# Prevalence of HPV Infection Among Chinese Males With HPV‐Related Diseases: A Systematic Review and Meta‐Analysis

**DOI:** 10.1002/jmv.70469

**Published:** 2025-07-10

**Authors:** Zexin Tao, Zhi Li, Baoli Chen, Xingxing Zhang, Rui Bian, Christine Velicer, Aiqiang Xu, Xiaodong Sun

**Affiliations:** ^1^ Shandong Center for Disease Control and Prevention Jinan China; ^2^ Institute of Immunization, Shanghai Municipal Center for Disease Control and Prevention Shanghai China; ^3^ Value & Implementation Global Medical & Scientific Affairs, MSD China Shanghai China; ^4^ Global Medical and Scientific Affairs, Merck & Co. Inc. Rahway New Jersey USA; ^5^ Shanghai Municipal Center for Disease Control and Prevention Shanghai China

**Keywords:** China, HPV, male, prevalence, systematic review

## Abstract

The prevalence and impact of HPV infection among Chinese males have not been comprehensively explored. This systematic review and meta‐analysis aims to investigate the prevalence of HPV infection among Chinese males diagnosed with HPV‐related diseases. A systematic search was conducted across multiple electronic databases from inception to June 2023. Studies reporting the prevalence of HPV infection among Chinese males diagnosed with diseases potentially associated with HPV, without requiring sample type documentation, were included. Data extraction and quality assessment were performed independently by two reviewers. A random‐effects meta‐analysis was used to estimate the pooled prevalence of HPV infection. Among 105 studies on genital warts, the pooled prevalence was 81.25% for any HPV type, with 55.84% for low‐risk (LR) genotypes and 20.11% for high‐risk (HR) genotypes. Single infections (53.93%) were most common, with HPV 6 and 11 being the most prevalent. Among the LR genotypes, the prevalence was 73.76% for HPV 6 or 11. The most prevalent HR genotypes were HPV 16, 52, 58, 18, and 51. Among 102 studies on cancers, the prevalence was 10.83%, 28.97%, 28.14%, 19.50%, and 45.01% for oropharyngeal cancer, oral cancer, tonsil cancer, laryngeal cancer, and penile cancer, respectively. For anal cancer, one study reported 87.5% were HPV‐positive. This study highlights the prevalence of HPV genotypes 6, 11, 16, 18, 52, and 58 in males with genital warts, and the prevalence of HPV 16 and 18 in males with cancers in which HPV has been detected. Future investment in resources focused on male is needed.

## 1 Introduction

Human papillomavirus (HPV) infection is one of the most common sexually transmitted infections globally, affecting individuals of all genders. Over 200 genotypes have been identified, some of which have been confirmed to possess carcinogenic potential. HPV genotypes are typically categorized into high‐risk (HR) and low‐risk (LR) types according to their carcinogenic potential [1]. In female, persistent HR‐HPV infection can lead to cervical cancer, a leading cause of global morbidity and mortality [2]. Much attention has been focused on HPV infection among females, although HPV‐related diseases in males have gained increasing recognition in recent years.

Estimates for male from 2018 suggest that HPV infection has been implicated in nearly all cases of anal squamous cell carcinoma, 52.9% of penile carcinoma, 30.9% of oropharyngeal carcinoma, 2.4% of laryngeal cancer, and 2.1% of oral cavity cancer [3]. The latest global estimate in 2023 suggests that nearly one in three males worldwide are infected with at least one genital HPV type (31%), and around one in five males carry at least one HR‐HPV type (21%) [4]. HPV also contributes to other associated diseases, such as genital warts and recurrent respiratory papillomatosis (RRP) [5, 6]. Studies report that 90% of genital warts and RRP cases are associated with HPV 6 and 11 [7]. Additionally, HPV infection has been detected in a subset of esophageal and lung cancers, although its causal role in these cancers remains inconclusive [8, 9]. HPV contributes significantly to the disease burden in males by increasing morbidity, reducing quality of life, and imposing substantial economic and psychosocial impacts [10, 11, 12, 13]. In case of cancers in which HPV has been detected, it also contributes to increased mortality rates [11]. Additionally, as HPV is sexually transmitted, infection in males can also lead to significant morbidity and mortality in females [14].

Despite the significant burden of diseases among males in which HPV has been detected, there remains a lack of comprehensive data in China, with greater public awareness of the impact of HPV on females [15, 16]. A 2017 systematic review and meta‐analysis in China indicated HPV prevalence rate from genital and anal swabs as 14.5% in male who have sex with male and female and 59.9% in male who have sex with men (MSM) [17]. However, among the 274 articles included, only 18 provided male‐specific data, highlighting the focus on females in the literature. Furthermore, the review did not address HPV prevalence among males with diseases potentially associated with HPV. A more recent systematic review estimated the prevalence of anal HPV among MSM in China to be 85.1% and 53.6% among HIV‐positive and HIV‐negative males, respectively [18]. However, the authors did not summarize the HPV prevalence at other anatomical sites.

Our review aims to summarize the prevalence of HPV among males with diseases in which HPV has been detected in China to both enhance the knowledge of healthcare professionals and the public and improve awareness of prevention, particularly through HPV vaccination.

## 2 Methods

This study followed the Preferred Reporting Items for Systematic Reviews and Meta‐analyses (PRISMA 2020) extension statement [19]. The protocol was registered on Prospero (registration number: CRD42023483562).

### 2.1 Data Sources and Literature Search

An electronic database search of PubMed, Embase, the Cochrane Library, Web of Science (WoS), China National Knowledge Infrastructure (CNKI), Wanfang, China Biology Medicine Database (CBM), and VIP was conducted on June 9, 2023 using the following keywords: Papillomaviridae or Human Papillomavirus Viruses, Male, Morbidity or Epidemiology, and China. No restrictions were applied on date/time, language, or document type. The search strategy is provided in Supporting Information S1: Supplementary S1.

### 2.2 Study Selection

We included observational and cross‐sectional studies investigating the prevalence of HPV infection among males with diseases in which HPV has been detected, including cancer, precancerous lesions, genital warts, or RRP, as defined in the original studies. Notably, all identified articles that met the inclusion criteria were included in our analysis, even though a clear causal link with HPV infection has not been confirmed for some types of cancer. According to WHO position paper [20], 12 HPV types are defined as high‐risk: (types 16, 18, 31, 33, 35, 39, 45, 51, 52, 56, 58, 59). HPV prevalence refers to the proportion of male who tested positive for HPV DNA, confirmed through PCR testing. As sample types (e.g., swabs vs. biopsies) were not always specified, results should be interpreted accordingly. We excluded case reports, therapeutic studies, reviews, meta‐analyses, and studies focused on clinical diagnostic methods. Studies not reported in English or Chinese were also excluded. Two reviewers (Baoli Chen and Zexin Tao) independently screened articles for eligibility, with any disagreements resolved through discussion with a third reviewer (Zhi Li).

### 2.3 Data Extraction

Two reviewers (Baoli Chen and Zexin Tao) independently extracted the following data from each study into a predefined data extraction form: study characteristics (first author name and publication year, study design, provinces, and sample size), participant characteristics (age, disease, diagnosis, detection methods and samples, sexual orientation, and HIV infection), and HPV‐related outcome data (number of samples testing positive for any HPV genotype, any high‐risk (HR) or low‐risk (LR) HPV genotype, single infection, double infection, and multiple infection, and attribution of HPV genotypes to cancer development). Any disagreements were resolved by discussion with a third reviewer (Zhi Li).

### 2.4 Data Analysis

Meta‐analyses were performed using a random‐effects model with the meta package in R software (version 4.1.1) to account for the potential high heterogeneity across population samples, study methodologies, and HPV DNA detection methods. We extracted the reported HPV prevalence estimates for each study, from which we calculated the HPV type combination‐specific prevalence (e.g., “any HR type”, etc.) and 95% confidence interval (CI), and we pooled these prevalence estimates by meta‐analysis. Before pooling the prevalence estimates, the Freeman–Tukey double arcsine transformation was employed to stabilize the variance of the raw prevalence observed in each included study. When data were insufficient for meta‐analysis (i.e., fewer than three studies for a particular combination of endpoint and HPV type combination), we calculated the prevalence by dividing the number of infected patients by the total sample size of the available studies.

The heterogeneity of studies was assessed using the Cochrane Q test with a significance level of 0.05 and the *I* ^2^ statistic, where *I* ^2^ ≥ 50% coupled with *p* < 0.05 from the Q test was interpreted as evidence of substantial heterogeneity [21]. In such cases, we provided a descriptive narrative of the outcomes. Additionally, subgroup analyses based on publication year were conducted to explore heterogeneity. The rising proportion of multiple infections in recent years may be due to changes in sexual behavior and improvements in HPV testing. Therefore, outcomes were analyzed by time period: within 10 years, 10–20 years ago, and over 20 years ago. Egger's test and Begg's test was conducted, funnel plots were created for outcomes with data from 10 or more studies to evaluate publication bias. A *p* < 0.05 in Egger's test and Begg's test indicated the presence of significant publication bias. When the number of included studies was small, the validity of the Begg's test was relatively reduced [22], so when the results of the two test were inconsistent, we reported the results with reference to Egger's test.

### 2.5 Quality Assessment

The quality of the included studies was assessed independently by two reviewers (Baoli Chen and Zexin Tao) using Joanna Briggs Institute (JBI) critical appraisal tools for prevalence studies [23]. Nine questions were used to assess sample representation, appropriateness of recruitment, sample size, detailed description of subjects and setting, sufficient data analysis, use of standard criteria, reliability of condition measurement, appropriateness of statistical analysis, and identification of confounding factors. Any disagreements were resolved by discussion with a third reviewer (Zhi Li).

## 3 Results

### 3.1 Results of Study Selection

**Figure 1:**
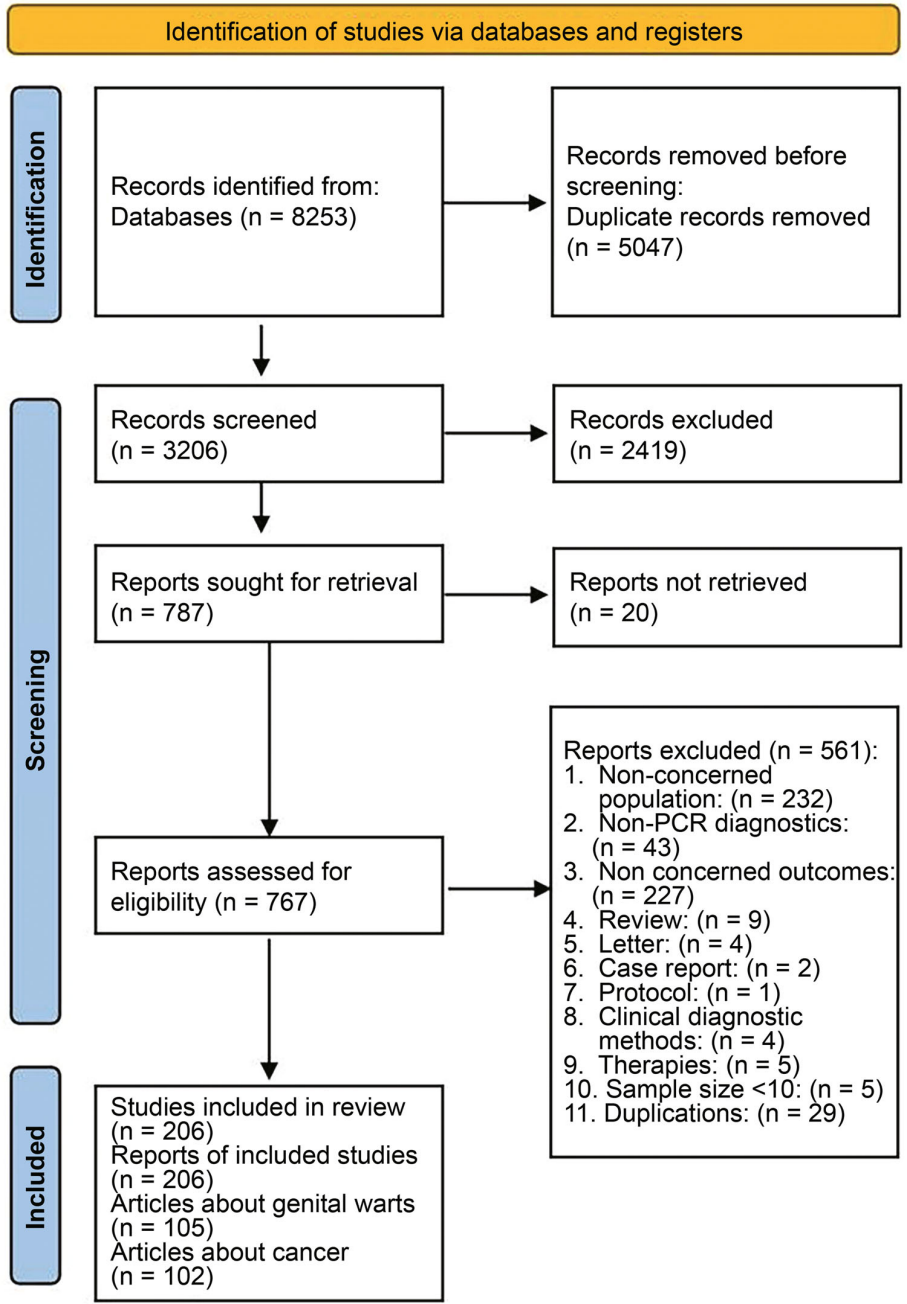
PRISMA flow chart of study selection.

The database search yielded 8253 results, with 3206 unique citations remaining after duplicate removal. A total of 787 articles were identified for retrieval based on their titles and abstracts meeting the inclusion criteria. With authorized access, 767 full publications were obtained for further review. Ultimately, 206 studies met our inclusion criteria and were included in this review (Figure 1), 105 of which had genital warts data and 102 had cancer/precancer data (with 1 study containing both genital warts and cancer/precancer). No studies assessed the relative contribution of HPV to the genital warts or precancers. Reference list is provided in Supporting Information S1: Supplementary S2.

### 3.2 Quality Assessment of Included Studies

All of the 105 studies reporting genital warts demonstrated strengths in sample representation, appropriate recruitment, data analysis, use of standard criteria, reliability of condition measurement and identification of confounding factors (100%). Most studies provided detailed descriptions of the subjects and settings (95%) and conducted data analysis with comprehensive coverage of the identified sample (84%). However, notable weaknesses were identified regarding the adequacy of the sample size (61%) (Supporting Information S1: Table S1). Similarly, the assessment of the 102 studies reporting cancer and precancer revealed strengths across most aspects but highlighted a significant weakness in sample size adequacy (23%) (Supporting Information S1: Table S2).

### 3.3 Characteristics of Studies and Patients

Of the 206 studies included, 105 focused on patients with genital warts, 102 examined patients with cancer and precancer, and one study contributed data for both genital warts and cancer [24]. In the studies focusing on genital warts, the sample size varied from 12 to 5717, while in the studies concerning cancer, the sample size ranged from 10 to 1,197. Detailed summaries of the studies and patients are given in Supporting Information S1: Table S3 for genital warts and in Supporting Information S1: Table S4 for cancer and precancer.

### 3.4 Prevalence of HPV Infection in Patients With Genital Warts

**Figure 2:**
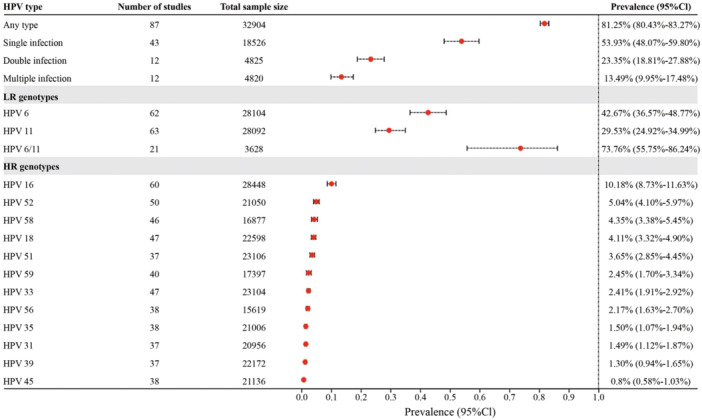
Prevalence of specific HPV types among male patients with genital warts.

The pooled prevalence of HPV infection of any genotype among patients diagnosed with genital warts was 81.25% (95% CI = 80.43%–83.27%, Figure 2), aggregated from 87 studies involving 32,904 patients. In addition, the prevalence was 55.84% (95% CI = 47.60%–63.92%) for any LR genotype and 20.11% (14.93%–25.84%) for any HR genotype. Results indicated that 53.93% of HPV‐infected male patients had a single infection (95% CI = 48.07%–59.80%, 43 studies with 18 526 patients), 23.35% had double infections (95% CI = 18.81%–27.88%, 12 studies with 4825 patients), and 13.49% had multiple infections (95% CI = 9.95%–17.48%, 12 studies with 4820 patients). Among patients with a single infection, the most prevalent genotypes were HPV 6 (25.30%, 95% CI = 20.96%–29.65%), followed by HPV 11 (19.04%, 95% CI = 15.08%–23.01%), HPV 16 (4.53%, 95% CI = 2.90%–6.15%), HPV 18 (1.41%, 95% CI = 0.51%–2.31%), and HPV 31 (1.09%, 95% CI = 0.35%–3.41%). Among the patients with double infections, the prevalences of infection with two LR genotypes, two HR genotypes, and one LR genotype plus one HR genotype were 3.96%, 6.71%, and 9.67%, respectively.

Regarding the genotypes targeted by vaccines, the prevalence was 42.67% (95% CI = 36.57%–48.77%) for HPV 6, 29.53% (95% CI = 24.92%–34.99%) for HPV 11, and 73.76% (95% CI = 55.75%–86.24%) for HPV6/11. Among the 12 HR HPVs detected in the included studies, the most prevalent types were HPV 16 (10.18%, 95% CI = 8.73%–11.63%), HPV 52 (5.04%, 95% CI = 4.10%–5.97%), HPV 58 (4.35%, 95% CI = 3.38%–5.45%), HPV 18 (4.11%, 95% CI = 3.32%–4.90%), and HPV 51 (3.65%, 95% CI = 2.85%–4.45%).

As the results of the meta‐analysis showed a high level of heterogeneity between studies, we explored whether publication time was responsible for the high level of heterogeneity through subgroup analyses. However, the results showed that grouping in this way did not reduce heterogeneity. Subgroup analysis also revealed no significant differences (*p* > 0.05, Supporting Information S1: Figures S1‐S5) in the prevalence of any HPV type, single, multiple, double infections, or HPV types 6/11 across the three time periods (within 10 years, 10–20 years ago, and over 20 years ago).

Publication bias detection results showed that any type of HPV, HPV 6, HPV 16, HPV 18, HPV 33, HPV 35, HPV 39, HPV 45, HPV 51, HPV 52, HPV 56, HPV 58, and HPV 59 in genital warts were in higher likelihood of publication bias in the included studies (Egger's test *p* < 0.05). Single infection, double infection, multiple infection, HPV 6/11, HPV 11, and HPV 31 may not be subject to publication bias (Egger's test and Begg's test *p* > 0.05). (Figures S6‐S24).

### 3.5 Prevalence of HPV Infection in Patients With Cancer and Precancerous Lesions

**Figure 3:**
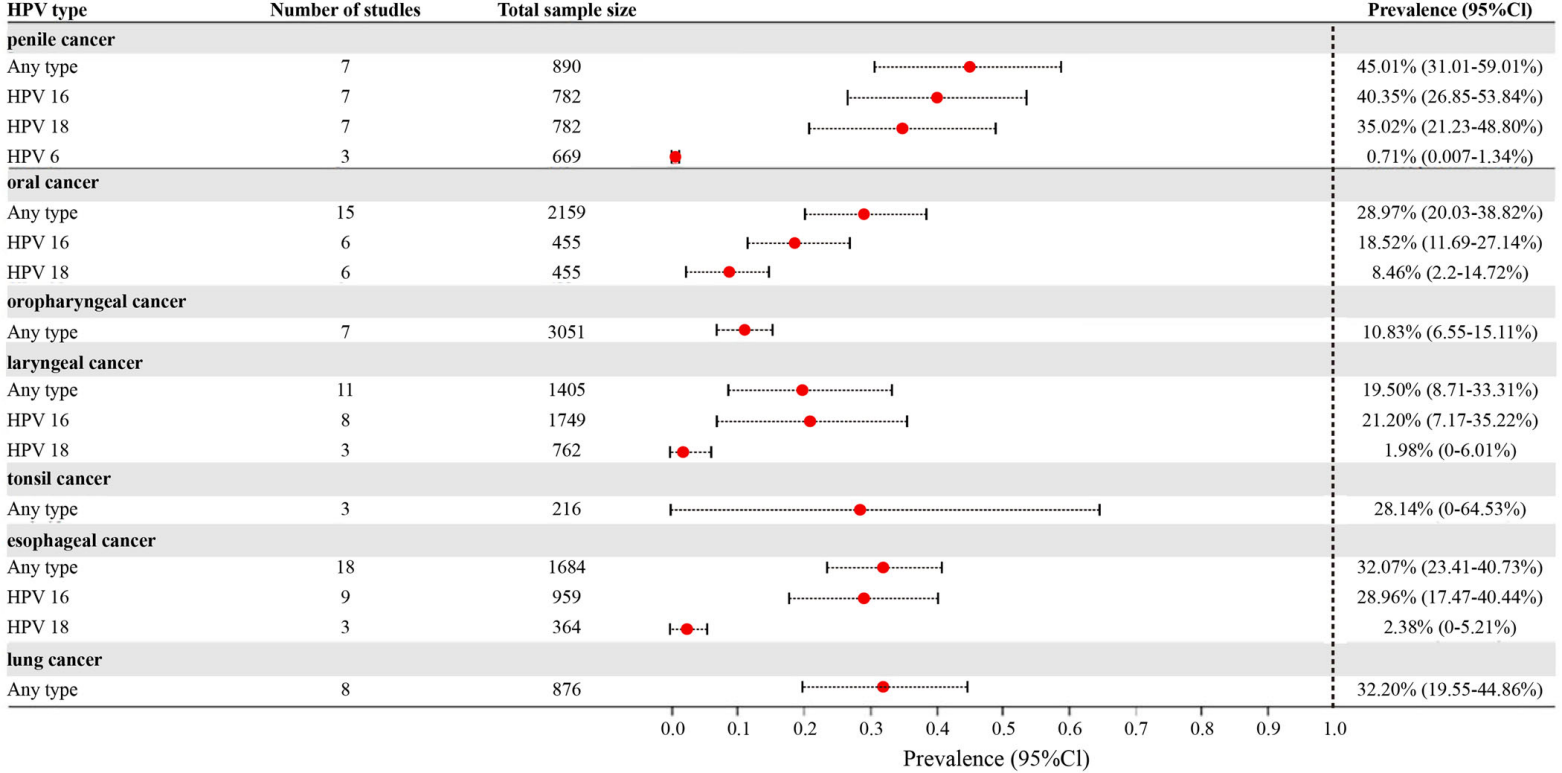
Prevalence of specific HPV types among male patients with HPV‐related cancers.

Among the 102 studies that provided data on patients with cancer, meta‐analyses were performed to pool the prevalence of HPV infection for patients with seven types of cancers (Figure 3).

#### 3.5.1 Anal Cancer and Precancer

In one study [24], seven of eight patients with anal cancer were tested for HPV, and all were found to be positive. For anal precancerous lesions [24, 25, 26, 27], the prevalence of HPV among 163 patients across four studies ranged from 92.86% to 100.0%.

#### 3.5.2 Penile Cancer and Precancer

Eight studies involving 900 patients with penile cancer revealed a prevalence of HPV infection of any type of 45.01% (95% CI = 31.01%–59.01%), with a pooled prevalence of 40.35% (95% CI = 26.85%–53.84%) for HPV 16, 35.02% (95% CI = 21.23%–48.80%) for HPV 18, and 0.71% (95% CI = 0.01%–1.34%) for HPV 6. One study [28] reported a prevalence of 100.0% among 10 patients with precancerous lesions of penile cancer.

#### 3.5.3 Head and Neck Cancers

Among the patients with head and neck cancers, the pooled prevalence of HPV infection of any type was 22.65% (95% CI = 17.59%–28.16%, 41 studies with 7520 patients). For oropharyngeal cancer, the pooled prevalence of HPV infection of any type was 10.83% (95% CI = 6.55%–15.11%, seven studies with 3051 patients). For laryngeal cancer, the meta‐analysis of 11 studies with 1405 patients gave a pooled prevalence of HPV infection of any type of 19.50% (95% CI = 8.71%–33.31%). Specifically, the prevalence of HPV 16 and 18 was 21.20% (95% CI = 7.17%–35.22%) and 1.98% (95% CI = 0.00%–6.01%), respectively. One study [29] reported that the prevalence of HPV infection of any type for precancerous lesions of the larynx was 73.33% among 15 patients. Another study [30] involving 110 patients with larynx cancer and hypopharynx cancer reported a prevalence rate of 16.36% for any type. The pooled prevalence of HPV infection of any type for tonsil cancer [31, 32, 33] was 28.14% (95% CI = 0.00%–64.53%, three studies with 216 patients). One study reported the prevalence of HPV infection of any type was 20.48% in patients with tongue cancer [34].

For oral cancer patients, the pooled prevalence of HPV infection of any type was 28.97% (95% CI = 20.03%–38.82%, 15 studies with 2159 patients). Specifically, the prevalence of HPV 16 and 18 among these patients was 18.52% (95% CI = 11.69–27.14%) and 8.46% (95% CI = 2.20%–14.72%), respectively. For precancerous lesions of oral cancer, the prevalence of HPV infection was 24.58%, calculated from two studies [35, 36] with 179 patients.

For nasopharyngeal carcinoma, one study [37] reported a prevalence of HPV infection of any type of 27.78%. For pharyngeal carcinoma, the prevalence of HPV infection of any type was 9.52%, based on a study [38] involving 168 patients. Additionally, the prevalence of HPV infection of any type was reported to be 8.33% in patients with nasal cancer [39] and 78.57% in patients with ear cancer [40].

#### 3.5.4 Other Cancers

Meta‐analysis was also performed for esophageal cancer and lung cancer. For esophageal cancer [41, 42, 43, 44, 45, 46, 47, 48, 49, 50, 51, 52, 53, 54, 55, 56, 57, 58], the prevalence of HPV infection was 32.07% (95% CI = 23.41%–40.73%) for any type, 28.96% (95% CI = 17.47%–40.44%) for HPV 16, and 2.38% (95% CI = 0.00%–5.21%) for HPV 18. For lung cancer [59, 60, 61, 62, 63, 64, 65, 66], the prevalence of HPV infection was 32.20% (95% CI = 19.55%–44.86%) for any type. Additionally, the prevalence of any type was 62.50% for bladder cancer [67], 16.51% for prostate cancer [68, 69], and 42.31% for gastric cancer [70] (Supporting Information S1: Table S5).

Significant publicationchageal cancer (Egger's test *p* = 0.0004). No significant bias was found for laryngeal cancer (Egger's test *p* = 0.0552) and oral cancer (Egger's test *p* = 0.1713) (Supporting Information S1: Figures S25–S27).

## 4 Discussion

This systematic review and meta‐analysis revealed a concerning prevalence of HPV infection among Chinese males diagnosed with diseases in which HPV has been detected, including genital warts and cancers. Although RRP is also caused by HPV infection (i.e., primarily HPV 6 and 11), our search did not reveal data for China. This highlights a significant gap in the current research and suggests a clear direction for future studies. Specifically, our findings indicated that among patients with genital warts, HPV 6 and 11 were the most prevalent LR genotypes, and HPV 16, 52, 58, 18, and 51 were the most prevalent HR genotypes. Among patients with cancers, HPV 16 and 18 were the most prevalent genotypes for most types of cancers, highlighting the critical role of these HPV types in cancers known to be caused by HPV in this population and underscoring the need for effective prevention and control measures.

Our study reveals the presence of multiple HPV infections for genital warts, alongside a notable prevalence of HR genotypes, highlights a significant shift in the epidemiology of HPV‐related diseases. Our findings reveal that double and multiple infections now account for nearly 37% of genital warts, with HR genotypes such as HPV 16, 52, and 58 becoming increasingly prevalent. This warrants attention, as co‐infections involving HR types are associated with a higher risk of persistent infection and progression to malignancy, particularly in the context of anogenital and oropharyngeal cancers [71]. These changes may underscore the advantages of multivalent vaccines, which offer broader protection by targeting a wider range of prevalent HPV genotypes. Additionally, our analysis highlights a notable prevalence of HPV infection among male patients with various cancers, including anal cancer (87.5%), penile cancer (45.01%), and oropharyngeal cancer (10.83%), esophageal cancer (32.07%), lung cancer (32.20%). The high prevalence observed among Chinese males emphasize the potential health benefits of expanding HPV vaccination programs to include males, thereby reducing the overall burden of diseases in which HPV has been detected.

Globally, oropharyngeal cancer rates are increasing, especially in males, with the US seeing an annual age‐standardized incidence rate of 6.41/100,000, compared to 0.39/100,000 in China [72]. However, lack of comprehensive and robust HPV data for oropharyngeal cancer in China creates uncertainty about future trends. For example, in the US between 2000 and 2021, oropharyngeal cancer rates increased 117% in males, surpassing cervical cancer as the most common HPV‐related cancer [73, 74]. It is hypothesized that the rising oropharyngeal cancer rates are due to changes in sexual behavior [75], while the decreasing cervical cancer trends are due to cervical screening, with rates expected to decrease even further as younger vaccinated cohorts age. This highlights a need for more research to understand these cancer trends in China.

Current HPV vaccines target HPV 16 and 18, which are commonly linked to HPV‐related cancers [76]. In addition, 4‐valent and 9‐valent vaccines provide coverage against genital warts and the 9‐valent vaccine provides protection against 5 additional cancer‐causing HPV types. The direct protection offered by HPV vaccines is crucial for reducing the incidence of HPV‐related diseases in males. Moreover, transmission of infection between partners can result in persistent infections, which heighten the risk of progressing to severe cervical abnormalities and, eventually, cervical cancer [77, 78, 79]. Increasing uptake of HPV vaccination among males may indirectly reduce the likelihood of HPV‐related diseases among females, such as cervical cancer. The most expedient approach to eliminating cervical cancer is gender‐neutral HPV vaccination, which can achieve this goal more quickly than a girls‐only strategy [80]. This approach is also resilient to disruptions, such as those caused by the COVID‐19 pandemic, and offers direct protection for both genders [80, 81]. An agent‐based model demonstrated that adopting a gender‐neutral strategy significantly improves resilience and enhances vaccination coverage, helping achieve WHO elimination thresholds by reducing cervical cancer incidence [81]. These findings provide valuable insights for China.

It is aware that some of the included studies used genotyping assays with limited HPV type coverage, which may have led to an underestimation of infection rates. However, the impact is likely minimal. As a retrospective analysis, it was not feasible to exclude studies based on the specific genotyping kits used.

Subgroup analysis based on publication year was also conducted to explore potential sources of heterogeneity. However, this analysis did not identify any clear patterns or explanations for the observed heterogeneity. One possible reason is that studies published in different time periods may vary significantly in terms of sample size, population characteristics, and methodological approaches. Additionally, the uneven distribution of studies across decades might have limited the statistical power of this analysis, making it difficult to draw firm conclusions. Therefore, while publication year was considered as a potential source of heterogeneity, it may not have fully captured the underlying differences between studies.

### 4.1 Strengths and Limitations

To our knowledge, this systematic review and meta‐analysis represents the first comprehensive summary of HPV prevalence among male patients with disease in which HPV has been detected in China. Through extensive search, rigorous analysis, and systematic synthesis, we provide valuable epidemiological insights. However, our study has limitations. First, studies on HPV in Chinese males, particularly for cancers, precancers, and other diseases, is limited [3]. Due to the limited number of studies for each cancer type, subgroup analysis was not feasible. Future large‐scale epidemiological studies are warranted to validate our findings. Globally, anal cancer accounts for approximately 0.5% of all new cancer cases, rising to 2.7% in the US [82, 83]. Yet China lacks specific prevalence data, with only one study on anal cancer in our review. In addition, data on age and sexual orientation are sparse, despite their influence on HPV prevalence and disease outcomes. Previous research suggests potential variations by age and geographical disparities in HPV prevalence among males across China [18]. Future studies could examine these factors in greater detail. Second, study heterogeneity exists in HPV types reported and sampling methods (e.g., swab exfoliated samples, biopsies, etc.), which may affect result accuracy. Moreover, the study lacks data on the relative contribution of specific HPV types to diseases in China. Prevalence estimates should not be interpreted as causal proportions, as other factors could contribute to these conditions. Identifying the specific HPV types involved and their proportionate contribution to cancers and precancers is essential for vaccination assessment. Some studies examined anatomical sites where HPV's causal role is speculative, like nasal and ear cancers, highlighting the need for further research using biomarkers or mRNA analysis. Some studies calculated the “any HPV type” rate, which includes individuals with multiple types, making direct comparisons with LR and HR rates inappropriate. This overlap affects the accuracy of summed prevalences and indicates a need for standardized reporting in future research. In addition, Egger's test and funnel plots revealed significant publication bias (*p* < 0.05) for certain HPV types in genital warts and cancers. This suggests that smaller studies with nonsignificant or negative findings may have been underrepresented, potentially leading to an overestimation of the prevalence of these HPV types. However, no significant bias was detected for other types, indicating relatively robust findings for these outcomes. Finally, due to limited relevant literature, we were unable to provide pooled HPV prevalence for certain types of cancers.

## 5 Conclusion

Our results highlight the noteworthy prevalence of HPV infection among male patients with HPV‐related diseases in China. HPV genotypes 6, 11, 16, 18, 52, and 58 were found to be prevalent among patients with genital warts. The findings also reveal gaps in the accurate reporting of the burden of HPV‐related cancers among Chinese males and a lack of comprehensive data on the HPV types driving these cancers. The study underscores the urgent need for greater resource allocation to address HPV‐related health issues in males. The widespread adoption of HPV multivalent vaccines in males holds clinical significance for preventing HPV‐related diseases in both males and females. Future studies could explore HPV infection prevalence among populations with different baselines.

## Author Contributions

Baoli Chen, Zhi Li, Xiaodong Sun and Aiqiang Xu, Rui Bian and Xingxing Zhang conceived, designed and planned the study; Baoli Chen and Zexin Tao collected the data; Baoli Chen, Zhi Li, Xiaodong Sun and Xingxing Zhang performed analysis; Zhi Li, Xiaodong Sun, Christine Velicer and Xingxing Zhang interpreted the results; Baoli Chen, Zexin Tao wrote the initial draft, Zhi Li, Xiaodong Sun, Aiqiang Xu, Christine Velicer and Xingxing Zhang provided substantive suggestions for revision or critically reviewed subsequent iterations of the manuscript. All authors reviewed and approved final version of the paper.

## Conflicts of Interest

Baoli Chen, Xingxing Zhang, and Rui Bian are employees of MSD China. Christine Velicer is an employee of Merck Sharp & Dohme LLC, a subsidiary of Merck & Co. Inc. Rahway, NJ, USA and may own stock and/or hold stock option in Merck & Co. Inc. Rahway, NJ, USA. All the other authors have no conflict of interest to declare.

## Supporting information

Supplementary materials‐clean.

## Data Availability

All data generated or analyzed during this study are included in this published article.
