# Supplementary material for: Prevalence of HPV Infection Among Chinese Males With HPV‐Related Diseases: A Systematic Review and Meta‐Analysis
Source: J Med Virol. 2025 Jul 10;97(7):e70469. doi: 10.1002/jmv.70469 (PMC12269541; doi:10.1002/jmv.70469)
Supplement: Supplementary file 1 — Supplementary materials‐clean. [file JMV-97-e70469-s001.docx]

**Supplementary 1 Search strategy**

Pubmed

1. "Papillomaviridae"[Mesh:noexp] OR "Human Papillomavirus Viruses"[Mesh] OR "Alphapapillomavirus"[Mesh] OR "HPV"[tw] OR "Human Papilloma Virus*"[tw] OR "Human Papillomavirus*"[tw] OR "Papilloma Virus*"[tw] OR "Papillomavirus*"[tw] OR "Alphapapillomavirus*"[tw]
2. "Male"[Mesh] OR male[tw] OR males[tw] OR Testicul*[tw] OR Testis*[tw] OR "Penile Neoplasms"[Mesh] OR Penis[tw] OR Penile[tw]
3. "Morbidity"[Mesh] OR "Epidemiology"[Mesh] OR "epidemiology" [Subheading] OR "Pharmacoepidemiology"[Mesh] OR Prevalenc*[tw] OR Incidenc*[tw] OR Attack Rate*[tw] OR Person-time Rate*[tw] OR "Epidemiolog*"[tw] OR "epidemiol*"[tw] OR "epidemic*"[tw] OR "Pharmacoepidemiolog*"[tw] OR "distribution*"[tw] OR "attribution*"[tw] OR "attributable fraction*"[tw] OR "relative contribution*"[tw] OR "coinfected lesion*"[tw] OR "multiple infection*"[tw] OR "fractional allocation*"[tw] OR "co infected lesion*"[tw] OR "Anus Neoplasms"[Mesh] OR Anal Cancer*[tw] OR Anus Cancer*[tw] OR "head and neck neoplasms"[MeSH] OR head cancer*[tw] OR neck cancer*[tw] OR throat cancer*[tw] OR ENT cancer*[tw] OR ORL cancer*[tw] OR otorhinolaryngeal cancer*[tw] OR otorhinolaryngologic cancer*[tw] OR otorhinolaryngological cancer*[tw] OR head Carcinoma*[tw] OR neck Carcinoma*[tw] OR penile intraepithelial neoplasia*[tw] OR Anal intraepithelial neoplasia*[tw] OR "Oropharyngeal Neoplasms"[Mesh] OR Oropharnyx Cancer*[tw] OR Oropharynx Cancer*[tw] OR Oropharnyx carcinoma*[tw] OR Oropharynx carcinoma*[tw] OR "Mouth Neoplasms"[Mesh] OR Mouth Cancer*[tw] OR Oral Cancer*[tw] OR oral carcinoma*[tw] OR "Condylomata Acuminata"[Mesh] OR condyloma*[tw] OR Venereal Wart*[tw] OR Genital Wart*[tw] OR "Recurrent respiratory papillomatosis" [Supplementary Concept] OR Recurrent respiratory papillomatos*[tw] OR JORRP[tw] OR AORRP[tw] OR genital lesions[tw] OR external genital lesions[tw] OR genital wart*[tw]
4. #1 and #2 and #3 12145
5. "China"[Mesh] OR China OR Chinese OR Taiwan OR Taiwanese OR "Hong kong" OR Hongkong OR Macau OR Macao OR Beijing OR Shanghai OR Tianjin OR Chongqing OR "Inner Mongolia" OR Tibet OR Guangxi OR Sinkiang OR Ningxia OR Xinjiang OR Hebei OR Shanxi OR Liaoning OR Jilin OR Heilongjiang OR Jiangsu OR Zhejiang OR Anhui OR Fujian OR Jiangxi OR Shandong OR Henan OR Hubei OR Hunan OR Guangdong OR Hainan OR Sichuan OR Guizhou OR Yunnan OR Shaanxi OR Gansu OR Qinghai
6. #4 and #5 841

EMBASE

1. 'Papillomaviridae'/de OR 'Alphapapillomavirus'/exp OR 'Wart virus'/exp OR (HPV OR "Human Papilloma Virus*" OR "Human Papillomavirus*" OR "Human Papillomavirus*" OR "Papilloma Virus*" OR Papillomavirus* OR Alphapapillomavirus*):ab,ti,kw
2. 'male'/exp OR 'male genital tract tumor'/exp OR 'male genital system disease'/exp OR (male OR males OR Testicul* OR Testis* OR Penis OR Penile):ab,ti,kw
3. 'morbidity'/exp OR 'epidemiology'/exp OR 'pharmacoepidemiology'/exp OR 'epidemiology'/lnk OR 'anus cancer'/exp OR 'head and neck cancer'/exp OR 'condyloma acuminatum'/exp OR 'recurrent respiratory papillomatosis'/exp OR (Morbidit* OR Prevalenc* OR Incidenc* OR "Attack Rate*" OR "Person-time Rate*" OR Epidemiolog* OR epidemiol* OR epidemic* OR Pharmacoepidemiolog* OR distribution* OR attribution* OR "attributable fraction*" OR "relative contribution*" OR "coinfected lesion*" OR "multiple infection*" OR "fractional allocation*" OR "co infected lesion*" OR ((Anal OR Anus OR head OR neck OR throat OR ENT OR ORL OR otorhinolaryngeal OR otorhinolaryngologic OR otorhinolaryngological OR Oropharnyx OR Oropharynx OR Mouth OR Oral) NEAR/3 (cancer* OR carcinoma*)) OR "penile intraepithelial neoplasia*" OR "Anal intraepithelial neoplasia*" OR condyloma* OR "Venereal Wart*" OR "Genital Wart*" OR "Recurrent respiratory papillomatos*" OR JORRP OR AORRP OR "genital lesions" OR "external genital lesions" OR "genital wart*"):ab,ti,kw
4. #1 and #2 and #3 18544
5. 'China'/exp OR (China OR Chinese OR Taiwan OR "Hong kong" OR Hongkong OR Macau OR Macao OR Beijing OR Shanghai OR Tianjin OR Chongqing OR "Inner Mongolia" OR Tibet OR Guangxi OR Sinkiang OR Ningxia OR Xinjiang OR Hebei OR Shanxi OR Liaoning OR Jilin OR Heilongjiang OR Jiangsu OR Zhejiang OR Anhui OR Fujian OR Jiangxi OR Shandong OR Henan OR Hubei OR Hunan OR Guangdong OR Hainan OR Sichuan OR Guizhou OR Yunnan OR Shaanxi OR Gansu OR Qinghai):ti,ab,ad,ff
6. #4 and #5 1221

Wos

1. TS=(HPV OR "Human Papilloma Virus*" OR "Human Papillomavirus*" OR "Human Papillomavirus*" OR "Papilloma Virus*" OR Papillomavirus* OR Alphapapillomavirus*)
2. TS=(male OR males OR Testicul* OR Testis* OR Penis OR Penile)
3. TS=(Morbidit* OR Prevalenc* OR Incidenc* OR "Attack Rate*" OR "Person-time Rate*" OR Epidemiolog* OR epidemiol* OR epidemic* OR Pharmacoepidemiolog* OR distribution* OR attribution* OR "attributable fraction*" OR "relative contribution*" OR "coinfected lesion*" OR "multiple infection*" OR "fractional allocation*" OR "co infected lesion*" OR ((Anal OR Anus OR head OR neck OR throat OR ENT OR ORL OR otorhinolaryngeal OR otorhinolaryngologic OR otorhinolaryngological OR Oropharnyx OR Oropharynx OR Mouth OR Oral) NEAR/3 (cancer* OR carcinoma*)) OR "penile intraepithelial neoplasia*" OR "Anal intraepithelial neoplasia*" OR condyloma* OR "Venereal Wart*" OR "Genital Wart*" OR "Recurrent respiratory papillomatos*" OR JORRP OR AORRP OR "genital lesions" OR "external genital lesions" OR "genital wart*")
4. #1 and #2 and #3 3780
5. CU=China OR TS=(China OR Chinese OR Taiwan OR "Hong kong" OR Hongkong OR Macau OR Macao OR Beijing OR Shanghai OR Tianjin OR Chongqing OR "Inner Mongolia" OR Tibet OR Guangxi OR Sinkiang OR Ningxia OR Xinjiang OR Hebei OR Shanxi OR Liaoning OR Jilin OR Heilongjiang OR Jiangsu OR Zhejiang OR Anhui OR Fujian OR Jiangxi OR Shandong OR Henan OR Hubei OR Hunan OR Guangdong OR Hainan OR Sichuan OR Guizhou OR Yunnan OR Shaanxi OR Gansu OR Qinghai) OR AD=(China OR Chinese OR Taiwan OR "Hong kong" OR Hongkong OR Macau OR Macao OR Beijing OR Shanghai OR Tianjin OR Chongqing OR "Inner Mongolia" OR Tibet OR Guangxi OR Sinkiang OR Ningxia OR Xinjiang OR Hebei OR Shanxi OR Liaoning OR Jilin OR Heilongjiang OR Jiangsu OR Zhejiang OR Anhui OR Fujian OR Jiangxi OR Shandong OR Henan OR Hubei OR Hunan OR Guangdong OR Hainan OR Sichuan OR Guizhou OR Yunnan OR Shaanxi OR Gansu OR Qinghai)
6. #4 and #5 251

Cochrane

#1 MeSH descriptor: [Papillomaviridae] explode all trees 926

#2 MeSH descriptor: [Papillomaviridae] explode all trees 926

#3 MeSH descriptor: [Alphapapillomavirus] explode all trees 392

#4 (HPV OR "Human Papilloma Virus*" OR "Human Papillomavirus*" OR "Human Papillomavirus*" OR "Papilloma Virus*" OR Papillomavirus* OR Alphapapillomavirus*):ti,ab,kw 4161

#5 #1 OR #2 OR #3 OR #4 4179

#6 MeSH descriptor: [Male] explode all trees 548381

#7 MeSH descriptor: [Penile Neoplasms] explode all trees 33

#8 (male OR males OR Testicul* OR Testis* OR Penis OR Penile):ti,ab,kw 853895

#9 #6 OR #7 OR #8 853895

#10 MeSH descriptor: [Morbidity] explode all trees 25910

#11 MeSH descriptor: [Epidemiology] explode all trees 386

#12 MeSH descriptor: [] explode all trees and with qualifier(s): [epidemiology - EP] 57647

#13 MeSH descriptor: [Pharmacoepidemiology] explode all trees 56

#14 (Morbidit* OR Prevalenc* OR Incidenc* OR "Attack Rate*" OR "Person-time Rate*" OR Epidemiolog* OR epidemiol* OR epidemic* OR Pharmacoepidemiolog* OR distribution* OR attribution* OR "attributable fraction*" OR "relative contribution*" OR "coinfected lesion*" OR "multiple infection*" OR "fractional allocation*" OR "co infected lesion*" OR ((Anal OR Anus OR head OR neck OR throat OR ENT OR ORL OR otorhinolaryngeal OR otorhinolaryngologic OR otorhinolaryngological OR Oropharnyx OR Oropharynx OR Mouth OR Oral) NEAR/3 (cancer* OR carcinoma*)) OR "penile intraepithelial neoplasia*" OR "Anal intraepithelial neoplasia*" OR condyloma* OR "Venereal Wart*" OR "Genital Wart*" OR "Recurrent respiratory papillomatos*" OR JORRP OR AORRP OR "genital lesions" OR "external genital lesions" OR "genital wart*"):ti,ab,kw 312378

#15 #10 or #11 OR #12 OR #13 OR #14 312379

#16 #5 and #9 and #15 726

#17 (China OR Chinese OR Taiwan OR "Hong kong" OR Hongkong OR Macau OR Macao OR Beijing OR Shanghai OR Tianjin OR Chongqing OR "Inner Mongolia" OR Tibet OR Guangxi OR Sinkiang OR Ningxia OR Xinjiang OR Hebei OR Shanxi OR Liaoning OR Jilin OR Heilongjiang OR Jiangsu OR Zhejiang OR Anhui OR Fujian OR Jiangxi OR Shandong OR Henan OR Hubei OR Hunan OR Guangdong OR Hainan OR Sichuan OR Guizhou OR Yunnan OR Shaanxi OR Gansu OR Qinghai) 128081

#18 #16 and #17 27，1篇review，26篇trials

#19 #16 not #18 699,1篇editorial，698篇trials

Cnki（期刊、学位、会议，中英文扩展：是，中文）

(SU%=(HPV+人乳头瘤病毒+人乳头状瘤病毒+人类乳头瘤病毒)*(阴茎肿瘤+阴茎癌+男性+阴茎+睾丸) OR TKA=(HPV+人乳头瘤病毒+人乳头状瘤病毒+人类乳头瘤病毒)*(阴茎肿瘤+阴茎癌+男性+阴茎+睾丸)) AND (TKA=患病率+发病率+感染率+传染率+流行病学+肛门癌+肛门腺腺癌+肛门生殖器癌+肛门直肠类癌+肛门周围癌+头颈癌+头颈部鳞状细胞癌+头颈部癌+头颈部恶性肿瘤+阴茎上皮内瘤变+肛门上皮内瘤变+口咽癌+口腔癌+湿疣+尖锐湿疣+复发性呼吸道乳头状瘤+JORRP+AORRP+外生殖器病变+生殖器病变+生殖器损伤+外生殖器疣+生殖器疣 OR SU%=患病率+发病率+感染率+传染率+流行病学+肛门癌+肛门腺腺癌+肛门生殖器癌+肛门直肠类癌+肛门周围癌+头颈癌+头颈部鳞状细胞癌+头颈部癌+头颈部恶性肿瘤+阴茎上皮内瘤变+肛门上皮内瘤变+口咽癌+口腔癌+湿疣+尖锐湿疣+复发性呼吸道乳头状瘤+JORRP+AORRP+外生殖器病变+生殖器病变+生殖器损伤+外生殖器疣+生殖器疣 OR ((SU%=基因型+分型+型别+高危型+亚型+HPV16+16型+"HPV 16"+HPV6+"HPV 6"+6型+HPV18+"HPV 18"+18型+HPV31+"HPV31"+31型+HPV33+"HPV33"+33型+HPV35+"HPV 35"+35型+HPV39+"HPV 39"+39型+HPV45+"HPV 45"+45型+HPV51+"HPV 51"+51型+HPV52+"HPV 52"+52型+HPV56+"HPV 56"+56型+HPV58+"HPV 58"+58型+ HPV59+ "HPV 59"+59型+ HPV68+"HPV 68"+68型+ HPV73+"HPV 73"+ 73型+HPV82+"HPV 82"+82型+单一感染+多重感染+二重感染 OR TKA=基因型+分型+型别+高危型+亚型+HPV16+16型+"HPV 16"+HPV6+"HPV 6"+6型+HPV18+"HPV 18"+18型+HPV31+"HPV31"+31型+HPV33+"HPV33"+33型+HPV35+"HPV 35"+35型+HPV39+"HPV 39"+39型+HPV45+"HPV 45"+45型+HPV51+"HPV 51"+51型+HPV52+"HPV 52"+52型+HPV56+"HPV 56"+56型+HPV58+"HPV 58"+58型+ HPV59+ "HPV 59"+59型+ HPV68+"HPV 68"+68型+ HPV73+"HPV 73"+ 73型+HPV82+"HPV 82"+82型+单一感染+多重感染+二重感染) AND (TKA=分布 OR TI=分布 OR SU%=分布))) 1012

万方（期刊、学位、会议）

主题:(HPV OR 人乳头瘤病毒) and 主题:(阴茎 OR 睾丸 OR 男性) and ((主题:("基因型" OR "分型" OR "型别" OR "高危型" OR "亚型" OR "HPV16" OR "HPV 16" OR "16型" OR "HPV6" OR "HPV 6" OR "6型" OR "HPV18" OR "HPV 18" OR "18型" OR "HPV31" OR "HPV 31" OR "31型" OR "HPV33" OR "HPV 33" OR "33型" OR "HPV35" OR "HPV 35" OR "35型" OR "HPV39" OR "HPV 39" OR "39型" OR "HPV45" OR "HPV 45" OR "45型" OR "HPV51" OR "HPV 51" OR "51型" OR "HPV52" OR "HPV 52" OR "52型" OR "HPV56" OR "HPV 56" OR "56型" OR "HPV58" OR "HPV 58" OR "58型" OR "HPV59" OR "HPV 59" OR "59型" OR "HPV68" OR "HPV 68" OR "68型" OR "HPV73" OR "HPV 73" OR "73型" OR "HPV82" OR "HPV 82" OR "82型" OR "单一感染" OR "多重感染" OR "二重感染") and 主题:(分布)) OR 主题:("患病率" OR "发病率" OR "感染率" OR "传染率" OR "流行病学" OR 肛门癌 OR 肛门腺腺癌 OR 肛门生殖器癌 OR 肛门直肠类癌 OR 肛门周围癌 OR 头颈癌 OR 头颈部鳞状细胞癌 OR 头颈部癌 OR 头颈部恶性肿瘤 OR "阴茎上皮内瘤变" OR "肛门上皮内瘤变" OR 口咽癌 OR 口腔癌 OR "湿疣" OR "复发性呼吸道乳头状瘤" OR JORRP OR AORRP OR "生殖器病变" OR "生殖器损伤" OR "生殖器疣")) 620

Cbm

( "HPV"[常用字段:智能] OR "人乳头瘤病毒"[常用字段:智能] OR "人乳头状瘤病毒"[常用字段:智能] OR "人类乳头瘤病毒"[常用字段:智能]) AND ( "阴茎肿瘤"[常用字段:智能] OR "阴茎癌"[常用字段:智能] OR "男性"[常用字段:智能] OR "阴茎"[常用字段:智能] OR "睾丸"[常用字段:智能]) AND (( "患病率"[常用字段:智能] OR "发病率"[常用字段:智能] OR "感染率"[常用字段:智能] OR "传染率"[常用字段:智能] OR "流行病学"[常用字段:智能] OR "肛门癌"[常用字段:智能] OR "肛门腺腺癌"[常用字段:智能] OR "肛门生殖器癌"[常用字段:智能] OR "肛门直肠类癌"[常用字段:智能] OR "肛门周围癌"[常用字段:智能] OR "头颈癌"[常用字段:智能] OR "头颈部鳞状细胞癌"[常用字段:智能] OR "头颈部癌"[常用字段:智能] OR "头颈部恶性肿瘤"[常用字段:智能] OR "阴茎上皮内瘤变"[常用字段:智能] OR "肛门上皮内瘤变"[常用字段:智能] OR "口咽癌"[常用字段:智能] OR "口腔癌"[常用字段:智能] OR "湿疣"[常用字段:智能] OR "尖锐湿疣"[常用字段:智能] OR "复发性呼吸道乳头状瘤"[常用字段:智能] OR "JORRP"[常用字段:智能] OR "AORRP"[常用字段:智能] OR "外生殖器病变"[常用字段:智能] OR "生殖器病变"[常用字段:智能] OR "生殖器损伤"[常用字段:智能] OR "外生殖器疣"[常用字段:智能] OR "生殖器疣"[常用字段:智能]) OR ( "分布"[常用字段:智能] AND ( "基因型"[常用字段:智能] OR "分型"[常用字段:智能] OR "型别"[常用字段:智能] OR "高危型"[常用字段:智能] OR "亚型"[常用字段:智能] OR "HPV16"[常用字段:智能] OR "HPV 16"[常用字段:智能] OR "16型"[常用字段:智能] OR "HPV6"[常用字段:智能] OR "HPV 6"[常用字段:智能] OR "6型"[常用字段:智能] OR "HPV18"[常用字段:智能] OR "HPV 18"[常用字段:智能] OR "18型"[常用字段:智能] OR "HPV31"[常用字段:智能] OR "HPV 31"[常用字段:智能] OR "31型"[常用字段:智能] OR "HPV33"[常用字段:智能] OR "HPV 33"[常用字段:智能] OR "33型"[常用字段:智能] OR "HPV35"[常用字段:智能] OR "HPV 35"[常用字段:智能] OR "35型"[常用字段:智能] OR "HPV39"[常用字段:智能] OR "HPV 39"[常用字段:智能] OR "39型"[常用字段:智能] OR "HPV45"[常用字段:智能] OR "HPV 45"[常用字段:智能] OR "45型"[常用字段:智能] OR "HPV51"[常用字段:智能] OR "HPV 51"[常用字段:智能] OR "51型"[常用字段:智能] OR "HPV52"[常用字段:智能] OR "HPV 52"[常用字段:智能] OR "52型"[常用字段:智能] OR "HPV56"[常用字段:智能] OR "HPV 56"[常用字段:智能] OR "56型"[常用字段:智能] OR "HPV58"[常用字段:智能] OR "HPV 58"[常用字段:智能] OR "58型"[常用字段:智能] OR "HPV59"[常用字段:智能] OR "HPV 59"[常用字段:智能] OR "59型"[常用字段:智能] OR "HPV68"[常用字段:智能] OR "HPV 68"[常用字段:智能] OR "68型"[常用字段:智能] OR "HPV73"[常用字段:智能] OR "HPV 73"[常用字段:智能] OR "73型"[常用字段:智能] OR "HPV82"[常用字段:智能] OR "HPV 82"[常用字段:智能] OR "82型"[常用字段:智能] OR "单一感染"[常用字段:智能] OR "多重感染"[常用字段:智能] OR "二重感染"[常用字段:智能]))) 652

维普 (由于检索式太长无法得出结果所以做了检索策略的缩减)

(M=(HPV+人乳头瘤病毒+人乳头状瘤病毒+人类乳头瘤病毒)*(阴茎肿瘤+阴茎癌+男性+阴茎+睾丸) OR R=(HPV+人乳头瘤病毒+人乳头状瘤病毒+人类乳头瘤病毒)*(阴茎肿瘤+阴茎癌+男性+阴茎+睾丸)) AND (U=患病率+发病率+感染率+传染率+流行病学+肛门癌+肛门腺腺癌+肛门生殖器癌+肛门直肠类癌+肛门周围癌+头颈癌+头颈部鳞状细胞癌+头颈部癌+头颈部恶性肿瘤+阴茎上皮内瘤变+肛门上皮内瘤变+口咽癌+口腔癌+湿疣+尖锐湿疣+复发性呼吸道乳头状瘤+JORRP+AORRP+外生殖器病变+生殖器病变+生殖器损伤+外生殖器疣+生殖器疣+((基因型+分型+型别+高危型+亚型+单一感染+多重感染+二重感染)*分布)) 659

**Supplementary 2 References**

1. Liang AF, He Y, J,, Hua XL, Lei LY, Sui H. Characteristics of human papillomavirus infection in 513 patients with condyloma acuminatum. *Experimental and Laboratory Medicine.* 2017;35(06):974-976.

2. Yang AQ, Bian KP, Zhai W, Zhai YR. HPV genotypes, epidemiological characteristics and outcomes of 227 patients with condyloma acuminatum in Nanyang area. *Practical Preventive Medicine.* 2018;25(09):1102-1104.

3. Shen B, Tu QF. Study on the relationship between human papillomavirus detection and clinical recurrence in patients with condyloma acuminatum. *Chinese Journal of AIDS & STD.* 2007(05):457-459.

4. Fang BX, Chen HQ, Qin ZH, et al. Analysis of human papilloma virus subtype distribution in 551 male patients with condyloma acuminatum. *China Medicine and Pharmacy.* 2018;8(17).

5. Chang L, Ci P, Shi J, et al. Distribution of genital wart human papillomavirus genotypes in China: a multi-center study. *J Med Virol.* 2013;85(10):1765-1774.doi:10.1002/jmv.23646.

6. Chen D. *Epidemiology and clinical study of HIV with tuberculosis infection and HIV with HPV infection* [master], Peking Union Medical College; 2011.

7. Chen X, Li L, Lai Y, Liu Q, Yan J, Tang Y. Characteristics of human papillomaviruses infection in men with genital warts in Shanghai. *Oncotarget.* 2016;7(33):53903-53910.doi:10.18632/oncotarget.9708.

8. Wang CD. Detection of human papilloma virus infection in patients with condyloma acuminatum. *Journal of Bengbu Medical College.* 2010;35(2).

9. Zeng CL, Zhu JF, Zhu GN, et al. Comparison of HPV genotype in different gender among 8876 patients with condyloma acuminatum. *China journal of Leprosy and Skin Diseases.* 2020;36(05):272-274.

10. Zhao CX. *HPV analysis in healthy men and patients with condyloma acuminatum* [Master], Qingdao University; 2009.

11. Cong X, Sun R, Zhang X, Wang Y, Wang L, Yu Y. Correlation of human papillomavirus types with clinical features of patients with condyloma acuminatum in China. *Int J Dermatol.* 2016;55(7):775-780.doi:10.1111/ijd.12964.

12. Dai Y, Huang YS, Tang M, Lv XP, Li TY, Yin YB. Distribution and clinical significance of human papillomavirus subtypes in Shenzhen city, People's Republic of China. *Int J Gynecol Cancer.* 2008;18(2):295-299.doi:10.1111/j.1525-1438.2007.01024.x.

13. Tan DY, Li X, Huang ZQ, Ou QX, Peng ZY. Clinical analysis of HPV genotypes detected by microarray in 142 patients with condyloma acuminatum. *Journal of Diagnosis and Therapy on Dermato-venereology.* 2009;16(03):166-169.

14. Fan F, Yin J, Xing JJ, Song YH, Duan YQ. HPV genotypes and clinical characteristics of male patients with condyloma acuminatum and risk factors for recurrence. *Chinese Journal of Nosocomiology.* 2023(11):1690-1694.

15. Hu FK, Deng YW, Du LP. Genotyping of human papillomavirus causing infections in patients with condyloma acuminate in anus and anal canal. *Chinese Journal of Nosocomiology.* 2014;24(13):3124-3126.

16. Li FG, Ding XP, Zhu YJ, Song Y, Zhang M. The research on the relationship between condyloma and human papillomavirus with the multiplex PCR technology. *Journal of Sichuan University（Natural Science Edition）.* 2011;48(01):225-230.

17. Chen GH, Zhao J, Ai YS, Liu DJ. Analysis of human papillomavirus infection in male patients with condyloma acuminatum. *Chinese Journal of Infectious Diseases.* 2018;36(05):277-279.

18. Zhang GQ, Su M, Zhu HY, Sun Y, Gao YH. Genotyping of human papillomavirus in sex workers with condyloma acuminatum in Kunming. *International Journal of Laboratory Medicine.* 2015;36(02):211-212.

19. Deng GH, Hu YX, WU X, Yang GL. Relationship between HPV virus subtypes of patients with genital warts and microecological. *Journal of Mudanjiang Medical University.* 2016;37(3).

20. Ning HM, Wu SF. Analysis of HPV genotypes in male patients with condyloma acuminatum in Lishui area. *National Journal of Andrology* 2015;21(11):1001-1004.doi:10.13263/j.cnki.nja.2015.11.009.

21. Hao S, Wang C, Liu S, He J, Jiang Y. HPV genotypic spectrum in Jilin province, China, where non-vaccine-covered HPV53 and 51 are prevalent, exhibits a bimodal agespecific pattern. *PloS one.* 2020;15(3)doi:10.1371/journal.pone.0230640.

22. Hu W, Shi Y, Guan MM, et al. An Analysis of HPV Infection and Distribution in Cervical and Genital Samples With Condyloma Acuminatum: A Retrospective Study. *International Journal of Dermatology and Venereology.* 2023;6(1):35-39.doi:10.1097/jd9.0000000000000200.

23. Liang HQ, Li Y, Chen JY, Ouyang ZB. The study of HPV infection type and HPV6 gene polymorphism in patients with condyloma acuminatum in Jiangmen area. *Medical laboratory and clinical.* 2013(2). Published 20131001.

24. Wang JB, Li XP, Zeng WY, et al. Investigation of fingertip HPV infection in patients with condyloma acuminatum in STD clinic. *Journal of Modern Laboratory Medicine.* 2018;33(03):148-150.

25. Wang JL, Zhu T, Han Y, Wang Y, Wen CM, Wei J. Human papillomavirus genotype distribution and recurrence risk factors in male patients with condyloma acuminatum. *Journal of Capital Medical University.* 2023;44(01):148-153.

26. Han JR. *Genotyping of human papillomavirus HPV in condyloma acuminata skin lesions in Ningxia* [Master], Ningxia Medical University; 2016.

27. Pu J, Yang T, Zahng B. A 3-year analysis of human papillomavirus infection in patients with condyloma acuminatum. *Journal of Nanjing Medical University (Natural Science).* 2013;33(09):1305-1307.

28. Liu JF, Guan CP, Tang X, Sun XK, Xu AE. Epidemiological investigation of human papillomavirus in venereal disease clinics, Hangzhou, Zhejiang. *China Preventive Medicine.* 2011;12(09):751-754.doi:10.16506/j.1009-6639.2011.09.023.

29. Xu JJ, Shu GB, Lu JJ, Xie HY, Shi LM. Detection of HPV genotypes in male patients with condyloma acuminatum in Beilun District of Ningbo City from 2014 to 2017. *China Preventive Medicine.* 2018;19(12):913-916.doi:10.16506/j.1009-6639.2018.12.008.

30. Zhang JH, Geng JX, Fan ZM, et al. Genetic analysis of HPV infection in 257 cases of genital condyloma acuminatum of anus and anal canal. *Chinese Journal of Clinical and Experimental Pathology.* 2013;29(05):520-523.doi:10.13315/j.cnki.cjcep.2013.05.006.

31. Tan J, Xu SM, Cai HB, Cao YY, Zhou QX, Zhang HP. Analysis of HPV microarray type in male condyloma acuminatum. *The Chinese Journal of Dermatovenereology.* 2012;26(03):196-199.

32. Wang J, Li J. Epidemiological analysis of human papillomavirus infection in male patients with condyloma acuminatum in Shanghai from 2017 to 2018. *Journal of Diagnostics Concepts & Practice.* 2020;19(06):572-576.doi:10.16150/j.1671-2870.2020.06.005.

33. Zhang J, Huang YC, Xue S, Yang MP, Xie J. Correlation between HPV subtypes and recurrence in male patients with condyloma acuminatum. *Jiangxi Medical Journal.* 2017;52(09):827-830.

34. Wang KD, Xu DJ, Su JR. Analysis of human papillomavirus genotypes in 1074 male external genital exfoliated cells in STD clinic. *Chinese Journal of Laboratory Medicine.* 2016;39(08):625-628.

35. Li LY, Sun Y, Xu XH, et al. Analysis of risk factors for recurrence of perianal and anal condyloma acuminatum among men who have sex with men. *Chinese Journal of AIDS & STD.* 2017;23(06):542-545.doi:10.13419/j.cnki.aids.2017.06.19.

36. Li LY, Zhu YJ, Ning Z, Jin NJ, Zhuang MH, Zheng H. Correlations between human papillomavirus types and recurrence of perianal and anal condyloma acuminatum among men who have sex with men. *Chinese Journal of Dermatology.* 2012;45(5). Published 20120830.

37. Lei YJ, Gao C, Wang C, et al. Molecular epidemiological study on prevalence of human papillomaviruses in patients with common warts in Beijing area. *Biomed Environ Sci.* 2009;22(1):55-61.doi:10.1016/s0895-3988(09)60023-4.

38. Pei LH, Tu XB, Liu SX, Jiang XL, Lan YB. Analysis of HPV genotypes in male condyloma acuminatum in Lishui area. *Chinese Journal of Health Laboratory Technology.* 2013;23(13):2764-2765.

39. Jiang LB, Tian XX, Li YW, et al. Analysis of 2830 male patients suspected of HPV infection in Shandong Province. *Chinese Journal of Human Sexuality.* 2018;27(04):37-40.

40. Feng LY, Liu QJ, Chen YY. Reverse dot hybridization analysis of epidemiological characteristics of 4620 HPV infected men in Zhejiang Province. *Chinese Journal of Clinical Laboratory Management（Electronic Editon）.* 2019;7(01):26-30.

41. Wang Q. Human papillomavirus DNA detection in urine samples of male patients with condyloma acuminatum. *Inertnational Journal of dermatology And Venereology.* 1999(01):63-64.

42. Ma DM, Sun MX, Li XY, et al. Distribution of high-risk human papillomavirus genotypes in male attendees at a clinic for sexually transmitted infections in Northern China. *Eur Rev Med Pharmacol Sci.* 2019;23(22):9714-9720.doi:10.26355/eurrev_201911_19533.

43. Xu M, Qiao L, Tian RL. Study on the relationship between male HPV infection and female vulvar and cervical HPV infection. *Medical Information.* 2017;30(16):181-182.

44. Zhao M, Zhang XL, Qiu HY, et al. Analysis of HIV with HPV infection and its subtypes in MSM population. *Chinese Journal of AIDS & STD.* 2013;19(04):239-240+247.doi:10.13419/j.cnki.aids.2013.04.019.

45. Liu MY, Wang QP, Han CY, Han CF, Ren M. Clinical case report of condyloma acuminatum in Jinan area -- Discussion of 336 suspected cases. *sexology.* 1998(04):46.

46. Zhao M, Cai JL, Zhou S, et al. Subtype detection and epidemiological analysis of genital HPV infection. *Chinese Journal of AIDS & STD.* 2010;16(03):281-283.doi:10.13419/j.cnki.aids.2010.03.042.

47. Jin N, Liu WL, Yang L, Liang JZ, Han Y. Analysis of Human Papillomavirus DNA Genotyping in High Risk Sexually Transmitted Diseases Group. *The Chinese Journal of Dermatovenereology.* 2007(08):490-491.

48. Zahng N. *Analysis of human papillomavirus gene subtypes in 904 patients with condyloma acuminatum* [Master], Tianjin Medical University; 2018.

49. Song NH, Wu HF, Qian LX, Hua LX, Song NJ, Tang NF. Application of human papillomavirus detection in male patients with condyloma acuminatum. Paper presented at: Sixth National Conference on Medical Genetics2005; Beijing, China.

50. Ding Q, Zhang YF, Sun X. Detection of human papilloma virus by polymerase chain reaction (PCR). *Journal of Andrology.* 1996(02):6-8.

51. Li Q, Gao YW, Wang HQ, et al. [Human papillomavirus infection in men with condyloma acuminatum: Genotype analysis of 70 cases]. *Zhonghua Nan Ke Xue.* 2020;26(10):906-910. Published 2021/01/01.

52. Liao QH, Liang ZD, Meng X, Li LM, Xu QH. Fluorescence quantitative PCR and its application in detection of pathogen DNA of STD in man. *Chinese Journal of Andrology.* 2004(03):24-25+28.

53. Zhang Q, Xiong Y, Jiang YR, Feng L, Jiang X, Wang SP. Detection and genotyping of HPV in patients with condyloma acuminatum in Chongqing. *The Chinese Journal of Dermatovenereology.* 2018;32(10):1163-1166.doi:10.13735/j.cjdv.1001-7089.201712107.

54. Lin QL, Lin J, Zhao LH, Wang KJ, Wu Q, Yan HY. Analysis of human papilloma virus infection state and genotypes of Condyloma Acuminate by gene chip technology. *Modern Journal of Integrated Traditional Chinese and Western Medicine.* 2013;22(30):3325-3328.

55. Cheng S, Zhao SG, Zhao SK, Xie ML. HPV infection and clinical characteristics in 159 male patients with condyloma acuminatum in Liaocheng,Shandong. *China Tropical Medicine.* 2022:1-6.

56. Cheng S, Zhao SG, Zhao SK, Xie ML. HPV infection and clinical characteristics in 159 male patients with condyloma acuminatum in Liaocheng,Shandong. *China Tropical Medicine.* 2023;23(03):289-293.doi:10.13604/j.cnki.46-1064/r.2023.03.14.

57. Wu SF, Wang W, Chen H, Yu XM. Effect of sex factors on human papillomavirus genotypes in patients with condyloma acuminatum. *National Journal of Andrology* 2014;20(07):660-662.doi:10.13263/j.cnki.nja.2014.07.014.

58. Li SH, Yin WH, Tan YF, Zhu XY, Cao WK. Analysis of HPV genotypes in patients with condyloma acuminatumin Jiaxing. *Chinese Journal of Human Sexuality.* 2015;24(12):53-55.

59. Xu SM, Tan J, Cao YY, et al. Detection of HPV type in different parts of male condyloma acuminata skin lesions by gene chip. Paper presented at: The 18th National Dermatological and venereal Diseases Academic Conference of the Chinese Medical Association2012; 中国北京.

60. Tang X, Xu AE, Dong XP, Sun XK, Shen H, Liu JF. Epidemiological investigation of human papillomavirus infection in men attending a sexually transmitted disease clinic in Hangzhou area. *Biomed Environ Sci.* 2006;19(2):153-157. Published 2006/07/11.

61. Ting Y, Jie P, Bin Z, Ping X, Hejian S. Distribution and clinical significance of human papilloma virus genotypes in 100 male patients with condyloma acuminate. *Chinese Journal of Andrology.* 2010;24(6):40-43.

62. Tsai TF, Kuo GT, Kuo LT, Hsiao CH. Prevalence status and association with human papilloma virus of anal squamous proliferative lesions in a patient sample in Taiwan. *Sex Transm Dis.* 2008;35(8):721-724.doi:10.1097/OLQ.0b013e3181705878.

63. Ma WY, Zahng YC, Tao SJ, Y. S, Zhao KN. HPV-DNA typing of anorectal condyloma acuminata in male contact population. *China journal of Leprosy and Skin Diseases.* 2009;25(1):67-68.doi:10.3969/j.issn.1009-1157.2009.01.043.

64. Wang WG, Zheng LL, Sun Z. HPV typing analysis of 67 condyloma acuminatum patients. *Chinese Journal of Human Sexuality.* 2013;22(02):53-54+65.

65. Li WH, Jia J, Cai L, et al. Observation of the relapse rates of condyloma acuminatum with different human papillomavirus genotypes in male urethral meatus. *Chinese Journal of AIDS & STD.* 2016;22(10):813-816.doi:10.13419/j.cnki.aids.2016.10.14.

66. Zhuang WH, Xu SH, Wang ZS, Mao YB. Analysis of human papillomavirus infection among male patients in STD clinic in Fuzhou area. *Strait Journal of Preventive Medicine.* 2010;16(05):85-86.

67. Liu WH, Huan H, Xia CF. Relationship between subclinical human papilomavirus infection and recurrence of condyloma acuminatum. *China journal of Leprosy and Skin Diseases.* 2014;30(02):97-99.

68. Shan XN, Jiang Q, Ma XQ, et al. Detection of HPV DNA typing in condyloma acuminatum and papilloma of external auditory meatus by PCR. *Chinese Journal of Virology.* 1993;9(4).

69. Ma XJ, Jiang S, Xu LL, Chen GZ. Distribution of male human papillomavirus genotypes in Qingdao. *Progress in clinical medicine.* 2021;11(4).

70. Zhang XH, Huang HQ, Liu H, et al. Analysis of HPV infection types among men who have sex with men in Guangzhou. *Journal of Diagnosis and Therapy on Dermato-venereology.* 2015;22(04):285-289.

71. Wu XJ, Ren JG, Liu YJ, et al. Molecular detection of HPV virus in samples of condyloma acuminatum. *Hereditas.* 2005(05):699-704.doi:10.16288/j.yczz.2005.05.004.

72. Wang XL. *Epidemiological analysis of HPV infection in 79 patients with condyloma acuminatum* [Master], Tianjin Medical University; 2014.

73. Hu XP, Wang YQ, Ye R, Yu B, Zhong QL, 王燕. Type Detection and Analysis of Human Papillomavirus in 1619 Patients with Condyloma Acuminatum. *Chinese Journal of Dermatovenerology of Integrated Traditional and Western Medicine.* 2012;11(06):345-347.

74. Shen XX, Liu WM, Xiao SX, Jia J, Tian Y. Detection of urethral mucosa HPV6/11 DNA in male patients with condyloma acuminatum. *The Chinese Journal of Dermatovenereology.* 2013;27(10):1031-1033.

75. Shen XX, Liu WM, Xiao SX, Jia J, Tian Y. Detection of human papillomavirus type 6/11 DNA in warts and fingers of male patients with condyloma acuminatum. *China journal of Leprosy and Skin Diseases.* 2013;29(09):561-563.

76. Zhao XX, Fu XX, Wang QJ. Detection and typing of human papillomavirus in condyloma acuminatum tissue in Haikou area. *International Journal of Laboratory Medicine.* 2017;38(15):2125-2127.

77. Tian XX, Wang R, Jiang LB, Li YW, Qi DB. Analysis of HPV subtypes in 1225 male patients with condyloma acuminatum in Shandong province. *Chinese Journal of Andrology.* 2016;30(07):41-45.

78. Long XR, Geng JX, Li L, et al. Distribution of HPV infection genotypes in 176 male urethral meatus cells. *International Journal of Laboratory Medicine.* 2013;34(06):723-725.

79. Yu XJ, Jiang JM. Detection of HPV virus genotypes in male patients with condyloma acuminatum in Quzhou. *Chinese Journal of Health Laboratory Technology.* 2017;27(24):3607-3609.

80. Yao XQ, Liao XM, Tang Y, Zheng XL, Ceng HZ. Analysis of human papillomavirus genotypes in male patients with condyloma acuminatum in a certain area. *Guide of China Medicine.* 2015;13(13):17-18.doi:10.15912/j.cnki.gocm.2015.13.011.

81. Lu Y, Wang XL, Wu D, Dong ZB. [Clinical features and epidemiological survey of perianal warts in 72 males]. *Zhonghua Nan Ke Xue.* 2006;12(10):923-926. Published 2006/11/24.

82. Yao YB, Jiang LZ, Li ZM, et al. Molecular epidemiological analysis and clinical study of HPV infection in adult patients with condyloma acuminatum in Quanzhou City. *China Health Standard Management.* 2021;12(09):25-28.

83. Liu YH. *Genotyping of anorectal HPV in MSM population and analysis of related factors* [Master], Qingdao University; 2017.

84. Zhong YQ, Chen YQ, Xiao X, Chen JY, Shao JH, Zhang DX. Analysis of HPV infection rate and genotype distribution in male reproductive tract in Dongguan. *Experimental and Laboratory Medicine.* 2018;36(05):781-783.

85. Xue YH, Jiang HN, Zheng HP, Huang JM, Zeng WY, Wu XZ. Study on human papillomavirus infection and its genotyping in male patients with sexually transmitted diseases in Guangdong area. *Chinese Journal of Clinical Laboratory Science.* 2011;29(07):526-528.doi:10.13602/j.cnki.jcls.2011.07.044.

86. Yang Y, Cui F, Yong G, et al. Retrospective analysis of HPV genotypes in multiple infection lesions of condyloma acuminata. *Chinese Journal of AIDS & STD.* 2019;25(07):718-721.doi:10.13419/j.cnki.aids.2019.07.17.

87. Sun Y, Xu M, Zhu XH, Xu JH. Analysis of FQ-PCR results in 110 patients with condyloma acuminatum. *Chinese Journal of AIDS & STD.* 2008(04):387-388.

88. Xiong Y. Genotyping and clinical analysis of HPV in 108 patients with condyloma acuminatum. *Journal of Community Medicine.* 2007(17):16-17.

89. Qin Y, Yu ZR, Chen Z, CAI HR. Clinical characteristics and psychological evaluation of 64 cases of anal and rectal condyloma acuminatum in men who have sex with HIV. *Chinese Journal of Experimental and Clinical Virology.* 2019;13(02):117-121.

90. Tang Y, Tang XL. Analysis of HPV DNA typing results in patients with condyloma acuminatum and high-risk groups. *Laboratory Medicine and Clinic.* 2009;6(17):1446-1447.

91. Dai YG, Zheng W, Mu JK. Analysis of human papillomavirus genotypes in 269 males with condyloma acuminata in Luzhong area. *Journal of Clinical Dermatology.* 2019;48(08):467-469.doi:10.16761/j.cnki.1000-4963.2019.08.003.

92. Wang YB, Zhao CX, Han T. Distribution of HPV subtypes in pubic hair follicles and skin lesions in patients with condyloma acuminatum. *Journal of QingDao University(Medical Sciences).* 2010;46(04):360-361.

93. Yuan H, Li R, Lv J, et al. Epidemiology of human papillomavirus on condyloma acuminatum in Shandong Province，China. *Hum Vaccin Immunother.* 2023;19(1):2170662.doi:10.1080/21645515.2023.2170662.

94. Chen YY, Shi M, Qian YH, Zhou PY. The prevalence and genotypes of Human Papillomavirus in anal region condyloma acuminatum and oral area among men who have sex with men in Shanghai. *Chinese Journal of Andrology.* 2017;31(02):22-28.

95. Haung Y, Ji PL, Song L, Liu HT. Human papillomavirus detectionin exfoliated cells from male external genitalia. *Chinese Journal of Experimental and Clinical Virology.* 2015;29(03):280-283.

96. He YF, Liu LH, Yang WJ. Application of HPV-DNA genotyping in the detection and diagnosis of genital warts. *Chinese Journal of Human Sexuality.* 2015;24(03):56-58.

97. Gao Y, Ye H, Ke J. HPV infection status of 116 patients with condyloma acuminatum and epidemiological analysis. *Chinese Journal of Human Sexuality.* 2019;28(05):154-157.

98. Fu YP, Li DN, Yan TF, et al. Analysis of the Genotypes of Human Papilloma Virus (HPV) in the Lesions of Condyloma Acuminatum. *The Chinese Journal of Dermatovenereology.* 2018;32(02):150-153.doi:10.13735/j.cjdv.1001-7089.201707011.

99. Fu YP, Sun J, F., Yan TF, Li DN. Analysis of urethral HPV infection in male patients with condyloma acuminatum. *Beijing Medical Journal* 2020;42(10):948-950+954.doi:10.15932/j.0253-9713.2020.10.010.

100. Huang ZQ, Xie WX, Chen QR, et al. Detection and analysis of HPV genotyping in 223 cases of male condyloma acuminatum. *Guangdong Medical Journal.* 2014;35(07):1087-1089.doi:10.13820/j.cnki.gdyx.2014.07.046.

101. Huang ZQ, Yao SN, Li XP, et al. Prevalence and genotype distribution of HPV infection in male patients with condyloma acuminatum in Foshan region. *China Tropical Medicine.* 2018;18(09):923-926.doi:10.13604/j.cnki.46-1064/r.2018.09.17.

102. Chen ZY. *Relationship between recurrence of condyloma acuminatum and HPV genotyping* [Master], Shihezi University; 2016.

103. Dai ZR, Li D, Wang KL, Huang ZY, Ma XD. HPV infection and genotyping analysis of 500 male patients in STD clinic. *Guangdong Medical Journal.* 2016;37(17):2637-2639.doi:10.13820/j.cnki.gdyx.20160923.008.

104. Bao ZY, Zhang YJ, Zou XB, Guo XJ, Lin H. Analysis of HPV type characteristics in 109 patients with condyloma acuminatum. *Infectious Disease Information.* 2016;29(05):276-278+320.

105. Zhu C, Wang Y, Mao W, Zhang H, Ma J. Prevalence and distribution of HPV types in genital warts in Xi'an, China: a prospective study. *BMJ Open.* 2019;9(5):e023897.doi:10.1136/bmjopen-2018-023897.

106. Chen F, Yan L, Liu F, et al. Oral human papillomavirus infection, sexual behaviors and risk of oral squamous cell carcinoma in southeast of China: A case-control study. *J Clin Virol.* 2016;85:7-12.doi:10.1016/j.jcv.2016.10.011.

107. Wang L, Li J, Hou J, et al. p53 expression but not p16(INK4A) correlates with human papillomavirus-associated esophageal squamous cell carcinoma in Kazakh population. *Infect Agent Cancer.* 2016;11:19.doi:10.1186/s13027-016-0065-x.

108. Zhang DH, Zhang QY, Hong CQ, Chen JY, Shen ZY, Zhu Y. Prevalence and association of human papillomavirus 16, Epstein-Barr virus, herpes simplex virus-1 and cytomegalovirus infection with human esophageal carcinoma: a case-control study. *Oncol Rep.* 2011;25(6):1731-1738.doi:10.3892/or.2011.1234.

109. Tong F, Geng J, Yan B, et al. Prevalence and Prognostic Significance of HPV in Laryngeal Squamous Cell Carcinoma in Northeast China. *Cell Physiol Biochem.* 2018;49(1):206-216.doi:10.1159/000492858.

110. Liu B, Lu Z, Wang P, Basang Z, Rao X. Prevalence of high-risk human papillomavirus types (HPV-16, HPV-18) and their physical status in primary laryngeal squamous cell carcinoma. *Neoplasma.* 2010;57(6):594-600.doi:10.4149/neo_2010_06_594.

111. He D, Zhang DK, Lam KY, et al. Prevalence of HPV infection in esophageal squamous cell carcinoma in Chinese patients and its relationship to the p53 gene mutation. *Int J Cancer.* 1997;72(6):959-964.doi:10.1002/(sici)1097-0215(19970917)72:6<959::aid-ijc7>3.0.co;2-o.

112. Chu C, Chen K, Tan X, et al. Prevalence of human papillomavirus and implication on survival in Chinese penile cancer. *Virchows Arch.* 2020;477(5):667-675.doi:10.1007/s00428-020-02831-7.

113. Jin YT, Tsai ST, Li C, et al. Prevalence of human papillomavirus in middle ear carcinoma associated with chronic otitis media. *Am J Pathol.* 1997;150(4):1327-1333. Published 1997/04/01.

114. Hu C, Quan H, Yan L, Sun J, Lan L, Wang S. Prevalence of human papillomavirus in sinonasal squamous cell carcinoma with and without association of inverted papilloma in Eastern China. *Infect Agent Cancer.* 2020;15(1)doi:10.1186/s13027-020-00298-4.

115. He F, Xiong W, Yu F, et al. Human papillomavirus infection maybe not associated with primary lung cancer in the Fujian population of China. *Thorac Cancer.* 2020;11(3):561-569.doi:10.1111/1759-7714.13282.

116. Lee LA, Huang CG, Tsao KC, et al. Human Papillomavirus Infections are Common and Predict Mortality in a Retrospective Cohort Study of Taiwanese Patients With Oral Cavity Cancer. *Medicine (Baltimore).* 2015;94(47):e2069.doi:10.1097/md.0000000000002069.

117. Zhang ZY, Sdek P, Cao J, Chen WT. Human papillomavirus type 16 and 18 DNA in oral squamous cell carcinoma and normal mucosa. *Int J Oral Maxillofac Surg.* 2004;33(1):71-74.doi:10.1054/ijom.2002.0443.

118. Luo CW, Roan CH, Liu CJ. Human papillomaviruses in oral squamous cell carcinoma and pre-cancerous lesions detected by PCR-based gene-chip array. *Int J Oral Maxillofac Surg.* 2007;36(2):153-158.doi:10.1016/j.ijom.2006.09.005.

119. Gu W, Zhang P, Zhang G, et al. Importance of HPV in Chinese Penile Cancer: A Contemporary Multicenter Study. *Front Oncol.* 2020;10:1521.doi:10.3389/fonc.2020.01521.

120. Huang KS, Ni GY, Ma BW, et al. Importance of human papillomavirus infection in squamous cell carcinomas of the tongue in Guangdong Province, China. *Journal of International Medical Research.* 2020;48(1)doi:10.1177/0300060519897187.

121. Ma X, Sheng S, Wu J, et al. LncRNAs as an intermediate in HPV16 promoting myeloidderived suppressor cell recruitment of head and neck squamous cell carcinoma. *Oncotarget.* 2017;8(26):42061-42075.doi:10.18632/oncotarget.14939.

122. Chien CY, Su CY, Fang FM, et al. Lower prevalence but favorable survival for human papillomavirus-related squamous cell carcinoma of tonsil in Taiwan. *Oral Oncol.* 2008;44(2):174-179.doi:10.1016/j.oraloncology.2007.01.018.

123. Lang B, Dong D, Zhao T, et al. A cross-sectional study of human papillomavirus genotype distribution and integration status in penile cancer among Chinese population. *Virology.* 2023;584:53-57.doi:10.1016/j.virol.2023.04.013.

124. Chen B. [Expression of human papillomavirus and pRb in head and neck squamous cell carcinoma]. *Lin Chuang Er Bi Yan Hou Ke Za Zhi.* 2004;18(3):154-156. Published 2004/06/30.

125. Yang JQ, Wu M, Liu YF, et al. Original article correlation between hpv infection and tumor recurrence in patients with head and neck squmaous cell carcinoma in tangshan, china. *International Journal of Clinical and Experimental Medicine.* 2019;12(8):10689-10695.

126. Meng HX, Yang XX, Liu RQ, et al. The relationship between human papillomavirus, ofd1 and primary ciliogenesis in the progression of oropharyngeal cancer: A retrospective cohort study. *Pharmacogenomics and Personalized Medicine.* 2020;13((Meng H.-X., menghongxue15@163.com; Yang X.-X.; Hou Y.-J.) Department of Pathology, Harbin Medical University Cancer Hospital, Harbin, China):633-644.doi:10.2147/pgpm.s271735.

127. Lei L, Li H, Sun Y. [Study of HPV in oral squamous cell carcinoma]. *Zhonghua Kou Qiang Yi Xue Za Zhi.* 1996;31(6):375-377. Published 1996/11/01.

128. Chen XJ, Sun K, Jiang WW. Absence of high-risk HPV 16 and 18 in Chinese patients with oral squamous cell carcinoma and oral potentially malignant disorders. *Virol J.* 2016;13:81.doi:10.1186/s12985-016-0526-2.

129. Zhai JP, Wang QY, Wei D, Xu KX, Man LB. [Association between HPV DNA and disease specific survival in patients with penile cancer]. *Zhonghua Yi Xue Za Zhi.* 2013;93(34):2719-2722. Published 2013/12/24.

130. Huang SF, Li HF, Liao CT, et al. Association of HPV infections with second primary tumors in early-staged oral cavity cancer. *Oral Diseases.* 2012;18(8):809-815.doi:10.1111/j.1601-0825.2012.01950.x.

131. Huang L, Wu MG, He J, et al. [Correlation of high-risk HPV 16/18 infections with prostate cancer]. *Zhonghua Nan Ke Xue.* 2016;22(6):501-505. Published 2016/06/01.

132. Patima, Cao J, Chen WT, Zhang ZY. [Detection of high risk human papillomavirus DNA in oral squamous cell carcinoma]. *Shanghai Kou Qiang Yi Xue.* 2000;9(4):212-215. Published 2004/03/12.

133. Chen L, Dong P, Yu Z. Detection of human papillomavirus infection in laryngeal and hypopharyngeal carcinoma using droplet digital PCR and its correlation with prognosis. *Postgrad Med.* 2021;133(6):619-625.doi:10.1080/00325481.2021.1904669.

134. Wei W, Shi Q, Guo F, et al. The distribution of human papillomavirus in tissues from patients with head and neck squamous cell carcinoma. *Oncol Rep.* 2012;28(5):1750-1756.doi:10.3892/or.2012.1990.

135. Chen X, Gao L, Sturgis EM, et al. HPV16 DNA and integration in normal and malignant epithelium: implications for the etiology of laryngeal squamous cell carcinoma. *Ann Oncol.* 2017;28(5):1105-1110.doi:10.1093/annonc/mdx027.

136. Lu WH. *Effects of HPV-16 infection on biological behavior of laryngeal squamous cell carcinoma and FaDu cells and regulation of miRNAs expression* [Phd], Zhengzhou University; 2013.

137. Zhao N. *Epidemiological study of HPV (Human papillomavirus) in colorectal cancer* [Master], Zhejiang University; 2010.

138. Gao LM. *Correlation between HPV infection and the incidence and prognosis of head and neck squamous cell carcinoma* [Phd], Peking Union Medical College; 2020.

139. Min XL, Liu XH. Qualitative diagnostic value of HPV subtypes in laryngeal phosphoid cell carcinoma and precancerous lesions. *Journal of Tropical Medicine.* 2017;17(09):1160-1163.

140. Huang CC, Hsiao JR, Yang MW, et al. Human papilloma virus detection in neoplastic and non-neoplastic nasopharyngeal tissues in Taiwan. *J Clin Pathol.* 2011;64(7):571-577.doi:10.1136/jcp.2010.087742.

141. Wang Z, Xia RH, Ye DX, Li J. Human Papillomavirus 16 Infection and TP53 Mutation: Two Distinct Pathogeneses for Oropharyngeal Squamous Cell Carcinoma in an Eastern Chinese Population. *PloS one.* 2016;11(10):e0164491.doi:10.1371/journal.pone.0164491.

142. Zhao D, Xu QG, Chen XM, Fan MW. Human papillomavirus as an independent predictor in oral squamous cell cancer. *International journal of oral science.* 2009;1(3):119-125.doi:10.4248/ijos.09015.

143. Huang H, Zhang B, Chen W, et al. Human papillomavirus infection and prognostic predictors in patients with oropharyngeal squamous cell carcinoma. *Asian Pac J Cancer Prev.* 2012;13(3):891-896.doi:10.7314/apjcp.2012.13.3.891.

144. Xu Y, Liu S, Yi H, et al. Human papillomavirus infection in 674 Chinese patients with laryngeal squamous cell carcinoma. *PloS one.* 2014;9(12):e115914.doi:10.1371/journal.pone.0115914.

145. Chan KW, Lam KY, Chan ACL, Lau P, Srivastava G. PREVALENCE OF HUMAN PAPILLOMAVIRUS TYPE-16 AND TYPE-18 IN PENILE CARCINOMA - A STUDY OF 41 CASES USING PCR. *J Clin Pathol.* 1994;47(9):823-826.doi:10.1136/jcp.47.9.823.

146. Li S, Shen H, Li J, Hou X, Zhang K. Prevalence of the integration status for human papillomavirus 16 in esophageal carcinoma samples. *Turk J Gastroenterol.* 2018;29(2):157-163.doi:10.5152/tjg.2018.17568.

147. Lam EW, Chan JY, Chan AB, et al. Prevalence, Clinicopathological Characteristics, and Outcome of Human Papillomavirus-Associated Oropharyngeal Cancer in Southern Chinese Patients. *Cancer Epidemiol Biomarkers Prev.* 2016;25(1):165-173.doi:10.1158/1055-9965.epi-15-0869.

148. Liu T, Liu Q, Liang M, et al. Viral load of HPV 16/18 in esophageal squamous cell carcinoma in three ethnic groups living in Xinjiang Autonomous Region, China. *Mol Biol Rep.* 2013;40(2):2045-2052.doi:10.1007/s11033-012-2263-y.

149. Qu LJ, Wang H, Liu HL. Association of high-risk human papillomavirus with transitional cell carcinoma of the bladder in men. *Journal of Logistics University of PAP（Medical Sciences）.* 2010;19(05):357-359.

150. Ding Q, Zhang YF, Sun X. The role of polymerase chain reaction and nucleic acid hybridization in the detection of human papilloma virus in penile cancer. *Chinese Journal of Surgery.* 1996;34(1).

151. Xu YN, Du BL, Zhou XF, Xiong L. Epidemiological analysis of human papillomavirus infection in patients with cutaneous squamous cell carcinoma. *Journal of Clinical Medicine in Practice.* 2017;21(07):92-94.

152. Wang Z, Luo F. Detection of human papillomavirus types in skin squamous cell carcinoma. *Journal of Clinical Medicine in Practice.* 2014;7(01):10-12+15.

153. Long Y. *Preliminary investigation and gene variation analysis of high-risk human papilloma virus infection rate in 105 cases of head and neck squamous cell carcinoma and 59 cases of head and neck chronic inflammation* [Master], Luzhou Medical College; 2011.

154. Yan Y, Zhang H, Jiang C, et al. Human Papillomavirus Prevalence and Integration Status in Tissue Samples of Bladder Cancer in the Chinese Population. *J Infect Dis.* 2021;224(1):114-122.doi:10.1093/infdis/jiaa710.

155. Wang Y, Wang A, Jiang R, et al. Human papillomavirus type 16 and 18 infection is associated with lung cancer patients from the central part of China. *Oncol Rep.* 2008;20(2):333-339. Published 2008/07/19.

156. Hu JM, Li L, Chen YZ, et al. Human papillomavirus type 16 infection may be involved in esophageal squamous cell carcinoma carcinogenesis in Chinese Kazakh patients. *Dis Esophagus.* 2013;26(7):703-707.doi:10.1111/dote.12009.

157. Zhang QY, Zhang DH, Shen ZY, Xu LY, Li EM, Au WW. Infection and integration of human papillomavirus in esophageal carcinoma. *Int J Hyg Environ Health.* 2011;214(2):156-161.doi:10.1016/j.ijheh.2010.11.001.

158. Li S, Shen H, Liu Z, et al. Integration of human papillomavirus 16 in esophageal carcinoma samples. *Infect Agent Cancer.* 2017;12(1)doi:10.1186/s13027-017-0164-3.

159. Chen WC, Chuang HC, Lin YT, Huang CC, Chien CY. Clinical impact of human papillomavirus in laryngeal squamous cell carcinoma: A retrospective study. *PeerJ.* 2017;2017(5)doi:10.7717/peerj.3395.

160. Fan X, Yu K, Wu J, Shao J, Zhu L, Zhang J. Correlation between squamous cell carcinoma of the lung and human papillomavirus infection and the relationship to expression of p53 and p16. *Tumor Biology.* 2015;36(4):3043-3049.doi:10.1007/s13277-014-2940-7.

161. Cheng YW, Chiou HL, Chen JT, et al. Gender difference in human papillomarvirus infection for non-small cell lung cancer in Taiwan. *Lung Cancer.* 2004;46(2):165-170.doi:10.1016/j.lungcan.2004.03.023.

162. Cui X, Chen Y, Liu L, et al. Heterozygote of PLCE1 rs2274223 increases susceptibility to human papillomavirus infection in patients with esophageal carcinoma among the Kazakh populations. *J Med Virol.* 2014;86(4):608-617.doi:10.1002/jmv.23775.

163. Lu XM, Monnier-Benoit S, Mo LZ, et al. Human papillomavirus in esophageal squamous cell carcinoma of the high-risk Kazakh ethnic group in Xinjiang, China. *Eur J Surg Oncol.* 2008;34(7):765-770.doi:10.1016/j.ejso.2007.11.007.

164. Shuyama K, Castillo A, Aguayo F, et al. Human papillomavirus in high- and low-risk areas of oesophageal squamous cell carcinoma in China. *Br J Cancer.* 2007;96(10):1554-1559.doi:10.1038/sj.bjc.6603765.

165. Cheng SH, Liao KS, Wang CC, Cheng CY, Chu FY. Multiple types of human papillomavirus infection and anal precancerous lesions in HIV-infected men in Taiwan: a cross-sectional study. *BMJ Open.* 2018;8(1):e019894.doi:10.1136/bmjopen-2017-019894.

166. Li W, Tran N, Lee SC, et al. New evidence for geographic variation in the role of human papillomavirus in tonsillar carcinogenesis. *Pathology.* 2007;39(2):217-222.doi:10.1080/00313020701230823.

167. Lam KY, He D, Ma L, et al. Presence of human papillomavirus in esophageal squamous cell carcinomas of Hong Kong Chinese and its relationship with p53 gene mutation. *Hum Pathol.* 1997;28(6):657-663.doi:10.1016/s0046-8177(97)90174-x.

168. Al-Swiahb JN, Huang CC, Fang FM, et al. Prognostic impact of p16, p53, epidermal growth factor receptor, and human papillomavirus in oropharyngeal cancer in a betel nut-chewing area. *Arch Otolaryngol Head Neck Surg.* 2010;136(5):502-508.doi:10.1001/archoto.2010.47.

169. Meng HX, Miao SS, Chen K, et al. Association of p16 as Prognostic Factors for Oropharyngeal Cancer: Evaluation of p16 in 1470 Patients for a 16 Year Study in Northeast China. *Biomed Res Int.* 2018;2018:9594568.doi:10.1155/2018/9594568.

170. Cui L, Qu C, Liu H. Association study of cell cycle proteins and human papillomavirus in laryngeal cancer in Chinese population. *Clin Otolaryngol.* 2019;44(3):323-329.doi:10.1111/coa.13296.

171. Wu E, An G, Wu R, et al. Prevalence of human papillomavirus in archival head and neck cancer in the eastern Inner Mongolian Autonomous Region, China. *Tumori.* 2020;106(5):369-377.doi:10.1177/0300891620901768.

172. Cheng SH, Chu FY, Wang CC, Hsueh YM. Screening and risk factors for anal cancer precursors in men infected with HIV in Taiwan. *J Med Virol.* 2014;86(2):193-201.doi:10.1002/jmv.23825.

173. Sun ZQ, Wang HJ, Zhao ZL, Wang QS, Fan CW, Fang F. Significance of HPV infection and genic mutation of APC and K-ras in patients with rectal cancer. *Asian Pac J Cancer Prev.* 2013;14(1):121-126.doi:10.7314/apjcp.2013.14.1.121.

174. Kuo KT, Hsiao CH, Lin CH, Kuo LT, Huang SH, Lin MC. The biomarkers of human papillomavirus infection in tonsillar squamous cell carcinoma-molecular basis and predicting favorable outcome. *Mod Pathol.* 2008;21(4):376-386.doi:10.1038/modpathol.3800979.

175. Liu WK, Jiang XY, Zhang MP, Zhang ZX. The relationship between HPV16 and expression of cyclooxygenase-2, P53 and their prognostic roles in esophageal squamous cell carcinoma. *European Journal of Gastroenterology and Hepatology.* 2010;22(1):67-74.doi:10.1097/MEG.0b013e32832c7e76.

176. Chan JYK, Cheung MK, Lan L, et al. Characterization of oral microbiota in HPV and non-HPV head and neck squamous cell carcinoma and its association with patient outcomes. *Oral Oncol.* 2022;135((Chan J.Y.K., jasonchan@ent.cuhk.edu.hk; Lan L.; Ng C.; Lau E.H.L.; Yeung Z.W.C.; Wong E.W.Y.; Leung L.; Qu X.) Department of Otorhinolaryngology, Head and Neck Surgery, Faculty of Medicine, The Chinese University of Hong Kong, Hong Kong Special Administrative Region, China)doi:10.1016/j.oraloncology.2022.106245.

177. Ma TY, Liu WK, Chu YL, et al. Detection of human papillomavirus type 16 DNA in formalin-fixed, paraffin-embedded tissue specimens of gastric carcinoma. *European Journal of Gastroenterology and Hepatology.* 2007;19(12):1090-1096.doi:10.1097/MEG.0b013e3282eeb4dc.

178. Wang H, Zhang Z, Sun R, et al. HPV Infection and Anemia Status Stratify the Survival of Early T2 Laryngeal Squamous Cell Carcinoma. *J Voice.* 2015;29(3):356-362.doi:10.1016/j.jvoice.2014.08.016.

179. Yang SW, Lee YS, Chen TA, Wu CJ, Tsai CN. Human papillomavirus in oral leukoplakia is no prognostic indicator of malignant transformation. *Cancer Epidemiol.* 2009;33(2):118-122.doi:10.1016/j.canep.2009.05.003.

180. Ni G, Huang K, Luan Y, et al. Human papillomavirus infection among head and neck squamous cell carcinomas in southern China. *PloS one.* 2019;14(9):e0221045.doi:10.1371/journal.pone.0221045.

181. Chan PK, To KF, Tsang SH, Lau CH, Kwong WH, Chan YH. Human papillomavirus infection and squamous cell carcinoma in Hong Kong: a case-control study. *Hong Kong medical journal = Xianggang yi xue za zhi.* 2017;23(4):8-11.

182. Wang JL, Fang CL, Wang M, et al. Human papillomavirus infections as a marker to predict overall survival in lung adenocarcinoma. *Int J Cancer.* 2014;134(1):65-71.doi:10.1002/ijc.28349.

183. Lee LA, Huang CG, Tsao KC, et al. Increasing rates of low-risk human papillomavirus infections in patients with oral cavity squamous cell carcinoma: association with clinical outcomes. *J Clin Virol.* 2013;57(4):331-337.doi:10.1016/j.jcv.2013.04.010.

184. Wang YF, Wang XS, Gao SG, et al. Clinical significance of combined detection of human papilloma virus infection and human telomerase RNA component gene amplification in patients with squamous cell carcinoma of the esophagus in northern China. *Eur J Med Res.* 2013;18(1):11.doi:10.1186/2047-783x-18-11.

185. Chang JY, Lin MC, Chiang CP. High-risk human papillomaviruses may have an important role in non-oral habits-associated oral squamous cell carcinomas in Taiwan. *Am J Clin Pathol.* 2003;120(6):909-916.doi:10.1309/c5p6-nuq2-nw6l-ctbp.

186. Chiou HL, Wu MF, Liaw YC, et al. The presence of human papillomavirus type 16/18 DNA in blood circulation may act as a risk marker of lung cancer in Taiwan. *Cancer.* 2003;97(6):1558-1563.doi:10.1002/cncr.11191.

187. Zhang M, Wu CH, Wang YJ, Yuan YH, Yang Q. The relationship between HPV infection and the expression of insulin-like growth factor II in lung cancer and its clinical significance. *Tumor.* 2009;29(8):749-753.doi:10.3781/j.issn.1000-7431.2009.08.010.

188. Lam EWH, Chan MMH, Wai CKC, et al. The role of human papillomavirus in laryngeal cancer in Southern China. *J Med Virol.* 2018;90(6):1150-1159.doi:10.1002/jmv.25058.

189. Zhang HZ, Shan CG, Wang JM, Huang AP. Detection and typing of human papilloma virus in fresh frozen tissue of oropharyngeal squamous cell carcinoma. *Chinese Archives of Otolaryngology-Head and Neck Surgery.* 2016;23(5):291-292.doi:10.16066/j.1672-7002.2016.05.015.

190. Liu YL, Liu RQ, Chen HS. The role of human papillomavirus and oncogenes in the development of human genital carcinoma. *Journal of Clinical Dermatology.* 1995(04):216-218.

191. Liu L. *Study on the relationship between human papillomavirus (HPV) infection and MHC-Class Ⅰ molecular expression and the development of esophageal squamous cell carcinoma* [Master], Hebei Medical University; 2014.

192. Du XG. *Expression of P53 and P73 in esophageal carcinoma tissues and their correlation with HPV16/18 infection* [Master], Shanxi Medical University; 2010.

193. Zhuang YH. Study on serum levels of soluble tumor necrosis factor receptor and human papillomavirus infection in patients with penile cancer. *Journal of advanced medical education.* 2005(20):18-19+44.

194. Wang Z. *Clinicopathology, HPV16/18 and TP53 changes of pharyngeal squamous cell carcinoma in Chinese population* [Phd], Shanghai Jiao Tong University; 2015.

195. Cui LF, Qu CL, Zhao HY, Chang H, Gao Y, Liu HG. Clinical significance of HPV16 and p16 expression in laryngeal squamous cell carcinoma. *Chinese Journal of Cancer Prevention and Treatment.* 2021;28(9):661-667.doi:10.16073/j.cnki.cjcpt.2021.09.05.

196. Mao ML, Xing L, Yang DM, Yin HY, WAN HF, Liu HG. Clinicopathological analysis of spindle-cell squamous cell carcinoma of the larynx. *Chinese Journal of Clinical and Experimental Pathology.* 2018;34(7). Published 20181230.

197. Cui LF, Chang H, Zhao HY, Qu CL, Gao Y, Liu HG. [Significance of HPV and cell cycle related proteins in basaloid squamous cell carcinoma of the larynx]. *Zhonghua Bing Li Xue Za Zhi.* 2018;47(9):682-686.doi:10.3760/cma.j.issn.0529-5807.2018.09.006.

198. Chang HJ, Pong YH, Chiang CY, et al. A matched case-control study in Taiwan to evaluate potential risk factors for prostate cancer. *Sci Rep.* 2023;13(1):4382.doi:10.1038/s41598-023-31434-w.

199. Chuang CY, Sung WW, Wang L, et al. Differential Impact of IL-10 Expression on Survival and Relapse between HPV16-Positive and -Negative Oral Squamous Cell Carcinomas. *PloS one.* 2012;7(10)doi:10.1371/journal.pone.0047541.

200. Cheng SH, Wang CC, Chang SL, Chu FY, Hsueh YM. Oncogenic human papillomavirus is not helpful for cytology screening of the precursor lesions of anal cancers in Taiwanese men who are infected with human immunodeficiency virus. *Int J Clin Oncol.* 2015;20(5):943-951.doi:10.1007/s10147-015-0804-9.

201. Yu Y, Liu X, Yang Y, et al. Effect of FHIT loss and p53 mutation on HPV-infected lung carcinoma development. *Oncology Letters.* 2015;10(1):392-398.doi:10.3892/ol.2015.3213.

202. Wu MF, Cheng YW, Lai JC, et al. Frequent p16INK4a promoter hypermethylation in human papillomavirus-infected female lung cancer in Taiwan. *Int J Cancer.* 2005;113(3):440-445.doi:10.1002/ijc.20597.

203. Zhang D, Zhang W, Liu W, et al. Human papillomavirus infection increases the chemoradiation response of esophageal squamous cell carcinoma based on P53 mutation. *Radiotherapy and Oncology.* 2017;124(1):155-160.doi:10.1016/j.radonc.2017.06.008.

204. Wang WL, Wang YC, Lee CT, et al. The impact of human papillomavirus infection on the survival and treatment response of patients with esophageal cancers. *J Dig Dis.* 2015;16(5):256-263.doi:10.1111/1751-2980.12236.

205. Armas GL, Su CY, Huang CC, Fang FM, Chen CM, Chien CY. The impact of virus in N3 node dissection for head and neck cancer. *European Archives of Oto-Rhino-Laryngology.* 2008;265(11):1379-1384.doi:10.1007/s00405-008-0670-4.

206. Lo WY, Lai CC, Hua CH, et al. S100A8 is identified as a biomarker of HPV18-infected oral squamous cell carcinomas by suppression subtraction hybridization, clinical proteomics analysis, and immunohistochemistry staining. *J Proteome Res.* 2007;6(6):2143-2151.doi:10.1021/pr060551+.

**Supplementary 3 Quality assessment**

Table S1 Quality assessment of included studies providing data for patients with genital warts

| Study ID | *Was the sample representative of the target population?* | *Were study participants recruited in an appropriate way?* | *Was the sample size adequate?* | *Were the study subjects and setting described in detail?* | *Is the data analysis conducted with sufficient coverage of the identified sample?* | *Were objective, standard criteria used for measurement of the condition?* | *Was the condition measured reliably?* | *Was there appropriate statistical analysis?* | *Are all important confounding factors/ subgroups/differences identified and accounted for?* |
| --- | --- | --- | --- | --- | --- | --- | --- | --- | --- |
| Aifen Liang 2017^1^ | Yes | Yes | Yes | Yes | Yes | Yes | Yes | Yes | Yes |
| Aiqin Yang 2018^2^ | Yes | Yes | Yes | Yes | Yes | Yes | Yes | Yes | Yes |
| Bin Shen 2007^3^ | Yes | Yes | No | Yes | Yes | Yes | Yes | Yes | Yes |
| Bingxiong Fang 2018^4^ | Yes | Yes | Yes | Yes | Yes | Yes | Yes | Yes | Yes |
| Lihong Chang 2013^5^ | Yes | Yes | Yes | Yes | Yes | Yes | Yes | Yes | Yes |
| Dian Chen 2011^6^ | Yes | Yes | No | Yes | Yes | Yes | Yes | Yes | Yes |
| Xiaogang Chen 2016^7^ | Yes | Yes | Yes | Yes | Yes | Yes | Yes | Yes | Yes |
| Chenghu Wang 2010^8^ | Yes | Yes | No | Yes | Yes | Yes | Yes | Yes | Yes |
| Chenglong Zeng 2020^9^ | Yes | Yes | Yes | Yes | Yes | Yes | Yes | Yes | Yes |
| Chunxiong Zhao 2009^10^ | Yes | Yes | No | No | Yes | Yes | Yes | Yes | Yes |
| Xianling Cong 2016^11^ | Yes | Yes | No | Yes | Yes | Yes | Yes | Yes | Yes |
| Y. Dai 2008^12^ | Yes | Yes | No | Yes | Yes | Yes | Yes | Yes | Yes |
| Deyou Tan 2009^13^ | Yes | Yes | Yes | Yes | Yes | Yes | Yes | Yes | Yes |
| Fang Fan 2023^14^ | Yes | Yes | No | Yes | Yes | Yes | Yes | Yes | Yes |
| Fangkuan Hu 2014^15^ | Yes | Yes | No | Yes | Yes | Yes | Yes | Yes | Yes |
| Fuguang Li 2011^16^ | Yes | Yes | No | No | No | Yes | Yes | Yes | Yes |
| Guanghui Chen 2018^17^ | Yes | Yes | Yes | Yes | Yes | Yes | Yes | Yes | Yes |
| Guiqian Zhang 2015^18^ | Yes | Yes | Yes | Yes | No | Yes | Yes | Yes | Yes |
| Guohui Deng 2016^19^ | Yes | Yes | No | Yes | Yes | Yes | Yes | Yes | Yes |
| Haiming Ning 2015^20^ | Yes | Yes | Yes | Yes | Yes | Yes | Yes | Yes | Yes |
| Sijia Hao 2020^21^ | Yes | Yes | Yes | Yes | Yes | Yes | Yes | Yes | Yes |
| Wen Hu 2023^22^ | Yes | Yes | Yes | Yes | Yes | Yes | Yes | Yes | Yes |
| Huiqiang Liang 2013^23^ | Yes | Yes | No | Yes | Yes | Yes | Yes | Yes | Yes |
| Jianbin Wang 2018^24^ | Yes | Yes | No | Yes | Yes | Yes | Yes | Yes | Yes |
| Jianlei Wang2023^25^ | Yes | Yes | Yes | Yes | Yes | Yes | Yes | Yes | Yes |
| Jiarong Han 2016^26^ | Yes | Yes | Yes | Yes | Yes | Yes | Yes | Yes | Yes |
| Jie Pu 2013^27^ | Yes | Yes | Yes | Yes | Yes | Yes | Yes | Yes | Yes |
| Jifeng Liu 2011^28^ | Yes | Yes | No | Yes | Yes | Yes | Yes | Yes | Yes |
| Jingjing Xu2018^29^ | Yes | Yes | Yes | Yes | Yes | Yes | Yes | Yes | Yes |
| Jinhao Zhang 2013^30^ | Yes | Yes | No | Yes | Yes | Yes | Yes | Yes | Yes |
| Juan Tan 2012^31^ | Yes | Yes | Yes | Yes | Yes | Yes | Yes | Yes | Yes |
| Juan Wang 2020^32^ | Yes | Yes | Yes | Yes | Yes | Yes | Yes | Yes | Yes |
| Jun Zhang 2017^33^ | Yes | Yes | Yes | Yes | Yes | Yes | Yes | Yes | Yes |
| Kedi Wang 2016^34^ | Yes | Yes | Yes | Yes | Yes | Yes | Yes | Yes | Yes |
| Lanying Li 2017^35^ | Yes | Yes | Yes | Yes | No | Yes | Yes | Yes | Yes |
| Lanying Li 2012^36^ | Yes | Yes | Yes | Yes | No | Yes | Yes | Yes | Yes |
| Ayan-Jun Lei 2009^37^ | Yes | Yes | No | Yes | Yes | Yes | Yes | Yes | Yes |
| Lihong Pei 2013^38^ | Yes | Yes | Yes | Yes | Yes | Yes | Yes | Yes | Yes |
| Lingbo Jiang 2018^39^ | Yes | Yes | Yes | Yes | Yes | Yes | Yes | Yes | Yes |
| Liya Feng 2019^40^ | Yes | Yes | Yes | Yes | Yes | Yes | Yes | Yes | Yes |
| Qun Wang 1999^41^ | Yes | Yes | No | No | No | Yes | Yes | Yes | Yes |
| D.-M. Ma 2019^42^ | Yes | Yes | Yes | Yes | Yes | Yes | Yes | Yes | Yes |
| Min Xu 2017^43^ | Yes | Yes | Yes | Yes | Yes | Yes | Yes | Yes | Yes |
| Min Zhao 2013^44^ | Yes | Yes | Yes | Yes | No | Yes | Yes | Yes | Yes |
| Mingyang Liu 1998^45^ | Yes | Yes | No | Yes | Yes | Yes | Yes | Yes | Yes |
| Minzhao 2010^46^ | Yes | Yes | Yes | Yes | Yes | Yes | Yes | Yes | Yes |
| Ning Jin 2007^47^ | Yes | Yes | No | No | No | Yes | Yes | Yes | Yes |
| Ning Zhang 2018b^48^ | Yes | Yes | Yes | Yes | Yes | Yes | Yes | Yes | Yes |
| Ninghong Song^49^ | Yes | Yes | No | No | No | Yes | Yes | Yes | Yes |
| Qiang Ding 1996^50^ | Yes | Yes | Yes | Yes | Yes | Yes | Yes | Yes | Yes |
| Qiang Li 2020^51^ | Yes | Yes | Yes | Yes | Yes | Yes | Yes | Yes | Yes |
| Qihong Liao 2004^52^ | Yes | Yes | Yes | Yes | Yes | Yes | Yes | Yes | Yes |
| Qin Zhang 2018a^53^ | Yes | Yes | Yes | Yes | Yes | Yes | Yes | Yes | Yes |
| Qiulan Lin 2013^54^ | Yes | Yes | No | Yes | Yes | Yes | Yes | Yes | Yes |
| Shang Cheng 2022^55^ | Yes | Yes | Yes | Yes | Yes | Yes | Yes | Yes | Yes |
| Shang Cheng2023^56^ | Yes | Yes | Yes | Yes | Yes | Yes | Yes | Yes | Yes |
| Shaofang Wu 2014^57^ | Yes | Yes | Yes | Yes | Yes | Yes | Yes | Yes | Yes |
| Shenghua Li 2015^58^ | Yes | Yes | Yes | Yes | Yes | Yes | Yes | Yes | Yes |
| Shunming Xu 2012^59^ | Yes | Yes | Yes | Yes | Yes | Yes | Yes | Yes | Yes |
| Xu Tang 2006^60^ | Yes | Yes | No | Yes | Yes | Yes | Yes | Yes | Yes |
| Ting Yang 2010^61^ | Yes | Yes | Yes | Yes | Yes | Yes | Yes | Yes | Yes |
| Tsen-Fang Tsai 2008^62^ | Yes | Yes | Yes | Yes | Yes | Yes | Yes | Yes | Yes |
| Weiyuan Ma 2009^63^ | Yes | Yes | Yes | Yes | No | Yes | Yes | Yes | Yes |
| Wenge Wang 2013^64^ | Yes | Yes | No | Yes | Yes | Yes | Yes | Yes | Yes |
| Wenhai Li 2016^65^ | Yes | Yes | Yes | Yes | Yes | Yes | Yes | Yes | Yes |
| Wenhao Zhuang 2010^66^ | Yes | Yes | Yes | Yes | Yes | Yes | Yes | Yes | Yes |
| Wenhui Liu 2014^67^ | Yes | Yes | No | Yes | Yes | Yes | Yes | Yes | Yes |
| Xiangnian Shan 1993^68^ | Yes | Yes | No | Yes | No | Yes | Yes | Yes | Yes |
| Xiangyu Ma 2021^69^ | Yes | Yes | Yes | Yes | Yes | Yes | Yes | Yes | Yes |
| Xiaohui Zhang 2015b^70^ | Yes | Yes | Yes | Yes | Yes | Yes | Yes | Yes | Yes |
| Xiaojing Wu 2005^71^ | Yes | Yes | Yes | Yes | No | Yes | Yes | Yes | Yes |
| Xiaolei Wang 2014^72^ | Yes | Yes | No | Yes | Yes | Yes | Yes | Yes | Yes |
| Xiaoping Hu 2012^73^ | Yes | Yes | Yes | Yes | Yes | Yes | Yes | Yes | Yes |
| Xiaoxia Shen 2013a^74^ | Yes | Yes | No | Yes | Yes | Yes | Yes | Yes | Yes |
| Xiaoxia Shen 2013b^75^ | Yes | Yes | No | Yes | Yes | Yes | Yes | Yes | Yes |
| Xiaoxia Zhao 2017^76^ | Yes | Yes | Yes | Yes | Yes | Yes | Yes | Yes | Yes |
| Xinxin Tian 2016^77^ | Yes | Yes | Yes | Yes | Yes | Yes | Yes | Yes | Yes |
| Xiurong Long 2013^78^ | Yes | Yes | No | Yes | Yes | Yes | Yes | Yes | Yes |
| Xuejiao Yu 2017^79^ | Yes | Yes | Yes | Yes | Yes | Yes | Yes | Yes | Yes |
| Xuequn Yao 2015^80^ | Yes | Yes | No | Yes | Yes | Yes | Yes | Yes | Yes |
| Yan Lu 2006^81^ | Yes | Yes | No | Yes | Yes | Yes | Yes | Yes | Yes |
| Yanbing Yao2021^82^ | Yes | Yes | No | Yes | No | Yes | Yes | Yes | Yes |
| Yanghui Liu2017^83^ | Yes | Yes | No | Yes | No | Yes | Yes | Yes | Yes |
| Yangqing Zhong 2018^84^ | Yes | Yes | Yes | Yes | Yes | Yes | Yes | Yes | Yes |
| Yaohua Xue 2011^85^ | Yes | Yes | Yes | Yes | Yes | Yes | Yes | Yes | Yes |
| Yi Yang 2019^86^ | Yes | Yes | No | Yes | Yes | Yes | Yes | Yes | Yes |
| Ying Sun 2008^87^ | Yes | Yes | No | Yes | No | Yes | Yes | Yes | Yes |
| Ying Xiong 2007^88^ | Yes | Yes | Yes | Yes | No | Yes | Yes | Yes | Yes |
| Yong Qing 2019^89^ | Yes | Yes | No | Yes | No | Yes | Yes | Yes | Yes |
| Yong Tang2009^90^ | Yes | Yes | Yes | Yes | Yes | Yes | Yes | Yes | Yes |
| Yonggang Dai 2019^91^ | Yes | Yes | Yes | Yes | Yes | Yes | Yes | Yes | Yes |
| Youbao Wang 2010^92^ | Yes | Yes | No | Yes | Yes | Yes | Yes | Yes | Yes |
| Haowen Yuan 2023^93^ | Yes | Yes | Yes | Yes | Yes | Yes | Yes | Yes | Yes |
| Yuanyuan Cheng 2017^94^ | Yes | Yes | Yes | Yes | No | Yes | Yes | Yes | Yes |
| Yue Huang 2015^95^ | Yes | Yes | No | Yes | Yes | Yes | Yes | Yes | Yes |
| Yunfei He2015^96^ | Yes | Yes | No | Yes | Yes | Yes | Yes | Yes | Yes |
| Yun Gao 2019^97^ | Yes | Yes | Yes | Yes | Yes | Yes | Yes | Yes | Yes |
| Yuping Fu 2018^98^ | Yes | Yes | Yes | Yes | Yes | Yes | Yes | Yes | Yes |
| Yuping Fu 2020^99^ | Yes | Yes | No | Yes | Yes | Yes | Yes | Yes | Yes |
| Zeqi Huang 2014^100^ | Yes | Yes | Yes | Yes | Yes | Yes | Yes | Yes | Yes |
| Zeqi Huang 2018^101^ | Yes | Yes | Yes | Yes | Yes | Yes | Yes | Yes | Yes |
| Zeyu Chen 2016^102^ | Yes | Yes | No | Yes | Yes | Yes | Yes | Yes | Yes |
| Zhengrong Dong 2016^103^ | Yes | Yes | Yes | Yes | Yes | Yes | Yes | Yes | Yes |
| Zhenyu Bao 2016^104^ | Yes | Yes | No | Yes | Yes | Yes | Yes | Yes | Yes |
| Cansheng Zhu 2019^105^ | Yes | Yes | Yes | Yes | Yes | Yes | Yes | Yes | Yes |

Table S2 Quality assessment of included studies that provided data on patients with HPV-related cancer and precancer

| Study ID | *Was the sample representative of the target population?* | *Were study participants recruited in an appropriate way?* | *Was the sample size adequate?* | *Were the study subjects and setting described in detail?* | *Is the data analysis conducted with sufficient coverage of the identified sample?* | *Were objective, standard criteria used for measurement of the condition?* | *Was the condition measured reliably?* | *Was there appropriate statistical analysis?* | *Are all important confounding factors/ subgroups/differences identified and accounted for?* |
| --- | --- | --- | --- | --- | --- | --- | --- | --- | --- |
| Fa Chen 2016^106^ | Yes | Yes | No | Yes | Yes | Yes | Yes | Yes | Yes |
| Lianghai Wang 2016^107^ | Yes | Yes | No | Yes | Yes | Yes | Yes | Yes | Yes |
| Dong-Hong Zhang 2011a^108^ | Yes | Yes | No | Yes | Yes | Yes | Yes | Yes | Yes |
| Fangjia Tong 2018^109^ | Yes | Yes | No | Yes | Yes | Yes | Yes | Yes | Yes |
| B. Liu 2010a^110^ | Yes | Yes | No | Yes | Yes | Yes | Yes | Yes | Yes |
| De He 1997^111^ | Yes | Yes | No | Yes | Yes | Yes | Yes | Yes | Yes |
| Chengbiao Chu 2020^112^ | Yes | Yes | No | Yes | Yes | Yes | Yes | Yes | Yes |
| Ying-Tai Jin 1997^113^ | Yes | Yes | No | Yes | No | Yes | Yes | Yes | Yes |
| Chunyan Hu 2020^114^ | Yes | Yes | No | Yes | Yes | Yes | Yes | Yes | Yes |
| Fei He 2019^115^ | Yes | Yes | No | Yes | Yes | Yes | Yes | Yes | Yes |
| Li-Ang Lee 2015^116^ | Yes | Yes | Yes | Yes | Yes | Yes | Yes | Yes | Yes |
| Z.-y. Zhang 2004^117^ | Yes | Yes | No | Yes | Yes | Yes | Yes | Yes | Yes |
| C.-W. Luo 2007^118^ | Yes | Yes | No | Yes | Yes | Yes | Yes | Yes | Yes |
| Weijie Gu 2019^119^ | Yes | Yes | No | Yes | Yes | Yes | Yes | Yes | Yes |
| Kunsong Huang 2020^120^ | Yes | Yes | No | Yes | Yes | Yes | Yes | Yes | Yes |
| Xiangrui Ma 2017^121^ | Yes | Yes | No | Yes | Yes | Yes | Yes | Yes | No |
| Chih-Yen Chien 2007^122^ | Yes | Yes | Yes | Yes | Yes | Yes | Yes | Yes | Yes |
| Bin Lang 2023^123^ | Yes | Yes | No | Yes | Yes | Yes | Yes | Yes | Yes |
| Bei Chen 2004^124^ | Yes | Yes | No | Yes | Yes | Yes | Yes | Yes | Yes |
| Jun-Quan Yang 2019^125^ | Yes | Yes | Yes | Yes | Yes | Yes | Yes | Yes | Yes |
| Hong-xue Meng 2020^126^ | Yes | Yes | Yes | Yes | Yes | Yes | Yes | Yes | Yes |
| Lei Lei 1996^127^ | Yes | Yes | No | No | No | Yes | Yes | Yes | Yes |
| Xiao-Jie Chen 2016^128^ | Yes | Yes | Yes | Yes | Yes | Yes | Yes | Yes | Yes |
| Jianpo Zhai 2013^129^ | Yes | Yes | No | Yes | Yes | Yes | Yes | Yes | Yes |
| S-F Huang 2012^130^ | Yes | Yes | No | Yes | Yes | Yes | Yes | Yes | Yes |
| Lin Huang 2016^131^ | Yes | Yes | No | Yes | Yes | Yes | Yes | Yes | Yes |
| Timan Pa 2000^132^ | Yes | Yes | No | Yes | Yes | Yes | Yes | Yes | Yes |
| Lixiao Chen 2021^133^ | Yes | Yes | No | Yes | Yes | Yes | Yes | Yes | Yes |
| Wei Wei 2012^134^ | Yes | Yes | No | Yes | Yes | Yes | Yes | Yes | Yes |
| X. Chen 2017a^135^ | Yes | Yes | Yes | Yes | Yes | Yes | Yes | Yes | Yes |
| Wuhao Lu 2013^136^ | Yes | Yes | No | Yes | Yes | Yes | Yes | Yes | Yes |
| Na Zhao 2010^137^ | Yes | Yes | No | Yes | Yes | Yes | Yes | Yes | Yes |
| Liming Gao 2020^138^ | Yes | Yes | Yes | Yes | Yes | Yes | Yes | Yes | Yes |
| Xiaoling Min 2017^139^ | Yes | Yes | No | Yes | Yes | Yes | Yes | Yes | Yes |
| Cheng-Chih Huang 2011^140^ | Yes | Yes | No | Yes | No | Yes | Yes | Yes | Yes |
| Zhen Wang 2016^141^ | Yes | Yes | Yes | Yes | Yes | Yes | Yes | Yes | Yes |
| Dan Zhao 2009^142^ | Yes | Yes | No | Yes | Yes | Yes | Yes | Yes | Yes |
| Hui Huang 2012^143^ | Yes | Yes | No | Yes | Yes | Yes | Yes | Yes | Yes |
| Yanan Xu 2014^144^ | Yes | Yes | Yes | Yes | Yes | Yes | Yes | Yes | Yes |
| K W Chan 1994^145^ | Yes | Yes | No | Yes | No | Yes | Yes | Yes | Yes |
| Shuying Li 2018^146^ | Yes | Yes | No | Yes | Yes | Yes | Yes | Yes | Yes |
| Eddy WH Lam 2015^147^ | Yes | Yes | No | Yes | Yes | Yes | Yes | Yes | Yes |
| Tao Liu 2013^148^ | Yes | Yes | No | Yes | Yes | Yes | Yes | Yes | Yes |
| Longjia Qu 2010^149^ | Yes | Yes | No | Yes | Yes | Yes | Yes | Yes | Yes |
| Qiang Ding 1996^150^ | Yes | Yes | No | Yes | Yes | Yes | Yes | Yes | Yes |
| Yanan Xu 2017^151^ | Yes | Yes | No | Yes | Yes | Yes | Yes | Yes | Yes |
| Zhen Wang 2014^152^ | Yes | Yes | No | Yes | Yes | Yes | Yes | Yes | Yes |
| Yu Long 2011^153^ | Yes | Yes | No | Yes | Yes | Yes | Yes | Yes | Yes |
| Yongji Yan 2020^154^ | Yes | Yes | No | Yes | Yes | Yes | Yes | Yes | Yes |
| Yong Ping Wang 2008^155^ | Yes | Yes | No | Yes | Yes | Yes | Yes | Yes | Yes |
| J. M. Hu 2013^156^ | Yes | Yes | No | Yes | Yes | Yes | Yes | Yes | Yes |
| Qing Ying Zhang 2011b^157^ | Yes | Yes | No | Yes | Yes | Yes | Yes | Yes | Yes |
| Shuying Li 2017^158^ | Yes | Yes | No | Yes | Yes | Yes | Yes | Yes | Yes |
| Wei-Chih Chen 2017b^159^ | Yes | Yes | No | Yes | Yes | Yes | Yes | Yes | Yes |
| Xiaohong Fan 2014^160^ | Yes | Yes | No | Yes | Yes | Yes | Yes | Yes | Yes |
| Ya Wen Cheng 2004^161^ | Yes | Yes | No | Yes | Yes | Yes | Yes | Yes | Yes |
| Xiaobin Cui 2014^162^ | Yes | Yes | No | Yes | Yes | Yes | Yes | Yes | Yes |
| X.M. Lu 2008^163^ | Yes | Yes | No | Yes | Yes | Yes | Yes | Yes | Yes |
| K Shuyama 2007^164^ | Yes | Yes | No | Yes | Yes | Yes | Yes | Yes | Yes |
| Shu-Hsing Cheng 2017^165^ | Yes | Yes | No | Yes | No | Yes | Yes | Yes | Yes |
| Wei Li 2007^166^ | Yes | Yes | No | Yes | Yes | Yes | Yes | Yes | Yes |
| King-Yin Lam 1997^167^ | Yes | Yes | No | Yes | Yes | Yes | Yes | Yes | Yes |
| Jamil N. Al-Swiahb 2010^168^ | Yes | Yes | Yes | Yes | Yes | Yes | Yes | Yes | Yes |
| Hong-xue Meng 2018^169^ | Yes | Yes | Yes | Yes | Yes | Yes | Yes | Yes | Yes |
| Lifang Cui 2019^170^ | Yes | Yes | Yes | Yes | Yes | Yes | Yes | Yes | Yes |
| Enqi Wu 2019^171^ | Yes | Yes | No | Yes | Yes | Yes | Yes | Yes | Yes |
| Shu-Hsing Cheng 2014^172^ | Yes | Yes | Yes | Yes | No | Yes | Yes | Yes | Yes |
| Zhen-Qiang Sun 2013^173^ | Yes | Yes | No | Yes | Yes | Yes | Yes | Yes | Yes |
| Kuan-Ting Kuo 2008^174^ | Yes | Yes | No | Yes | Yes | Yes | Yes | Yes | Yes |
| Wen-Kang Liu 2010b^175^ | Yes | Yes | No | Yes | Yes | Yes | Yes | Yes | Yes |
| Jason Y.K. Chan 2022^176^ | Yes | Yes | No | Yes | Yes | Yes | Yes | Yes | Yes |
| Tian-You Ma 2007^177^ | Yes | Yes | No | Yes | Yes | Yes | Yes | Yes | Yes |
| Hongzhi Wang 2015^178^ | Yes | Yes | Yes | Yes | Yes | Yes | Yes | Yes | Yes |
| Shih-Wei Yang 2009^179^ | Yes | Yes | No | Yes | Yes | Yes | Yes | Yes | Yes |
| Guoying Ni 2019^180^ | Yes | Yes | No | Yes | Yes | Yes | Yes | Yes | Yes |
| PKS Chan 2017^181^ | Yes | Yes | Yes | Yes | Yes | Yes | Yes | Yes | Yes |
| Jinn-Li Wang 2014^182^ | Yes | Yes | No | Yes | Yes | Yes | Yes | Yes | Yes |
| Li-Ang Lee 2013^183^ | Yes | Yes | Yes | Yes | Yes | Yes | Yes | Yes | Yes |
| Yu-Feng Wang 2013^184^ | Yes | Yes | No | Yes | Yes | Yes | Yes | Yes | Yes |
| Julia Yu-Fong Chang 2003^185^ | Yes | Yes | No | Yes | Yes | Yes | Yes | Yes | Yes |
| Hui-Ling Chiou 2003^186^ | Yes | Yes | No | Yes | Yes | Yes | Yes | Yes | Yes |
| Ming Zhang 2009^187^ | Yes | Yes | No | Yes | Yes | Yes | Yes | Yes | Yes |
| Eddy WH Lam, FRCSEd2018^188^ | Yes | Yes | Yes | Yes | Yes | Yes | Yes | Yes | Yes |
| Haizhong Zhang 2016^189^ | Yes | Yes | No | Yes | Yes | Yes | Yes | Yes | Yes |
| Yuanlin Liu 1995^190^ | Yes | Yes | No | Yes | No | Yes | Yes | Yes | Yes |
| Ling Liu 2014^191^ | Yes | Yes | No | Yes | Yes | Yes | Yes | Yes | Yes |
| Xiaogang Du 2010^192^ | Yes | Yes | No | Yes | Yes | Yes | Yes | Yes | Yes |
| Yihong Zhuang 2005^193^ | Yes | Yes | No | Yes | Yes | Yes | Yes | Yes | Yes |
| Zhen Wang 2015^194^ | Yes | Yes | Yes | Yes | Yes | Yes | Yes | Yes | Yes |
| LiFang Cui 2021^195^ | Yes | Yes | Yes | Yes | Yes | Yes | Yes | Yes | Yes |
| Meiling Mao 2018^196^ | Yes | Yes | Yes | Yes | Yes | Yes | Yes | Yes | Yes |
| Lifang Cui2018^197^ | Yes | Yes | No | Yes | Yes | Yes | Yes | Yes | Yes |
| Heng‑Jui Chang 2023^198^ | Yes | Yes | Yes | Yes | Yes | Yes | Yes | Yes | Yes |
| Chun-Yi Chuang 2012^199^ | Yes | Yes | No | Yes | Yes | Yes | Yes | Yes | Yes |
| Shu-Hsing Cheng 2015^200^ | Yes | Yes | Yes | Yes | No | Yes | Yes | Yes | Yes |
| Yan Yu 2015^201^ | Yes | Yes | No | Yes | Yes | Yes | Yes | Yes | Yes |
| Ming-Fang Wu 2005^202^ | Yes | Yes | No | Yes | Yes | Yes | Yes | Yes | Yes |
| Dakai Zhang 2017^203^ | Yes | Yes | No | Yes | Yes | Yes | Yes | Yes | Yes |
| Wen-Lun WANG 2015^204^ | Yes | Yes | No | Yes | Yes | Yes | Yes | Yes | Yes |
| Gian Luca Armas 2008^205^ | Yes | Yes | No | Yes | Yes | Yes | Yes | Yes | Yes |
| Wan-Yu Lo 2007^206^ | Yes | Yes | No | Yes | Yes | Yes | Yes | Yes | Yes |
| Tsen-Fang Tsai 2008^62^ | Yes | Yes | Yes | Yes | Yes | Yes | Yes | Yes | Yes |

**Supplementary 4 Characteristics of included studies**

Table S3 Characteristics of included studies with patients with genital warts

| **Study ID** | **Study design** | **Region** | **Population** | **Sample size tested for HPV** | **Recruitment time** | **Age, years**  Median (range)/mean±standard deviation | **HPV detection method** | **Genotypes determined by PCR** | **Anatomical site (eg.Anal canal and perianal regions)** |
| --- | --- | --- | --- | --- | --- | --- | --- | --- | --- |
| Aifen Liang 2017^1^ | Cross-sectional study | Guangdong | Condyloma acuminatum | 156 | 2014.06-2016.06 | NR | PCR | NR | Exfoliated cell specimens from warts. |
| Aiqin Yang 2018^2^ | Cohort study | Henan | Condyloma acuminatum | 165 | 2015.01-2017.02 | NR | PCR | 18 high - risk types (16,18,31,33,35,39,45,51,52,53,56,58,59,66,68,73,82,83); 5 low-risk types :(6,11,42,43,81) | Specimens were collected from the urethral orifice, glans, coronary sulcus, frenulum, prepuce, penis, and perianal warts |
| Bin Shen 2007^3^ | Cross-sectional study | Zhejiang | Condyloma acuminatum | 137 | 2005.1-2006.6 | NR | Fluorescence quantitative polymerase chain reaction (FQ-PCR) | NR | Swab specimens were obtained from the wart site. |
| Bingxiong Fang 2018^4^ | Cross-sectional study | Guangdong | Condyloma acuminatum | 551 | 2016.01-2017.12 | 31.7±10.4 | PCR | 15 kinds of high-risk type (16,18,31,33,35,39,45,51,52,53,56,58,59,66,68); 6 low-risk types :(6,11,42,43,44,CP8304) | Warts specimen, exfoliated cells of urethra, other pathological specimens. |
| Lihong Chang 2013^5^ | Cross-sectional study | Seven geographic regions (northeast, north, northwest, central, south- west, south, and east) in China | genital warts | 449 | 2007.09 - 2008.04 | 34.0 ± 11.4 | PCR | low-risk HPV types HPV6, 11, 7, 61, 81; and high-risk HPV types: HPV 16, 58, 59, 45, 66, 67. | biopsy specimens |
| Dian Chen 2011^6^ | Cross-sectional study | Guangdong | MSM with condyloma acuminatum were infected with HIV | 138 | 2011.02 - 2011.04 | Range: 20-73 | NR | NR | Perianal and oral specimens: 1. Perianal specimens: brush the sample on the perianal area for several times, and oral specimens: brush the sample on the lateral wall of the oropharynx for several times. |
| Xiaogang Chen 2016^7^ | Cross-sectional study | Shanghai | Genital warts | 935 | 2014.01 - 2015.05 | NR | PCR | 15 high-risk HPV strains (HPV16, 18, 31, 33, 35, 39, 45, 51, 52, 53, 56, 58, 59, 66 and 68) and 6 low-risk HPV strains (HPV6, 11, 42, 43, 44 and CP8304) | Biopsies of the outer genital area (scrotum or penis) |
| Chenghu Wang 2010^8^ | Cross-sectional study | Anhui | Condyloma acuminatum | 138 | 2003.3-2005.6 | NR | PCR | HPV 6, 11, 16, 18 | Patients with confirmed condyloma acuminatum: warts tissue; Subclinical patients with condyloma acuminatum: secretions and swab specimens from lesions. |
| Chenglong Zeng 2020^9^ | Cross-sectional study | Shaanxi | Condyloma acuminatum | 5717 | 2010.09 - 2019.9 | Range: 9-84 | PCR | 18 high-risk types(HPV16, 18, 31, 33, 35, 39, 45, 51, 53, 56, 58, 59, 66, 68,73,82,83) and 5 low-risk types(HPV6, 11,42,43, 81) | The warts were fully exposed, and one of them was randomly selected. First, the warts were cleaned with normal saline, then the lesions were disinfected, and the warts were removed with sterile biopsy forceps and placed in specimen preservation solution. |
| Chunxiong Zhao 2009^10^ | Case-control study | Shandong | Condyloma acuminatum | 104 | NR | NR | PCR | HPV6, 11 | Cryopreserved specimens of skin lesions. |
|  |  |  |  | 86 |  |  |  |  | Frozen pubic hair specimens. |
| Xianling Cong 2016^11^ | Cross-sectional study | Jilin | Condyloma acuminatum (CA) | 50 | 2001.11 - 2003.10 | NR | PCR | HPV type 6, 11, 16, 18, 31, 33, and 58 | Genital lesions consistent with exophytic CA were removed by electrocautery after local anesthesia from the 80 patients and stored in ethanol and formalin. |
| Y. Dai 2008^12^ | Cross-sectional study | Guangdong | condyloma accuminatum (CA) | 43 | 2004 - 2006 | NR | PCR | 5 low-risk subtypes (HPV 6, 8, 11, 44, and 53), 14 high-risk subtypes (16, 18, 31, 33, 35, 39, 45, 51, 52, 56, 58, 59, 66, and 68), and 1 unknown-risk subtype CP8304 | CA tissues and cast-off samples were taken from genitals. |
| Deyou Tan 2009^13^ | Case-control study | Guangdong | Condyloma acuminatum | 106 | 2008.3-2008.12 | NR | PCR+ nucleic acid molecular rapid flow-through hybridization gene chip | 5 Low-risk ：HPV6, ll, 42, 43, 44;  13high-risk types:HPVl6, 18, 31, 33, 35, 39, 45, 5l, 52, 56, 58, 59, 68； 3 Intermediate-risk HPV53, 66 and CP8304 | Frozen tissue biopsy specimens of genital warts were obtained. |
| Fang Fan 2023^14^ | Case-control study | Hubei | Condyloma acuminatum | 119 | 2020.01-2021.01 | NR | PCR | NR | After local disinfection and anesthesia of the warts, the male genitalia, anus and surrounding surfaces were scraped with HPV sampling brushes for many times, and the exfoliated cells were removed and placed into corresponding sampling tubes and stored in a 4℃ refrigerator for later use. |
| Fangkuan Hu 2014^15^ | Cross-sectional study | Henan | Condyloma acuminatum | 154 | 2010.1-2012.12 | NR | PCR+ gene chip hybridization | 23 types of HPV | Paraffin-embedded tissue specimens of the anus and anal canal. |
| Fuguang Li 2011^16^ | Cross-sectional study | NR | Condyloma acuminatum | 156 | NR | NR | PCR | HPV 16,18,33,42,52,56,58 and 6,11 | CA tissues or secretions |
| Guanghui Chen 2018^17^ | Cohort study | Henan | Condyloma acuminatum | 332 | 2015.03-2017.09 | Range: 1-70  31.5± 10.5 | PCR | 21 high - risk,18,26,31,33,35,39,45,51,52,53,56,58,59,66,67,68,69,73,82,83 types; 16 kinds of low-risk type: (6,11,34,40,42,43,44,54,55,57,61,70,71,72,84,81 (CP8304) | Exfoliated cells were obtained by wiping the surface of the wart tissue several times with a sterile cotton swab. |
| Guiqian Zhang 2015^18^ | Cross-sectional study | Yunnan | Condyloma acuminatum | 46 | NR | NR | PCR | HPV 6,11,16,18,31,33,35,39,42,43,44,45,51,52,53,56,59,66,68 CP8304 21 types of HPV | The tissue and secretion of condyloma acuminatum were scraped from the surface of genital and perianal lesions and placed in a sampling tube with HPV-specific cell preservation solution, stored in a refrigerator at 4℃, and the test was completed within two weeks. |
| Guohui Deng 2016^19^ | Case-control study | Guangdong | Condyloma acuminatum | 25 | NR | NR | PCR | HPV-23 | Skin lesions shed cells. |
| Haiming Ning 2015^20^ | Cross-sectional study | Zhejiang | Condyloma acuminatum | 110 | 2012.12-2013.11 | Range: 16-65 | Pcr-reverse dot blot hybridization | "The 16 high-risk subtypes of HPV16, 18, 31, 33, 35, 39, 45, 51, 52, 53, 56, 58, 59, 66, 68 and CP8304(a high-risk subtype unique to Chinese); Low-risk subtype HPV6, 11, 19(43) | Wart tissue (samples were collected from the coronal sulcus of prepuce, meatus and perianal). |
| Sijia Hao 2020^21^ | Cross-sectional study | Jilin | Condyloma acuminatum | 634 | 2017.10-2019.09 | NR | PCR | HPV 16, 18, 31, 33, 35, 39, 45, 51, 52, 56, 58, 59, 66 and 68；HPV 53；HPV 6, 11, 42, 43, 44, and 81 | And epithelial cells from common warts and genital warts were collected with a cytobrush by epithelial brushing. |
| Wen Hu 2023^22^ | Cross-sectional study | Xinjiang | Condyloma acuminatum | 499 | 2016.12-2018.12 | NR | PCR | NR（23 types) (HybriBio). | To sample the shed cells on the surface of the wart, the wart was punctured with a disposable syringe, and the lesion was then repeatedly wiped with a cotton swab to obtain sufficient shed cells For the collection of male urethral specimens, the urethra was penetrated about 2–4cm with a fine cotton swab that was then gently twisted to collect epithelial cells. |
| Huiqiang Liang 2013^23^ | Cross-sectional study | Guangdong | Condyloma acuminatum | 41 | 2011.06-2012.06 | NR | PCR | NR | The warts were exposed, and the skin lesion tissue of about 2mnl×2ram×2mm in size was taken by tissue clamp and placed in a sterile test tube towel of lml saline, one 20. C Save to be tested. |
| Jianbin Wang 2018^24^ | Cross-sectional study | Guangdong | Condyloma acuminatum | 57 | 2014.02-2015.12 | NR | PCR | NR | After the patient was instructed to avoid washing hands for more than 4 hours, epithelial cells were removed by brushing the patient's fingertips 10 times with a cell brush. |
| Jianlei Wang2023^25^ | Case-control study | Beijing | Condyloma acuminatum | 167 | 2018.01-2020.01 | NR | PCR | 10 low-risk types(HPV6, 11, 40, 42, 43, 44, 54, 61, 81, 83) and 18 high-risk types(HPV6, 11, 40, 42, 43, 44, 54, 61, 81, 83) HPV16, 18, 26, 31, 33, 35, 39, 45, 51, 52, 53, 56, 58, 59, 66, 68, 73, 82) | Cotton swabs were used to remove excessive secretions from the surface of CA warts (glans, coronal sulcus, foreskin, penis, scrotum and other areas with obvious skin lesions), and exfoliated epithelial cells samples were collected from the skin lesions using a special exfoliated cell collector, and the samples were labeled. The samples were stored at 2 ℃ ~ 8 ℃ and detected within 7 days. |
| Jiarong Han 2016^26^ | Cohort study | Ningxia | Condyloma acuminatum | 52 | 2013.12-2015.12 | NR | PCR amplification, flow fluorescence hybridization typing detection method | 27 types of HPV DNA: 16 high - risk types (16,18,31,33,35,39,45,51,52,53,56,58,59,66,68,82); 8 low-risk types :(6,11,40,42,43,44,61,81) | Exfoliated cells or wart tissue on the surface of warts |
| Jie Pu 2013^27^ | Cross-sectional study | Jiangsu | Condyloma acuminatum | 409 | 2010-2012 | 35.63 ± 12.55 | PCR+ gene chip hybridization | 18 high-risk types (HPV-16, 18, 31, 33, 35, 39, 45, 51, 53, 56, 58,59,66,68,73,83, MM4), 5 low-risk types (HPV-6, 11, 42, 43, 44) | Tissue samples of condyloma acuminatum. |
| Jifeng Liu 2011^28^ | Case-control study | Zhejiang | Condyloma acuminatum | 101 | 2008.1-2009.12 | NR | PCR+ gene chip hybridization | NR | Mung bean-sized wart tissue was removed surgically or harvested with sterile forceps. |
| Jingjing Xu2018^29^ | Cross-sectional study | Zhejiang | Condyloma acuminatum | 80 | 2014.01-2017.12 | NR | PCR | NR | The tissue were removed with special surgical scissors. The samples were stored in a centrifuge tube with cell preservation solution. Prostatic fluid (0.05 ml) was obtained by transrectal prostatic massage. Before sampling, the first drop of prostatic fluid was removed and the prostatic fluid was stored in cell preservation solution. |
| Jinhao Zhang 2013^30^ | Cross-sectional study | Jiangsu | Anal and anal canal condyloma acuminatum | 126 | 1985.01-2012.05 | NR | PCR | 23 HPV types (6, 11, 42-45, 16, 18, 31, 33, 35, 39, 51-53, 56, 58, 59, 66, 68, 73, 83, MM4 type) | Paraffin tissue sections of the anus and anal canal with condyloma acuminatum surgically removed. |
| Juan Tan 2012^31^ | Cohort study | Shanghai | Condyloma acuminatum | 200 | 2009.05-2011.07 | Range: 18-77  40.0±14.2 | PCR | 17 high - risk types: (16,18,31,33,35,45,51,52,53,56,58,59,66,68,73, type the CP8304); 9 low-risk types :(6,7,11,40,42,43,44,54,67) | Skin lesions of about 2mm×2mm×2mm were sheared |
| Juan Wang 2020^32^ | Cross-sectional study | Shanghai | Condyloma acuminatum | 2202 | 2017.03 - 2018.03 | Range: 13-89  34 ± 12 | PCR | 17 high - risk types: HPV 16,18,31,33,35,39,45,51,52,53,56,58,59,66,68, 73; 6 low-risk types 6,11,42,43, 81,83 | Exfoliated cells were collected from lesions of condyloma acuminatum in male patients by sterile cotton swabs. |
| Jun Zhang 2017^33^ | Cohort study | Jiangxi | Condyloma acuminatum | 128 | 2014.01-2016.06 | Range:18-80  34.59± 14.17 | PCR | 18 High Risk-HPV types (16, 18, 31,33, 35, 39,45, 51, 52, 56,58, 59,68,73,82,53,66,CP8304)；10 Low risk （HPV 6,11,40,42,43,44,54,61,81,83) | Epithelial tissue was scraped from the warts. |
| Kedi Wang 2016^34^ | Cohort study | Beijing | Condyloma acuminatum | 1074 | 2015.01 - 2015.08 | Range: 17-65  Mean: 36 | PCR | HR- HPV 16, 18, 31, 33, 35, 39, 45, 51, 52, 53, 56, 58, 59, 66, 68. LR-HPV 6, 11, 42, 43, 44, CP8304 (81) | The warts or skin lesions were scraped slightly vigorously with a male swab and placed in a tube containing a dedicated cell preservation solution. |
| Lanying Li 2017^35^ | Cohort study | Shanghai | Condyloma acuminatum | 132 | 2012.06-2015.06 | Range：17-57  28.99±9.90 | PCR | 16 High Risk-HPV types (16, 18, 31,33, 35, 39,45, 51, 52, 53,56,58, 59,66,68,CP8304)；5 Low risk （HPV 6,11,42,43,44) | Some verrucous tissue |
| Lanying Li 2012^36^ | Cohort study | Shanghai | Perianal anal canal condyloma acuminatum | 57 | 2008.10-2010.06 | Range：16-65  Mean: 32.5 | PCR | 16 High Risk-HPV types (16, 18, 31,33, 35, 39,45, 51, 52, 53,56,58, 59,66,68,CP8304)；5 Low risk （HPV 6,11,42,43,44,81) | Some verrucous tissue |
| Ayan-Jun Lei 2009^37^ | Cross-sectional study | Beijing | Condyloma acuminatum | 38 | 2006.04-2006.12 | NR | PCR | HPV 1, 2, 4, 7, 27, 57 and 65 | Liquid nitrogen-frozen specimens |
| Lihong Pei 2013^38^ | Cross-sectional study | Zhejiang | Condyloma acuminatum | 389 | 2010.5.1 -2012.5.1 | Range: 23- 66 | PCR+ reverse dot blot hybridization of DNA | HPV(6, 11, 16, 18, 31, 33, 35, 39, 42, 43, 44, 45, 51, 52, 53, 56, 58, 59, 66, 68, CP8304) | Skin lesions of condyloma acuminatum. |
| Lingbo Jiang 2018^39^ | Cross-sectional study | Shandong | Condyloma acuminatum | 2830 | 2012.01-2016.08 | NR | PCR | 17 kinds of high-risk type (16,18,31,33,35,39,45,51,52,53,56,58,59,66,68,73,82); 6 low-risk types :(6,11,42,43,81,83) | Swabs or tissues from the skin lesions of condyloma acuminatum in the foreskin, coronary groove, urethral orifice, or perianal area of male patients. |
| Liya Feng 2019^40^ | Cross-sectional study | Zhejiang | Condyloma acuminatum | 4500 | 2014.1-2016.12 | NR | PCR+ gene chip reverse dot blot hybridization | High-risk types-16, 18, 31, 33, 35, 39, 45, 51, 52, 53, 56, 58, 59, 66, 68, 73, 82 and 83 Low-risk subtypes -6, 11, 42, 43, and 81 | Secretions and tissue specimens from male prepuce, coronary sulcus, meatus, perianal, etc. |
| Qun Wang 1999^41^ | Cross-sectional study | NR | Condyloma acuminatum | 32 (29 urethral condyloma acuminatum, 3 penile condyloma acuminatum) | NR | NR | PCR | NR | Urine and tissue samples (31 from patients with urethral condyloma acuminatum and 3 from penile condyloma acuminatum) were fixed in 10% formalin and embedded in paraffin." |
| D.-M. Ma 2019^42^ | Cross-sectional study | Shandong | Condyloma acuminatum | 426 | 2016.01-2018.12 | NR | PCR | 15 HR-HPV(16, 18, 31, 33, 35, 39, 45, 51, 52, 56, 58, 59, 66, 68, and 82) and 2 LR-HPVs (6 and 11) | Genital specimens were obtained from the coronal sulcus, glans penis, penile shaft, scrotum, and urethral using dacron swabs, which were subsequently combined into one sample |
| Min Xu 2017^43^ | Case-control study | Tianjin | Condyloma acuminatum | 158 | 2015.9-2016.12 | NR | PCR + membrane hybridization method | 21 types of HPV | NR |
| Min Zhao 2013^44^ | Case-control study | Hubei | Condyloma acuminatum | 166 | NR | NR | PCR+ gene chip hybridization | 5 low-risk subtypes (6, 11, 42, 43, 44) 18 high-risk types (16, 18, 31, 33, 35, 39, 45, 51, 52, 53, 56, 58, 59, 66, 68, 73, 83, MM4) | Exfoliated cells were found in glans, perianal and anal canal. |
| Mingyang Liu 1998^45^ | Cohort study | Shandong | Condyloma acuminatum | 250 | 1995.07-1998.07 | NR | PCR | NR | Tissue of warts. |
| Minzhao 2010^46^ | Cross-sectional study | Hubei | Condyloma acuminatum | 157 | 2005.02-2008.01 | NR | PCR | 18 kinds of high-risk type: (16,18,31,33,35,39,45,51,52,53,56,58,59,66,68,73,83, MM). 5 low-risk types :(6,11,42,43,44) | Ca-like tissue specimens from patients |
| Ning Jin 2007^47^ | Cross-sectional study | Liaoning | Condyloma acuminatum | 52 | 2005.07-2006.06 | NR | PCR | HPV 6,11,16,18 | If warts were visible, warts were exposed and routinely disinfected, and about 0.5m³ warts were collected with an ophthalmic sterile small camera. |
| Ning Zhang 2018^48^ | Cross-sectional study | Tianjin | Condyloma acuminatum | 623 | 2014.01-2017.12 | 32 (26-42) | PCR | 13 kinds of high-risk type: (16,18,31,33,35,39,45,51,52,56,58,59,68); 2 low-risk types :(6,11) | The warts were exposed and washed many times with normal saline. After local anesthesia, the warts were destroyed with sharp instruments, and then the appropriate amount of warts tissue debris was soaked in a moist cleaning cotton swab. |
| Ninghong Song^49^ | Case-control study | NR | Condyloma acuminatum | 52 | NR | NR | PCR | NR | Urine samples, paraffin-embedded tissues. |
| Qiang Ding 1996^50^ | Cross-sectional study | Shanghai | Condyloma acuminatum | 34 | 1984-1993 | Mean(range):44(20-67). | PCR | HPV 6, 11 | Specimens were obtained from DNA paraffin-embedded tissue from condyloma acuminatum tissues in the coronary sulcus, frenulum, and foreskin. |
| Qiang Li 2020^51^ | Cross-sectional study | Shandong | Condyloma acuminatum | 70 | 2017.01 - 2018.12 | range: 18 – 66  Mean: 33 | PCR | 17 high-risk types: HPV16, 18, 31, 33, 35, 39, 45, 51, 52, 53, 56, 58, 59, 66, 68, 73, 82; 6 low-risk genotypes: HPV6, 11, 42, 43, 81, 83 | A special sterile brush was used to place the swab on the surface of the wart and slowly rub it back and forth for 3-5 times to obtain sufficient epithelial cells. |
| Qihong Liao 2004^52^ | Cross-sectional study | Guangxi | Condyloma acuminatum | 260 | NR | Mean(range):29(18-65). | PCR | NR | The urethral secretions, prostatic fluid or wart tissue were taken immediately for examination. |
| Qin Zhang 2018^53^ | Cross-sectional study | Chongqing | Condyloma acuminatum | 224 | 2016.09-2017.05 | NR | PCR | 24 subtypes, including 18 high-risk and 6 low-risk subtypes | Specimens were obtained from prepuce, urethral meatus, labia large and small, vaginal meatus, cervix, perianal and intraanal skin lesions. |
| Qiulan Lin 2013^54^ | Cross-sectional study | Beijing | Condyloma acuminatum | 112 | 2011. 11 -2013.4 | NR | PCR+ gene chip hybridization | 18 high-risk types:(HPV16, 18, 31, 33, 35, 39, 45, 51, 53, 53, 58, 59, 66, 68, 73, 83 and MM4) and 5 low-risk genotypes (HPV6, 11,42,43 and 44)" | The wart tissue shed cells. |
| Shang Cheng 2022^55^ | Cohort study | Shandong | Condyloma acuminatum | 159 | 2021.01-2022.01 | 33 (15-75) | PCR | 17 kinds of high-risk type (16,18,26,31,33,35,39,45,51,52,53,56,58,59,66,68,82); 10 kinds of low-risk type：(6,11,40,42,43,44,55,61,81,83) | Male sterile swab was used to wipe the warts and skin lesions several times until enough exfoliated cell secretion was obtained. |
| Shang Cheng2023^56^ | Case-control study | Shandong | Condyloma acuminatum | 159 | 2021.01-2022.01 | Range: 15-75 | PCR | 10 low-risk type including 6,11,40,42,43,44,55,61,81,83; 17 high - risk types: 16,18,26,31,33,35,39,45,51,52,53,56,58,59,66,68,82 | The male sterile swab was used to wipe the warts and skin lesions of the patient for several times, and the exfoliated cell secretion was taken into a sterile test tube and immediately sent to the central laboratory of our hospital for detection |
| Shaofang Wu 2014^57^ | Cross-sectional study | Zhejiang | Condyloma acuminatum | 111 | 2011.05-2013.04 | NR | PCR | 16 kinds of high-risk type: (16,18,31,33,35,39,45,51,52,53,56,58,59,66,68 CP8304); 5 low-risk types :(6,11,42,43,44) | Biopsy forceps were used to collect CA wart tissue about 0.3cm ×0.3cm ×0.3cm |
| Shenghua Li 2015^58^ | Cross-sectional study | Zhejiang | Condyloma acuminatum | 201 | 2013.4-2014.3 | 34. 39 ± 12. 11 | PCR+ HPV genotyping detection kit | 23 HPV genotypes, including 18 high-risk types and 5 low-risk types | The wart tissue shed cells. |
| Shunming Xu 2012^59^ | Cross-sectional study | Shanghai | Condyloma acuminatum | 187 | 2009.05-2011.07 | 38.2 ± 12. 32 | PCR | HPV 6，7，11，40，42，43，44，54，67 ；HPV 16，18，31，33，35，39，45，51，52，53，56，58，59，66，68，73，subtype CP8304 | A 2mm×2mm×2mm lesion was obtained from each patient and placed in a sterilized test tube containing normal saline. The tissue was then wrapped in aluminum foil and numbered in a liquid nitrogen tank (-196 ° C) and frozen for storage. |
| Xu Tang 2006^60^ | Cross-sectional study | Zhejiang | Condyloma acuminatum | 30 | 2003.09-2004.04 | NR | PCR | HPV 16, 18, 31, 33；HPV 6, 11, 42,54 | For each subject, a urethral swab was inserted 2 cm into the urethral meatus and rotated 360o. After being inserted into a centrifugal tube containing 2 mL of normal saline solution, each swab was squeezed and rinsed for 1 min to make DNA dissolve sufficiently in the solvent |
| Ting Yang 2010^61^ | Cross-sectional study | Jiangsu | Condyloma acuminatum | 100 | 2008.11-2009.11 | range: 16-73  Mean: 37.5 | PCR | 18 high-risk types (HPV-16, 18, 31, 33, 35, 39, 45, 51, 25, 53, 56, 58, 59, 66, 68, 73, 83, MM4) and 5 low-risk types (HPV-6, 11, 42, 43, 44). | Genital condyloma acuminatum tissue samples were collected |
| Tsen-Fang Tsai 2008^62^ | Cross-sectional study | Taiwan | condyloma | 36 | 1991-2005 | NR  Mean: 36 | PCR+Gene Chip HPV Genotyping | 39 types of HPV DNA (6, 11, 16, 18, 26, 31, 32, 33, 35, 37, 39, 42, 43, 44, 45, 51,52, 53, 54, 55, 56, 58, 59, 61, 62, 66, 67, 68, 69, 70, 72, 74, 82, CP8061, CP8304, L1AE5, MM4, MM7, and MM8) | Formalin-fixed and paraffin-embedded (FFPE) surgical specimens |
| Weiyuan Ma 2009^63^ | Cross-sectional study | Shandong | Condyloma acuminatum | 107 | 2006.12-2008.2 | 23.6（17-47） | PCR | HPV6/11, 16, 18 | Swab specimens of epithelial cells from skin lesions at the site of warts. |
| Wenge Wang 2013^64^ | Cross-sectional study | Beijing | Condyloma acuminatum | 18 | 2011.6-2011. 12 | NR | PCR+ membrane hybridization method | 21 types of HPV | The wart tissue shed cells. |
| Wenhai Li 2016^65^ | Cross-sectional study | Beijing | Condyloma acuminatum | 68 | 2011.10-2015.06 | Mean(range):33.6(16-62). | DNA microarray technology combining PCR and reverse dot blot hybridization | HPV16,18,31,33,35,39,45,51,52,53,56,58,59,66,68,73,83,MM4, and HPV 6,11,42,43,44 | Samples were obtained from urethral orifice warts. Methods: The warts were exposed, and the brush provided by the kit was used to wipe the surface of the warts slightly hard several times to obtain exfoliated cells, which were placed in a sample tube containing normal saline, immediately sent to the laboratory and stored at -20℃ until detection. |
| Wenhao Zhuang 2010^66^ | Cross-sectional study | Fujian | Condyloma acuminatum | 125 | 2009.01-2009.12 | NR | PCR | HPV 6、11 ;HPV 16、18、31、33、45、52、56、58 | Skin lesions were obtained by repeatedly wiping the condyloma acuminatum tissue with cotton swabs. |
| Wenhui Liu 2014^67^ | Case-control study | Shandong | Condyloma acuminatum | 184 | 2009.01-2011.01 | NR | PCR | HPV 16,18,31,33,35,39,45,51,52,53,56,58,59,66,68,73,83,MM4;HPV 6,11,42,43,44 | After routine disinfection of the vulva, the large light bodies of rice grains were retained with scissors and placed in different microcentrifuge tubes, and frozen at -20℃. |
| Xiangnian Shan 1993^68^ | Cross-sectional study | Jiangsu | Condyloma acuminatum | 12 | NR | NR | PCR | 1 high-risk type :(16); 2 low-risk types :(6,11) | Male prepuce, glans, meatus, penile shaft, coronal sulcus, frenulum, and perianal biopsy specimens. |
| Xiangyu Ma 2021^69^ | Cohort study | Shandong | Condyloma acuminatum | 425 | 2019.01-2020.12 | NR | PCR | 17 kinds of high - risk types (16,18,31,33,35,39,45,51,52,53,56,58,59,66,68,72,82); 6 low-risk types :(6,11,42,43,81,83) | Clinical specimens were collected using swabs from the penis, coronal sulcus, etc |
| Xiaohui Zhang 2015^70^ | Cross-sectional study | Guangdong | Anorectal condyloma acuminatum | 55 | 2012- 2014 | 31 ± 9 | Pcr-reverse dot blot assay | 19 high-risk types HPV(16, 18, 26, 3l, 33, 35, 39, 45, 51, 52, 53, 56, 58, 59, 66, 68, 73, 82, 83) 9 Low-risk HPV(6, 11, 40, 42, 43, 44, 54, 61, 81) | Epidermal cells of anorectal warts. |
| Xiaojing Wu 2005^71^ | Cross-sectional study | Beijing+Hebei | Condyloma acuminatum | 13 | NR | NR | PCR | NR | Tissue mass of genital condyloma acuminatum. |
| Xiaolei Wang 2014^72^ | Cross-sectional study | Tianjin | Condyloma acuminatum | 50 | 2012.06-2012.09 | NR | PCR | 2 high-risk types :(16,18); 2 low-risk types :(6,11) | The warts were exposed and a small amount of part of the wart tissue was taken with tissue scissors. |
| Xiaoping Hu 2012^73^ | Cross-sectional study | Guangdong（Shenzhen） | Condyloma acuminatum | 741 | 2010.03 - 2012.03 | range: 16-72  31.2±8.2 | PCR | 18 high-risk types (HPV16, 18, 31, 33, 35, 39, 45, 51, 52, 53, 56, 58, 59, 66, 68, 73, 83, MM4) and 5 low-risk types (HPV6, 11, 42, 43, 44) | Cotton swabs were used to remove excessive secretions on the surface of skin lesions, and exfoliated epithelial cells samples were collected from skin lesions using a special exfoliated cell collector, and samples were labeled. |
| Xiaoxia Shen2013a^74^ | Case-control study | Shanxi (Xian) | Condyloma acuminatum | 43 | 2010.12-2012.09 | 31.23±13.54 | PCR | HPV 6, 11 | The exfoliated cells were obtained by rubbing with a cotton swab soaked in normal saline for 3 to 6 times, and then placed into a test tube containing 0.5mL of sterile normal saline. Urethral mucosa sampling: A cotton swab was gently extended into the urethral orifice about 2cm, rotated 360°, and paused for a moment to remove exfoliated epithelial cells, which were placed into a test tube equipped with 0.5mL sterile saline. |
| Xiaoxia Shen2013b^75^ | Case-control study | Shanxi (Xian) | Condyloma acuminatum | 46 | 2010.12-2012.09 | 35.46 ± 12.06 | PCR | HPV 6, 11 | A sterile male calcium alginate cotton swab soaked in normal saline was wiped slightly and repeatedly for 3 to 6 times, and the exfoliated cells were placed into a test tube containing 0.5mL of sterile normal saline. The pulp of the dominant index finger of the hand was moistened with normal saline, and then gently scraped with a disposable scalpel for 3 to 6 times. Then the exfoliated cells were washed repeatedly in a test tube containing 0.5 mL sterile normal saline. |
| Xiaoxia Zhao 2017^76^ | Case-control study | Hainan | Condyloma acuminatum | 443 | 2014.6-2016.6 | NR | PCR+ nucleic acid molecular rapid flow-through hybridization gene chip | 37 types of HPV | The genital warts were removed by tissue scissors. |
| Xinxin Tian 2016^77^ | Cross-sectional study | Shandong | Condyloma acuminatum | 1225 | 2011.12-2014.11 | NR | PCR | 18 kinds of high-risk type (16,18,31,33,35,39,45,51,52,53,56,58,59,66,68,73,82,83); 5 low-risk types :(6,11,42,43,81) | Swabs or tissues from the skin lesions of male patients with condyloma acuminatum. |
| Xiurong Long 2013^78^ | Cross-sectional study | Jiangsu | Condyloma acuminatum | 176 | 2010.01-2012.04 | Range: 20-61  Mean: 32.1 | PCR | NR | Specimens of urethral meatus epithelial cells |
| Xuejiao Yu 2017^79^ | Cohort study | Zhejiang | Condyloma acuminatum | 566 | 2014.08-2017.08 | Range: 16-73  Mean: 44.5 | PCR | 21 types of HPV: 13 high - risk types (16,18,31,33,39,45,52,53,56,58,59,66,68); 6 low-risk types :(6,11,42,43,44,CP8304) | Skin lesion specimens from CA patients |
| Xuequn Yao 2015^80^ | Cross-sectional study | Hunan | Condyloma acuminatum | 120 | 2013.1-2014.12 | 35.15（16-61） | PCR+ reverse dot blot hybridization of DNA | 18 high-risk types: 16, 18, 31, 33, 35, 39, 45, 51, 52, 53, 56, 58, 59, 66, 68, 73, 82, 83; 5 low-risk types: 6, 11, 42, 43 and 81. | Swab specimens were obtained from the wart site. |
| Yan Lu 2006^81^ | Case-control study | Jiangsu | Condyloma acuminatum | 72 | 2004.6 - 2006.4 | Range：17-76  34.14 ± 10.18 | PCR | NR | According to the clinical manifestations, acetowhite test and pathological biopsy if necessary. |
| Yanbing Yao2021^82^ | Cohort study | Fujian | Condyloma acuminatum | 69 | 2019.01-2020.01 | NR | PCR | 18 high - risk types (16,18,31,33,35,39,45,51,52,53,56,58,59,66,68,73,82,83); 4 low-risk types :(6,11,42,43) | NR |
| Yanghui Liu2017^83^ | Cross-sectional study | Shandong | Anal anal canal condyloma acuminatum | 116 | 2014.9-2016.8 | Range: 18-52  29.41±6.86 | PCR | 18 high-risk types (HPV 16, 18, 31, 33, 35, 39, 45, 51, 52, 53, 56, 58, 59, 66, 68, 73, 82, and 83); 5 low-risk genotypes (HPV6, 11, 42, 43 and 81) | Anal canal swab |
| Yangqing Zhong 2018^84^ | Cross-sectional study | Guangdong | Condyloma acuminatum | 1147 | 2015.5-2017.5 | Range: 7-84  31.1±8.2 | PCR | HPV6, 11, 42, 43, 81, 16, 18, 31, 33, 35, 45, 51, 52, 56, 58, 61, 66, 68, 73, 82, 83 | Swabs or tissues from the skin lesions of condyloma acuminatum were taken from the male foreskin, coronary groove, urethral orifice, perianal, etc. |
| Yaohua Xue 2011^85^ | Cross-sectional study | Guangdong | Condyloma acuminatum | 81 | 2008.5-2009.12 | NR | PCR+ gene chip flow-through reverse dot blot hybridization | 6 low-risk types 6, 11, 42, 43, 44 and CP 8304; 15 high-risk types: including 16, 18, 31, 33, 35, 39, 45, 51, 52, 53, 56, 58, 59, 66 and 68. | Tissue swab specimens of condyloma tissue lesions on genital and adjacent surfaces. |
| Yi Yang 2019^86^ | Cross-sectional study | Sichuan | Condyloma acuminatum | 242 | 2016.7-2017.9 | NR | PCR | High-risk types (16, 18, 31, 33, 35, 39, 45, 51, 52, 53, 56, 58, 59, 66, 68 types) Low-risk (6, 11, 42, 43, 44, CP8304) | Tissue fluid and exfoliated cells from skin lesions of condyloma acuminatum. |
| Ying Sun 2008^87^ | Cross-sectional study | Shanghai | Condyloma acuminatum | 44 | 2006.10-2006.12 | 35.6 ± 10.4 | PCR | HPV 6 ,11 ,16 ,18 | Exfoliated cells on the surface of warts were collected. Exfoliated cells were obtained by rubbing the wart surface back and forth several times with a cotton swab infiltrated with normal saline. |
| Ying Xiong 2007^88^ | Cross-sectional study | Shandong | Condyloma acuminatum | 58 | 2005.09-2006.09 | NR | PCR | HPV 6 ,11 ,16 ,18 | After cleaning the affected area with 0.9% normal saline, the affected area was repeatedly wiped with a sterile cotton swab slightly and vigorously for about 10 times before being sent for examination |
| Yong Qing 2019^89^ | Cohort study | Sichuan | Condyloma acuminatum complicated with HIV infection | 64 | 2015.07-2017.06 | 31 (16-85) | PCR | NR | NR |
| Yong Tang2009^90^ | Cross-sectional study | Hunan | Condyloma acuminatum | 60 | 2006.07-2008.08 | NR | PCR | HPV 6／11、16／18 | The exposed warts of some CA patients were routinely disinfected, and about 0.5m³ warts were collected with an ophthalmic sterile mini-camera. For male, a sterile calcium alginate cotton swab was gently inserted into the urethra for 2-3 cm, rotated 360° and stayed for 2s. Then, the swab was placed in 0.5 mL normal saline for examination. |
| Yonggang Dai 2019^91^ | Cross-sectional study | Shandong | Condyloma acuminatum | 269 | 2014.1-2017.7 | Range: 17- 65 | PCR+ reverse dot blot hybridization of DNA | 5 Low risk : HPV6, 11, 43, 42, 81; 18 High risk :HPV52, 16, 18, 51, 59, 58, 68, 53, 66, 33, 56, 35, 31, 39, 45, 73, 83, 82 | Tissue biopsies or swabs of genital and perianal skin lesions were obtained. |
| Youbao Wang 2010^92^ | Cross-sectional study | Shandong | Condyloma acuminatum | 104 | NR | NR | PCR | NR | Frozen tissue of condyloma acuminatum. |
|  |  |  |  | 86 |  |  |  |  | Pubic hair cryopreserved specimens. |
| Haowen Yuan 2023^93^ | Cross-sectional study | Shandong | Condyloma acuminatum | 597 | 2019.08-2021.06 | NR | PCR | HPV 16, 18, 31, 33, 35, 39, 45, 51, 52, 53, 56, 58, 59, 66, 68, 73, and 82；HPV 6, 11, 42, 43, 81, and 83 | Exfoliated cell specimen was collected from the surface of each CA lesion using a sampling brush. The brush was rotated 3–5 full turns in clockwise direction to ensure acquisition of the exfoliated cells. For each patient, each specimen was independently eluted from the sampling brush with normal saline and stored in the refrigerator at −80°C before detecting HPV genotypes |
| Yuanyuan Cheng 2017^94^ | Cross-sectional study | Shanghai | Condyloma acuminatum | 162 | 2013.10-2017.01 | 31 (18-59) | PCR | 27 HPV subtypes (including HPV6, 11, 16, 18, 26, 31, 33, 35, 39, 40, 42, 43, 44, 45, 51, 53, 53, 56, 58, 59, 61, 66, 68, 81, 82, 83) | (1) Exfoliated cells from anal condyloma acuminatum were collected with a collection brush and placed into the elution tube containing cell preservation solution for HPV detection and typing analysis. (2) Exfoliated cells in the oral cavity were collected by the same method as in the anus, and the collection site was the oral side wall. If there was oral condyloma acuminatum, the wart body was collected. |
| Yue Huang 2015^95^ | Cohort study | Beijing | Condyloma acuminatum | 43 | 2012.8-2014.3 | NR | PCR | 13 high - risk types (16,18,31,33,35,39,45,51,52,56,58,59,68); 2 low-risk types :(6,11) | A sampling brush was used to collect the external orifice of the urethra of the subject for central sampling |
| Yunfei He2015^96^ | Cross-sectional study | Sichuan | Condyloma acuminatum | 30 | 2011.09-2013.09 | Range: 19-71  32.5±4.9 | PCR | HPV 6 /11、16 /18 | The wart tissue was collected. In male patients, a sterile calcium alginate cotton swab was gently extended into the urethra for 2 to 3cm and rotated 360° |
| Yun Gao 2019^97^ | Cross-sectional study | Hubei | Condyloma acuminatum | 73 | 2014.01-2016.06 | NR | PCR | 18 kinds of high-risk type (16,18,31,33,35,39,45,51,52,53,56,58,59,66,68,73,82,83); 5 low-risk types :(6,11,42,43,81) | Specimens of CA skin lesions |
| Yuping Fu 2018^98^ | Cross-sectional study | Liaoning | Condyloma acuminatum | 98 | 2016.1-2017. 1 | NR | Pcr-reverse dot blot assay | 18 high-risk types: 16, 18, 31, 33, 35, 39, 45, 51, 52, 53, 56, 58, 59, 66, 68, 73, 83, 82; 5 low-risk types: 6, 11, 42, 43, 81 | Approximately 2 cubic mm of large skin lesion tissue was biopsied. |
| Yuping Fu 2020^99^ | Cohort study | Liaoning | Condyloma acuminatum | 155 | 2017.06-2019.12 | Range: 17-68  34.3 ± 11.0 | PCR | 18 high-risk types:16、18、31、33、35、39、45、51、52、53、56、58、59、66、68、73、83、82）；5 low-risk types:(6、11、42、43、81） | A cotton swab was gently extended into the urethral orifice about 2 cm, rotated 360°, and stayed for a moment to remove mucosal exfoliated cells. |
| Zeqi Huang 2014^100^ | Cross-sectional study | Guangdong | Condyloma acuminatum | 223 | 2011.05-2013.10 | 32 (16-78) | PCR | NR | Condyloma tissue was scraped from male genitalia and adjacent surfaces. |
| Zeqi Huang 2018^101^ | Cross-sectional study | Guangdong | Condyloma acuminatum | 500 | 2012.7-2016.9 | 32（9～83） | PCR+ gene chip hybridization | HPV low-risk types 6, 11, 42, 43, 44, Chinese CP8304; high-risk types:16, 18, 31, 33, 35, 39, 45, 51, 52, 56, 58, 59, 68, China 53, China 66. | Condyloma tissue on the genitals and adjacent surfaces. |
| Zeyu Chen 2016^102^ | Cross-sectional study | Xinjiang | Condyloma acuminatum | 94 | 2014.6-2015.6 | NR | PCR | NR | Frozen specimens of perianal warts were collected. |
| Zhengrong Dong 2016^103^ | Case-control study | Guangdong | Condyloma acuminatum | There were 113 patients with condyloma acuminatum and 56 patients with subclinical condyloma acuminatum | 2014.01-2015.12 | Range: 18-65 | PCR | HPV 6, 11, 16, 18, 31, 33, 35, 39, 42, 43, 44, 45, 51, 52, 53, 56, 58, 59, 66, 68 and CP8304 | A sterile cotton swab, moistened with normal saline, was wiped over the candidate site to collect exfoliated cells. Specifically: (1) Condyloma acuminatum, the positive sites were collected if the acetowhite test was positive, and the skin lesions were directly collected if the test was negative. (2) subclinical condyloma acuminatum, collecting acetowhite test positive sites; |
| Zhenyu Bao 2016^104^ | Cross-sectional study | Beijing | Condyloma acuminatum | 76 | 2015.10-2016.01 | NR | PCR | 15 kinds of high-risk type (16,18,31,33,35,39,51,52,53,56,58,59,66,68,82); 5 low-risk types :(6,11,40,43,61) | The warts were exposed, washed with normal saline first, and then the special brush provided by the kit was used to rub the wart head repeatedly for several times to obtain exfoliated cells. |
| Cansheng Zhu 2019^105^ | Cross-sectional study | Shanxi | Condyloma acuminatum | 512 | 2014.9-2017.4 | NR | automatic nucleic acid hybridisation system | low-risk HPV types, HPV6, 11, 42, 43 and 81,   high-risk HPV types, HPV16, 18, 31, 33, 35, 39, 45, 51, 52, 53, 56, 58, 59, 66, 68, 73, 82 and 85 | lesions of genital warts |

Table S4 Characteristics of included studies with patients with HPV-related cancer and precancer

| **Study ID** | **Study design** | **Region** | **Population** | **Sample size tested for HPV** | **Recruitment time** | **Age, years**  Median (range)/mean±standard deviation | **HPV detection method** | **Genotypes determined by PCR** | **Anatomical site (eg.Anal canal and perianal regions)** |
| --- | --- | --- | --- | --- | --- | --- | --- | --- | --- |
| Fa Chen 2016^106^ | Case-control study | Fujian | Oral squamous cell carcinoma (OSCC) | 178 | 2012.09 - 2015.09 | Median (IQR): 58.92 (45.92–69.18) | PCR+Flow-through Hybridization and Gene chip | 15 high-risk HPV genotypes (HPV 16, 18, 31, 33, 35, 39, 45, 51, 52, 53, 56,58, 59, 66, 68), and 6 low-risk HPV genotypes (HPV 6, 11, 42, 43, 44,CP8304) | Tumor tissues from all OSCC patients after surgical resection, and then were immediately frozen at -80℃until use |
| Lianghai Wang 2016^107^ | Case-control study | Xinjiang | Esophageal squamous cell carcinoma (ESCC) | 158 | 1984 - 2013 | Mean:  <58: 85 ≥58: 67 | PCR | NR | Formalin-fixed paraffin-embedded tissue (FFPE) blocks from patients who underwent esophagectomy without prior chemotherapy or radiotherapy were obtained |
| Dong-Hong Zhang 2011a^108^ | Case-control study | Guangdong | Esophageal carcinoma (EC) | 70 | 2000 - 2006 | Range: 40-78  57.3±1.08 | PCR | HPV 16 | Cancerous tissues and paracancerous normal tissues (dissected from an area ≥7 cm away from the tumor lesions) |
| Fangjia Tong 2018^109^ | Cross-sectional study | Heilongjiang | Laryngeal squamous cell carcinoma (LSCC) | 211 | NR | Mean (range): 58.2 (37-80) | PCR | HPV 16,18,31,33,35,39,45,51,52,58,59,68, and 70 | Tumor specimens were collected during surgery, and one representative paraffin block was selected for each patient. |
| B. Liu 2010^110^ | Cross-sectional study | Beijing | Laryngeal squamous cell carcinoma (LSCC) | 84 | 2000 - 2008 | 64 (38-74) | PCR | HPV 16,18 | Paraffin-embedded tissue samples of primary laryngeal carcinoma |
| De He 1997^111^ | Cross-sectional study | Sichuan | Esophageal squamous cell carcinoma (ESCC) | 152 | 1987 - 1994 | age: no. of participant 30-40: 9 41-50: 32 51-60: 54 >60: 48 | PCR | HPV 6,11,16,18,31,32 | 97 paraffin-embedded specimens and 55 freshly frozen specimens |
| Chengbiao Chu 2020^112^ | Cohort study | Guangdong | Penile cancer | 226 | 1999 - 2013 | 52 (24-86) | PCR | 13 HR-HPVs (subtype, 16, 18, 31, 33, 35, 39, 45, 51, 52, 56, 58, 59, and 68), 5 low-risk HPVs (LR-HPVs) (subtype, 6, 11, 42, 43, and 44), and other HPV types commonly found in Chinese populations (subtype, 53, 66, 73, 82 and 81/CP8304) | 186 primary tumors and 40 lymph node metastatic specimens |
| Ying-Tai Jin 1997^113^ | Cross-sectional study | Taiwan | Squamous cell carcinoma of the middle ear (MESCC) | 14 | NR | Mean (range): 57 (36-69) | PCR | HPV 6,11,16,18,31,33,52 | Biopsy of cancerous area; middle ear |
| Chunyan Hu 2020^114^ | Cross-sectional study | Shanghai | Sinonasal squamous cell carcinoma (SNSCC) | 114 | 2010 - 2017 | IP-SNSCC: 58 (45–82) DN-SNSCC: 59 (24–84) | PCR | 17 high-risk types (HPV 16, 18, 31, 33, 35, 39, 45, 51, 52, 56, 58, 59, 66, 68, 53,73, 82) and six low-risk types (HPV 6, 11, 42, 43, 44, 81) | Formalin-fixed and paraffin-embedded (FFPE) tissues were retrospectively collected from SNSCC patients who underwent surgical treatment at the Eye and ENT |
| Fei He 2019^115^ | Case-control study | Fujian | Primary lung cancer | 87 | 2013.11-2015.05；2014.05-2016.05 | NR | PCR+a diversion hybrid gene chip | 15 high-risk types (HPV16, 18, 31, 33, 35, 39, 45, 51, 52,53, 56, 58, 59, 66, 68) and six low risk types (HPV6, 11, 42,43, 44, CP8304 (81) | Pulmonary cancer tissues and selfmatched adjacent normal tissues (more than 5 cm away from the cancer tissues as autologous controls) were collected during surgery. |
| Li-Ang Lee 2015^116^ | Cohort study | Taiwan | oral cavity cancer (OCC) | 938 | 2004-2011 | NR | PCR | NR（39 distinct HPV genotypes） | Formalin-fixed, paraffin-embedded tumor samples collected during radical surgery |
| Z.-y. Zhang 2004^117^ | Case-control study | Shanghai | Oral squamous cell carcinoma (OSCC) | 48 | 1997-1999 | NR | PCR | HPV 16,18 | Samples were obtained from surgical tumour resections carried out in the Department of Oral and Maxillofacial Surgery |
| C.-W. Luo 2007^118^ | Case-control study | Taiwan | Oral squamous cell carcinoma (OSCC) | 48 | 2022.01-2004.05 | NR | PCR+ gene-chip arrays | 39 genotypes of HPV DNA (6, 11, 16, 18, 26, 31, 32, 33, 35, 37, 39, 42, 43, 44, 45, 51, 52, 53, 54, 55, 56, 58, 59, 61, 62, 66, 67, 68, 69, 70, 72, 74, 82, CP8061, CP8304, L1AE5, MM4, MM7 and MM8) | Cytological specimens were obtained from the lesions of each subject before surgery. The brush was held against the mucosa of the lesion and rotated 10 full turns. |
|  |  |  | precancerous lesions, including leukoplakia, oral submucous fibrosis and verrucous hyperplasia. | 45 |  |  |  |  |  |
| Weijie Gu 2019^119^ | Cross-sectional study | multicenter: Shanghai, Shandong, Hainan, Jiangsu | Penile cancer | 340 | 2006-2017 | Median (IQR): 56（47-65） | PCR | 18 high-risk HPV types (16, 18, 31, 33, 35, 39, 45, 51, 52, 53, 56, 58, 59, 66, 68, 73, 82, and 83) and 5 low-risk HPV types (6, 11, 42, 43, and 81). | Formalin-fixed, paraffin-embedded (FFPE) penile cancer specimens |
| Kunsong Huang 2020^120^ | Case-control study | Guangdong | Tongue squamous cell carcinoma(TSC) | 83 | 2011-2018 (Guangzhou); 2012-2017 (Foshan) | NR | PCR-RDB | 17 high-risk (HR) HPV types (16, 18, 31, 33, 35, 39, 45, 51, 52, 53, 56, 58, 59, 66, 68, 73, and 82) and 6 low-risk (LR) HPV types (6, 11, 42, 43, 81, and 83). | DNA was extracted from three pieces of 5-mm-thick formalin-fixed paraffin-embedded (FFPE) tumor tissue sections using TaKaRa MiniBEST FFPE DNA Extraction Kit (TaKaRa Bio Group,Shiga, Japan) |
| Xiangrui Ma 2017^121^ | Case-control study | Sichuan | Head and neck squamous cell carcinomas (HNSCC) | 1.103 fresh tumor tissues (male) 2.111 blood samples with available tumor DNA (male) | 2012-2015 | NR | PCR | HPV 16,18 | fresh primary tumor tissues and peripheral blood samples |
| Chih-Yen Chien 2007^122^ | Cross-sectional study | Taiwan | Squamous cell carcinoma of tonsil (TSCC) | 100 | 1992-2005 | NR | PCR | HPV 6, 11, 16,and 18 | Ten tissue sections, 10 lm thick, from the selected tissue block for each specimen were collected in a 1.5 ml Eppend off tube for DNA extraction. |
| Bin Lang 2023^123^ | Cross-sectional study | Hubei | Penile cancer | 103 | 2013.08-2019.08 | Range: 24-90  62.18 ± 13.35 | PCR (hybrid capture-based NGS assay) | 17 HPV genomes (HPV6, 11, 16, 18, 31, 33, 35, 39,45, 52, 56, 58, 59, 66, 68, 69, and 82). | Formalin-Fixed and Paraffin-Embedded (FFPE) samples of histologically confirmed cases of penile cancer |
| Bei Chen 2004^124^ | Cross-sectional study | Shandong | Squamous cell carcinoma of the head and neck | 51 | NR | NR | PCR | All types of HPV | Pre-treatment biopsy tissue or surgically resected tumor specimens were taken, and at least five sections 5μm thick were cut from each specimen. |
| Jun-Quan Yang 2019^125^ | Cross-sectional study | Hebei | Head and neck cancer | 261 | 2003.01-2013.12 | NR | PCR-reverse dot blot hybridization | NR | surgically-removed specimens of head and neck carcinoma |
| Hong-xue Meng 2020^126^ | Cohort study | Heilongjiang | Oropharyngeal squamous cell carcinoma (OPSCC) | 1197 | 2000.01-2017.01 | NR | PCR | NR | The acquired tissue was taken during the operation of the patient. |
| Lei Lei 1996^127^ | Case-control study | Hubei | Oral squamous cell carcinoma | 17 | NR | NR | PCR | HPV 16,18 | One part of the biopsy or surgical specimen was sent for pathological examination, and the other part was stored at -20℃ to prepare tissue DNA by the conventional proteinase K-phenol-chloroform extraction method. |
| Xiao-Jie Chen 2016^128^ | cross-sectional study | Shanghai | Oral squamous cell carcinoma (OSCC) and Oral potentially malignant disorders (OPMD) | 59 | NR | Range: 28-81 | real-time PCR | HPV 16, 18 | Paired tissue and serum samples were collected from each patient. Tissue samples were immediately frozen at −80 °C after surgery |
| Jianpo Zhai 2013^129^ | Cohort study | Beijing | Penile cancer | 28 | 2000 - 2009 | Mean (range): 60.5 (28, 89) | PCR | NR | The penis was partially or totally removed, and all specimens were fixed in 4% formaldehyde, routinely embedded in paraffin, stained with HE, and confirmed by pathology. One sample of wax scraps was cut from each case for DNA extraction by PCR. |
| S-F Huang 2012^130^ | cross-sectional study | Taiwan | oral cavity squamous cell carcinoma (OSCC) | 96 | 1997.03 - 2003.12 | NR | PCR | HPV 6, 11, 16, 18, 26, 31, 32, 33, 35, 37, 39, 42, 43, 44, 45, 51, 52, 53, 54, 55, 56, 58, 59, 61, 62, 66, 67, 68, 69, 70, 71 (CP8061), 72, 74, 81 (CP8304), 82 (MM4), 83 (MM7), 84 (MM8), and L1AE5 | Ten micrometer sections were cut from the paraffin blocks of cancer tissues and collected into 1.5-ml Eppendorf tubes for DNA extraction |
| Lin Huang 2016^131^ | Case-control study | Guangxi | Prostate Cancer (PCa) | 75 | 2012 - 2015 | Range: 55-88  72.1±6.4 | PCR + RDB | HPV 16, 18 | Prostate biopsy or surgical removal of PCa tissue. |
| Timan Pa 2000^132^ | Case-control study | Shanghai | Oral squamous cell carcinoma | 48 | 1997 - 1999 | NR | PCR | HPV 16, 18 | Surgical resection specimens |
| Lixiao Chen 2021^133^ | Cohort study | Shanghai | Squamous cell carcinoma of the larynx and hypopharynx (SCCLHP) | 110 | 2016.06 - 2019.07 | NR | PCR | NR | formalin-fixed paraffin-embedded tumor sample |
| Wei Wei 2012^134^ | cross-sectional study | Beijing | malignant squamous tumors, including laryngo- carcinoma, oropharyngeal carcinoma, hypopharynx carcinoma and lip carcinoma | 84 | 2006 - 2011 | NR | PCR | detect 26 genotypes of HPVs including 19 high-risk HPV types (HPV16, 18, 26, 31, 33, 35, 39, 45, 51, 52, 53, 55, 56, 58, 59, 66, 68, 82 and 83) and 7 low-risk HPV types (HPV6, 11, 40, 42, 44, 61 and 73). | All samples were surgically removed and conventionally fixed in 10% formalin and paraffin embedded. Tumor tissues were extracted from the paraffin-embedded tissue blocks with a commercial genomic DNA extraction FFPE kit (Qiagen) |
| X. Chen 2017a^135^ | Case-control study | Beijing | Laryngeal squamous cell carcinoma (LSCC) | 220 | 2013.01 - 2015.12 | NR | PCR | NR | Tissue specimens were collected from cases and controls at surgical resection |
| Wuhao Lu 2013^136^ | cross-sectional study | Henan | Pharyngeal squamous cell carcinoma (OSCC) | 26 | 2011.07 - 2012.06 | Range: 52-73 | PCR | Five low-risk and 18 high-risk HPV types (unspecified) | Part of the tumor tissue with typical morphology was cut immediately after the tumor was isolated |
| Na Zhao 2010^137^ | Case-control study | Zhejiang | Rectal cancer | 53 | 2007 - 2009 | NR | PCR | HR: HPV16, 18, 31, 33, 35, 39, 45, 51, 52, 56, 58, 59, 68  LR: HPV6, 11, 42, 43, 44  HPV53，66，CP8304 | Rectal cancer lesion tissue, adjacent mucosa tissue and peripheral blood samples from the scm of the lesion. |
| Liming Gao 2020^138^ | Case-control study | Beijing | Laryngeal squamous cell carcinoma | 220 | 2013.01 - 2015.12 | ≥18 | PCR | HR: HPV 16、18、31、33、39、40、45、51、52、56、58、59、66、68  LR: HPV 6、11、34、35、42、43、44、53、54、70、74 | Pathological specimens of laryngeal squamous cell carcinoma |
| Xiaoling Min 2017^139^ | Case-control study | Sichuan | Laryngeal squamous cell carcinoma, precancerous lesions (laryngeal papilloma, vocal cord leukoplakia) | 57 | 2012.01 - 2015.03 | NR | PCR | NR | Part of the lesion was sectioned in paraffin during surgery |
| Cheng-Chih Huang 2011^140^ | Case-control study | Taiwan | Nasopharyngeal carcinoma (NPC) | 36 | 2003 - 2004 | NR | PCR | HPV 6, 11, 16, 18, 26, 31, 32, 33, 35, 37, 39, 42, 43, 44, 45, 51, 52, 53, 54, 55, 56, 58, 59, 61, 62, 66, 67, 68, 69, 70, 72, 74, 82, CP8061, CP8304, L1AE5, MM4, MM7 and MM8 | primary NPC specimens |
| Zhen Wang 2016^141^ | cross-sectional study | Shanghai | Oropharyngeal squamous cell carcinoma (OPSCC) | 168 | 2008.01 - 2014.04 | NR | PCR | 18 high-risk types (HPV 16, 18, 31, 33, 35, 39, 45, 51, 52, 53, 56, 58, 59, 66, 68, 73, 82 and 83) and 5 low-risk types (HPV 6, 11, 42, 43 and 44). | Archived, formalin-fixed, and paraffin-embedded (FFPE) OPSCC specimens |
| Dan Zhao 2009^142^ | cross-sectional study | Hubei | Oral squamous cell carcinoma (OSCC). | 35 | 1999 - 2001 | NR | PCR | NR | The 10% formalin-fixed and paraffin-embedded tumor specimens |
| Hui Huang 2012^143^ | Cross-sectional study | Beijing | Oral squamous cell carcinoma (OSCC). | 61 | 1999.01 - 2009.12 | NR | PCR | NR | Formalin-fixed, paraffin-embedded tissue blocks |
| Yanan Xu 2014^144^ | Cohort study | Shanghai | Laryngeal squamous cell carcinoma (LSCC) | 654 | started 2006.04 | NR | PCR | 13 HR types (types 16, 18, 31, 33, 35, 39, 45, 51, 52, 56, 58, 59, and 68), 2 probable HR types (types 53 and 66), and 6 LR and unknown-risk types (types 6, 11, 42, 43, 44, and CP8304 [HPV-81]) | One representative formalin-fixed, paraffin-embedded (FFPE) block was retrieved for each case. |
| K W Chan 1994^145^ | Cross-sectional study | Hong Kong | Penile cancer | 41 | 1974-1992 | 59 (29-84) | PCR | HPV 16,18 | Paraffin embedded tissue sections |
| Shuying Li 2018^146^ | Cross-sectional study | Hebei | esophageal carcinoma (EC) | 136 | 2013.03-2015.12 | NR | PCR | HPV 16 | Fresh surgically resected tissue |
| Eddy WH Lam 2015^147^ | Cross-sectional study | Hong Kong | oropharyngeal squamous cell carcinoma | 178 | 2005-2009 | NR | PCR | NR | representative FFPE tumor tissue cell blocks |
| Tao Liu 2013^148^ | Cross-sectional study | Xinjiang | esophageal squamous cell carcinoma (ESCC) | 153 | 2005-2008 | NR | PCR | HPV 16,18 | The paraffin-embedded ESCC tissues |
| Longjia Qu 2010^149^ | Case-control study | Shaanxi | Transitional cell carcinoma of the bladder | 56 | 2008.9-2009.8 | 58.7±7.7 (38-72) | Gene hybridisation HC2 technology | HPV16、18、31、33、35、39、45、51、52、56、58、59、68 | Paraffin-fixed or frozen male bladder cancer tissues surgically removed or biopsied |
| Qiang Ding 1996^150^ | Cross-sectional study | Shanghai | Penile cancer | 28 | 1980-1993 | 38 (25-61) | PCR | HPV 16,18 | Paraffin-embedded specimen of penile cancer. |
| Yanan Xu 2017^151^ | Cross-sectional study | Shanxi | Squamous cell carcinoma of the skin | 201 | 2006.01-2016.01 | NR | PCR | 4 high-risk types :(16,18,31,33); 2 low-risk types :(6,11) | Tissue specimens from patients with cutaneous squamous cell carcinoma |
| Zhen Wang 2014^152^ | Cross-sectional study | Hubei | Squamous cell carcinoma of the skin | 196 | 2001.01-2011.01 | NR | PCR | 4 high-risk types :(16,18,31,33); 2 low-risk types :(6,11) | Tissue specimen of cutaneous squamous cell carcinoma |
| Yu Long 2011^153^ | Cross-sectional study | Sichuan | Head and neck squamous cell carcinoma | 59 | 2009.05-2010.10 | NR | PCR | 7 high-risk types :(16,18,33,45,52,58,59) | Lesion tissue specimens and oropharyngeal swab specimens were obtained. |
| Yongji Yan 2020^154^ | Cross-sectional study | Beijing | Bladder cancer | 122 | 2015-2019 | 66.42± 0.95 | PCR | HPV6, 11, 16, 18, 31, 33, 35, 39, 45, 52, 56, 58, 59, 66, 68, 69, 82 | DNA samples analyzed in this study were extracted from frozen tumor tissues which collected from the patients |
| Yongping Wang 2008^155^ | Cross-sectional study | Hubei | Non-small cell lung carcinoma(NSCLC) | 237 | NR | NR | PCR | HPV 16,18 | Lung tissue resected from primary tumors provided fresh samples, which were immediately frozen at -80˚C. |
| J. M. Hu 2013^156^ | Case-control study | Xinjiang(Kazakh) | Esophageal squamous cell carcinoma (ESCC) | 93 | 2000-2006 | NR | PCR | HPV 16 | All ESCC specimens were obtained from the surgery and embedded in paraffin, subsequently sectioned into 5 mm slices and subjected to conventional hematoxyllin & eosin staining |
| Qing Ying Zhang 2011b^157^ | Case-control study | Guangdong | Esophageal carcinoma (EC) | 71 | 2000-2006 | NR | PCR | HPV 16,18 | Every fresh specimen was divided into 2 parts: cancerous and paracancerous normal tissues (dissected from an area ≥7 cm away from the tumor lesions) |
| Shuying Li 2017^158^ | Cross-sectional study | Hebei | Esophageal carcinoma (EC) | 136 | 2013.03-2015.12 | NR | PCR | HPV16 | Fresh surgically resected tissue samples |
| Wei-Chih Chen 2017b^159^ | Cohort study | Taiwan | laryngeal squamous cell carcinoma (LSCC) | 103 | 2006 - 2009 | NR | PCR | 39 different HPV types (not report the specific type) | Paraffin-embedded samples from identified tumor blocks of each specimen |
| Xiaohong Fan 2014^160^ | Cross-sectional study | Shanghai | non-small-cell lung cancer (NSCLC)-squamous cell carcinoma (SQC) | 70 | 2004 - 2010 | NR | PCR | NR | Genomic DNA was extracted and isolated from tissue sections using conventional methods |
| Ya Wen Cheng 2004^161^ | Case-control study | Taiwan | primary lung cancer | 96 | NR | NR | nesteed PCR | HPV 6 /11 | Genomic DNA was prepared from a tissue section |
| Xiaobin Cui 2014^162^ | Case-control study | Xinjiang | esophageal carcinoma | 117 | 2011.05-2012.01 | NR | PCR | 23 type-specific probes,including the high risk types (HPV16, 18, 31, 33, 35,39, 45, 51, 52, 53, 56, 58, 59, 66, 68, 73, 82, 83) and the low risk type (HPV6, 11, 42, 43, and 81) | esophageal carcinoma specimens in formalin-fixed paraffin-embedded archival tissues were collected |
| X.M. Lu 2008^163^ | cross-sectional study | Xinjiang | Esophageal squamous cell carcinoma (ESCC) | 41 | 1999 - 2004 | NR | PCR | HPV 6, 11, 16, 18, 31, 33-35, 39, 40, 42-45, 51-54, 56/74, 58, 59, 66, 68/73 and 70) | Paraffin blocks from the primary tumor and the normal mucosa were cut in 10 mm sections and four sections/sample were collected in microcentrifuge tubes |
| K Shuyama 2007^164^ | cross-sectional study | Gansu; Shandong | Esophageal squamous cell carcinoma (ESCC) | 48 | 1994-2005 | NR | PCR | 25 different HPV genotypes (no specific information) | Formalin-fixed, paraffin-embedded samples |
| Shu-Hsing Cheng 2017^165^ | Cohort study | Taiwan | HIV-infected (n=714): ASCUS :n=87(12.2%); LSIL/HSIL :n=88 (12.3%); | 714 | 2011.03-2016.06 | 30.66±8.18 | PCR | 12 oncogenic types (HPV 16, 18, 31, 33, 35, 39, 45, 51, 52, 56, 58, 59 ) ;24 non-oncogenic types (HPV 6, 11, 26, 40, 42, 53, 54, 55, 61, 62, 64, 66, 67, 69, 70, 71, 72, 73, 81, 82, 83, 84, IS39 and CP6108) | anal sampling |
| Wei Li 2007^166^ | Cohort study | Hong Kong | tonsillar cancers | 37 | 1985-2004 | NR | PCR | NR | crude lysates of 5 mm sections of formalin-fixed, paraffin-embedded tonsillar tumours |
| King-Yin Lam 1997^167^ | Cohort study | Hong Kong | Esophageal Squamous Carcinomas | 59 | 1992-1994 | NR | PCR | 2 high-risk types (HPV16,18) | One representative frozen block from the tumor |
| Jamil N. Al-Swiahb 2010^168^ | Cohort study | Taiwan | Oropharyngeal Cancer | 206 | 1992.01-2008.03 | NR | PCR | The primer sequences were the same as those previously reported by Resnick et al, flanking the conserved region L1 open readingframe that was found in a broad spectrum of HPV. | tissue sections |
| Hong-xue Meng 2018^169^ | Cohort study | Heilongjiang | Oropharyngeal Cancer :OPSCC | 1167 | 2000.01-2016.02 | NR | PCR | NR | Tissues were obtained from patients during surgery. |
| Lifang Cui 2019^170^ | Cohort study | Beijing | laryngeal squamous cell carcinoma (LSCC) | 307 | 2005-2011 | NR | PCR | 13 High Risk-HPV types (HPV-16,-18, -31,-33,-35,-39,-45,-51,-52,-56,-58,-59,-68) and two Low Risk-HPV types (HPV-6, -11) | Four pieces of paraffin sections (4 μm thickness) were prepared from each block of formalin-fixed, paraffin-embedded tissues |
| Enqi Wu 2019^171^ | Cohort study | Inner Mongolian | archival head and neck cancer (HNC) | 12 | 2009.10- 2012.12 | NR | PCR | 3 high-risk types (HPV16,58,31) | representative blocks of formalinfixed HNC from 75 cases and blocks |
| Shu-Hsing Cheng 2014^172^ | Cohort study | Taiwan | ASCUS: n=31； HSIL: n=6 | 53 | 2011.03-2011.12 | 31.0 ±6.8 | PCR | 13 oncogenic types(16, 18, 31, 33, 35, 39, 45, 51, 52, 56, 58, 59, 68); 24 non-oncogenic types(6, 11, 26, 40, 42, 53, 54, 55, 61, 62, 64, 66, 67, 69, 70, 71, 72, 73, 81, 82, 83, 84, IS39, CP6108) | Rectal swabs |
| Zhen-Qiang Sun 2013^173^ | Case-control study | Xinjiang | Rectal Cancer | 37 | 2007.12-2008.09 | NR | PCR | NR | tumor tissue |
| Kuan-Ting Kuo 2008^174^ | Cohort study | Taiwan | tonsillar squamous cell carcinoma | 79 | 1997.03-2005.03 | 51 (29-79) | PCR | 7 high-risk types (HPV16,18,33,35,58,66,69) | Formalin-fixed, paraffin-embedded tissue blocks of patients |
| Wen-Kang Liu 2010b^175^ | Cohort study | Shanxi | ESCC | 48 | 2000-2003 | NR | PCR | 1 high-risk types (HPV16) | specimens from patients routinely processed for fixation and embedding with formalin and paraffin |
| Jason Y.K. Chan 2022^176^ | Cohort study | Hong Kong | head and neck squamous cell carcinoma (HNSCC)-Larynx | 24 | 2015.10-2018.04 | NR | PCR | NR | Tumor tissues |
|  |  |  | head and neck squamous cell carcinoma (HNSCC)-Hypopharynx | 13 |  |  |  |  |  |
| Tian-You Ma 2007^177^ | cross-sectional study | Shaanxi | gastric carcinoma (GC) | 26 | 2003-2005 | NR | PCR | NR | specimens routinely processed for fixation in formalin and were embedded in paraffin |
| Hongzhi Wang 2015^178^ | Cohort study | Guangdong | laryngeal squamous cell carcinomas (LSCCs) | 312 | 1995 - 2009 | NR | PCR | 15 high-risk HPV subtypes (HPV 16, 18, 31, 33, 35, 39, 45,51, 52, 53, 56, 58, 59, 66, and 68) and 9 low-risk HPV (HPV 6, 11, 40, 42, 43, 44, 54, 70, and 81). | Formalin-fixed paraffin-embedded specimens were obtained from patients who received definitive surgery |
| Shih-Wei Yang 2009^179^ | cross-sectional study | Taiwan | oral cavity mucosa leukoplakia | 134 | 2002.07-2007.12 | NR | PCR | NR | Paraffin-embedded specimens from patients |
| Guoying Ni 2019^180^ | cross-sectional study | Guangdong+Zhejiang | head and neck squamous cell carcinomas (HNSCCs) | 247 | 2005 – 2018 (Guangzhou)  2009 – 2017 (Wenzhou)  2012 – 2017 (Foshan) | NR | PCR | 17 High Risk-HPV types (16, 18, 31, 33, 35, 39, 45, 51, 52, 53, 56, 58, 59, 66, 68, 73 and 82) and 6 LR-HPV types (6, 11, 42, 43, 81 and 83) | Five to seven slices (5 μm) from each sample were collected |
| PKS Chan 2017^181^ | Case-control study | Hong Kong | oesophageal squamous cell carcinoma (OSCC) | 112 | 2012.04-2015.03 | NR | PCR | NR | tissue samples |
| Jinn-Li Wang 2014^182^ | Cohort study | Taiwan | lung adenocarcinoma | 102 | 2003-2011 | NR | PCR | 2 High Risk-HPV types (16, 18) | cancer tissues |
| Li-Ang Lee 2013^183^ | Cohort study | Taiwan | oesophageal squamous cell carcinoma (OSCC) | 391 | 2010 - 2011、2005 - 2006 | NR | PCR | 13 High Risk-HPV types (16, 18, 31, 33, 35, 39, 45, 51, 52, 56, 58, 59, and 68)；25 low risk （HPV 6, 11, 26, 32, 37, 42–44,53–55, 61, 62, 66, 67, 69–72, 74, 81–84, and L1AE5 ) | formalin-fixed, paraffin-embedded (FFPE) specimens from patients with newly diagnosed OSCC |
| Yu-Feng Wang 2013^184^ | Case-control study | Henan | squamous cell carcinoma of the esophagus (ESCC) | 56 | 2007.12-2008.12 | NR | PCR | 24 types of specific HPV | frozen esophageal mucosa |
| Julia Yu-Fong Chang 2003^185^ | Case-control study | Taiwan | oral squamous cell carcinomas (OSCCs) | 42 | 1988.01-2002.08 | NR | PCR | 39 subtypes of HPV DNA (6, 11, 16, 18, 26, 31-33, 35, 37, 39, 42-45, 51-56, 58, 59, 61, 62, 66-70, 72, 74, 82, CP8061, CP8304, L1AE5, MM4, MM7, and MM8) | Formalin-fixed, paraffin-embedded tissue blocks |
| Hui-Ling Chiou 2003^186^ | Case-control study | Taiwan | primary lung cancer | 90 | NR | NR | PCR | 2 High Risk-HPV types (16, 18) | tumor tissues and peripheral blood samples available |
| Ming Zhang 2009^187^ | Case-control study | Hubei | Lung cancer | 49 | 2006.11—2007. 4 | NR | PCR | 2 High Risk-HPV types (16, 18) | Lung tissue specimens |
| Eddy WH Lam, FRCSEd2018^188^ | cross-sectional study | Hong Kong | laryngeal squamous cell carcinoma (LSCC) | 83 | 2005-2010 | NR | PCR | NR | paraffin-embedded tissues |
| Haizhong Zhang 2016^189^ | cross-sectional study | Hebei | Oropharyngeal squamous cell carcinoma OSCC | 28 | 2010.1-2014.12 | NR | PCR | NR | Tumor tissue |
| Yuanlin Liu 1995^190^ | Cohort study | Beijing | Penile intraepithelial neoplasia grade II | 10 | NR | NR | PCR | HPV6, 11,16,33 | Tissue samples or paraffin sections |
|  |  |  | Penile squamous cell carcinoma | 16 |  |  |  |  |  |
| Ling Liu 2014^191^ | cross-sectional study | Hebei | Esophageal squamous cell carcinoma | 90 | 2009-2012 | NR | PCR | 25 mucosal HPV types and 5 high-risk HPV types (16, 18, 45,56,58). 4 low risk (HPV6,11, 26,57) | Esophageal squamous cell carcinoma and paraffin-embedded mucosa |
| Xiaogang Du 2010^192^ | Case-control study | Shanxi | Esophageal squamous cell carcinoma | 29 | 2008 .8-2009.3 | NR | PCR | 2 High Risk-HPV types (16, 18) | Paraffin blocks of esophageal cancer specimens |
| Yihong Zhuang 2005^193^ | Case-control study | Guangdong | Penile cancer | 124 | 1997.4-2004.6 | Range：20-64  Mean: 41.1 | PCR | 2 High Risk-HPV types (16, 18) | Pathological tissue |
| Zhen Wang 2015^194^ | Cohort study | Shanghai | Oropharyngeal squamous cell carcinoma OPSCC | 171 | 2008.01-2014.04 | NR | PCR | 2 High Risk-HPV types (16, 18) | Pathological sections |
| LiFang Cui 2021^195^ | cross-sectional study | Beijing | Laryngeal squamous cell carcinoma LSCC | 307 | 2005.01-2011.12 | NR | PCR | 13 High Risk-HPV types (16, 18, 31,33, 35, 39,45, 51, 52, 56,58, 59,68)；2 Low risk （HPV 6,11) | Paraffin-embedded specimens of LSCC |
| Meiling Mao 2018^196^ | Cohort study | Beijing | spindle cell squamous cell carcinoma (SCSCC) of the larynx | 12 | 2007-2017 | NR | PCR | 21 HPV genotypes (6, 11, 16, 18, 31, 33, 35, 39, 42, 43, 44, 45, 51, 52, 53, 56, 58, 59, 66, 68, 81) | Pathological sections |
| Lifang Cui2018^197^ | Cross-sectional study | Beijing | Basaloid Squamous cell Carcinoma (BSCC) | 27 | 2005.01-2011.12 | NR | PCR | 13 high-risk HPV types (HPV16, 18, 31, 33, 35, 39, 45, 51, 256, 58, 59, 68) and 2 low-risk HPV types (HPV6, 11). | Pathological examination of laryngeal squamous cell carcinoma specimens |
| Heng‑Jui Chang 2023^198^ | Case-control study | Taiwan | Prostate cancer (PCa) | 143 | 2018.02-2020.12 | Median age (with interquartile range) was 71.8 (11.2)  Mean±SD(range):72.6±7.8（55-86） | PCR | HPV16, 18, 31, 33, 35, 39, 45, 51, 52, 56, 58, 59, 68, 73, 82 and HPV 6, 11, 53, 54, 61, 62, 66, 69, 70, 72, 81, 84 | formalin-fixed, paraffin-embedded tissue (FFPE) samples |
| Chun-Yi Chuang 2012^199^ | Cross-sectional study | Taiwan | Oral squamous cell carcinomas | 108 | 2001-2010 | NR | PCR | HPV 16,18 | OSCC tumor specimens |
| Shu-Hsing Cheng 2015^200^ | Cross-sectional study | Taiwan | Cases with high-grade AIN | 14 | 2011.03-2013.12 | 31.78 ± 10.29 | PCR | Thirty-seven types of HPV were detected, comprising oncogenic types 16, 18, 31, 33, 35, 39, 45, 51, 52, 56, 58, 59, and 68, and non-oncogenic types 6, 11, 26, 40, 42, 53, 54, 55, 61, 62, 64, 66, 67, 69, 70, 71, 72, 73, 81, 82, 83, 84, IS39, and CP6108. | Anal cytology samples |
| Yan Yu 2015^201^ | Case-control study | Xi'an | Lung cancer | 127 | 2012.01-2014.05 | NR | PCR | HPV 16,18 | The 4% paraformaldehyde-fixed and paraffin‑embedded tissue samples |
| Ming-Fang Wu 2005^202^ | Case-control study | Taiwan | Non small cell lung cancer (NSCLC) | 108 | 1993-2001 | NR | PCR | HPV 16,18 | surgically removed tissue |
| Dakai Zhang 2017^203^ | Cohort study | NR | Esophageal squamous cell carcinoma (ESCC) | 155 | 2009-2012 | NR | PCR | HPV 16,18 | formalin fixed paraffin embedded (FFPE) blocks by ESCC biopsy |
| Wen-Lun WANG 2015^204^ | Cohort study | Taiwan | Esophageal squamous cell carcinoma (ESCC) | 148 | 2008.06-2011.12 | NR | PCR | NR | formalin-fixed, paraffinembedded tumor samples |
| Gian Luca Armas 2008^205^ | Cross-sectional study | Taiwan | HNSCC head and neck cancer | 31 | 1987.01-2006.10 | NR | PCR | HPV 6, 11, 16, 18 | tissue block |
| Wan-Yu Lo 2007^206^ | Case-control study | Taiwan | Oral squamous cell carcinomas (OSCC) | 62 | 1990-2004 | NR | PCR | HPV 16, 18, 31, 33, 52b and 58；HPV 6，11,18 | Formalin-fixed paraffin-embedded blocks of tumor specimens that had been obtained from cancer patients who had undergone surgery for oral cancer were used in our study |
| Tsen-Fang Tsai 2008^62^ | Cross-sectional study | Taiwan | anal high-grade squamous intraepithelial lesion (AHSIL) | 8 | 1991-2005 | NR | PCR | 39 types of HPV DNA (6, 11, 16, 18, 26, 31, 32, 33, 35, 37, 39, 42, 43, 44, 45, 51, 52, 53, 54, 55, 56, 58, 59, 61, 62, 66, 67, 68, 69, 70, 72, 74, 82, CP8061, CP8304, L1AE5, MM4, MM7, and MM8) | Paraffin embedded tissue sections |
|  |  |  | squamous cell carcinoma (SCC) | 8 |  |  |  |  |  |

Table S5 Summary of data from studies reporting HPV-related cancer and precancer

| **Cancer** | | **Study ID** | **Sample size** | **Positive rate of any genotype** | **Type** | **mRNA test (E6, E7, E2, etc）** | | | **Immunohistochemistry （biomarker-p16INK4a, pRb, p53, Cyclin D1）** | | |
| --- | --- | --- | --- | --- | --- | --- | --- | --- | --- | --- | --- |
|  |  |  |  |  |  |  | **HPV+ group** | **Total** |  | **HPV+ group** | **Total** |
|  |  |  |  |  |  | **mRNA** | **Positive rate** | **Positive rate** | **biomarker** | **Positive rate** | **Positive rate** |
| **Cancer of the head and neck** | **Oral cancer** | Sun 2013^173^ | 391 | 20.72% | NR | NR | NR | NR | NR | NR | NR |
|  |  | Z.-y. Zhang 2004^117^ | 48 | 81.25% | NR | NR | NR | NR | NR | NR | NR |
|  |  | Lei Lei 1996^127^ | 17 | 58.82% | NR | NR | NR | NR | NR | NR | NR |
|  |  | Timan Pa 2000^132^ | 48 | 81.25% | NR | NR | NR | NR | NR | NR | NR |
|  |  | Xiao-Jie Chen 2016^128^ | 59 | 0.00% | NR | NR | NR | NR | NR | NR | NR |
|  |  | S-F Huang 2012^130^ | 96 | 29.17% | NR | NR | NR | NR | NR | NR | NR |
|  |  | Dan Zhao 2009^142^ | 35 | 42.86% | NR | NR | NR | NR | NR | NR | NR |
|  |  | Hui Huang 2012^143^ | 61 | 11.48% | NR | NR | NR | NR | NR | NR | NR |
|  |  | Julia Yu-Fong Chang 2003^185^ | 42 | 33.33% | NR | NR | NR | NR | NR | NR | NR |
|  |  | Haizhong Zhang 2016^189^ | 28 | 17.86% | NR | NR | NR | NR | NR | NR | NR |
|  |  | Chun-Yi Chuang 2012^199^ | 108 | 35.19% | HPV 16,18 | IL-10 | NR | 47.22% | NR | NR | NR |
|  |  | Wan-Yu Lo 2007^206^ | 62 | 12.90% | NR | NR | NR | NR | NR | NR | NR |
|  |  | Fa Chen 2016^106^ | 178 | 14.04% | NR | NR | NR | NR | NR | NR | NR |
|  |  | Li-Ang Lee 2013^183^ | 391 | 20.70% | NR | NR | NR | NR | NR | NR | NR |
|  |  | Li-Ang Lee 2015^116^ | 938 | 19.40% | NR | NR | NR | NR | NR | NR | NR |
|  |  | C.-W. Luo 2007^118^ | 48 | 25.00% | HPV 33,52,18,53,11,18,16,39,72,66,58 | NR | NR | NR | NR | NR | NR |
|  | **Precancerous lesions of oral cancer** | C.-W. Luo 2007^118^ | 45 | 31.11% | HPV 58,18,31,11,16,66,53,68,56,42,44,52,MM8, CP8304 | NR | NR | NR | NR | NR | NR |
|  |  | Shih-Wei Yang 2009^179^ | 134 | 22.39% | NR | NR | NR | NR | NR | NR | NR |
|  | **Nasal cancer** | Chunyan Hu 2020^114^ | 84 | 8.33% | HPV 16 | NR | NR | NR | p16 | 2.63% | NR |
|  | **Nasopharyngeal carcinoma** | Cheng-Chih Huang 2011^140^ | 36 | 27.78% | NR | NR | NR | NR | NR | NR | NR |
|  | **Cancer of the tonsil** | Chih-Yen Chien 2007^122^ | 100 | 6.00% | NR | NR | NR | NR | NR | NR | NR |
|  |  | Wei Li 2007^166^ | 37 | 8.11% | HPV 16 | NR | NR | NR | NR | NR | NR |
|  |  | Kuan-Ting Kuo 2008^174^ | 79 | 70.89% | HPV16, 18, 33, 35, 58, 66, 69 | NR | NR | NR | p16INK4A | NR | 50.63% |
|  | **Ear cancer** | Ying-Tai Jin 1997^113^ | 14 | 78.57% | HPV 16 | NR | NR | NR | NR | NR | NR |
|  | **Laryngeal cancer** | Fangjia Tong 2018^109^ | 211 | 62.43% | HPV 16 | NR | NR | NR | p16 | NR | 55.24% |
|  |  | B. Liu 2010^110^ | 84 | 27.38% | HPV 16/18 | NR | NR | NR | NR | NR | NR |
|  |  | X. Chen 2017a^135^ | 220 | 6.82% | HPV 16 | NR | NR | NR | NR | NR | NR |
|  |  | Xiaoling Min 2017-1^139^ | 29 | 31.03% | NR | NR | NR | NR | NR | NR | NR |
|  |  | Yanan Xu 2014^144^ | 654 | 5.05% | HPV 16,18,31,33,45 | NR | NR | NR | NR | NR | NR |
|  |  | Wei-Chih Chen 2017b^159^ | 103 | 13.59% | NR | NR | NR | NR | NR | NR | NR |
|  |  | Lifang Cui 2019^170^ | 307 | 12.38% | NR | NR | NR | NR | p16 | NR | 9.77% |
|  |  |  |  |  |  |  |  |  | p53 | NR | 33.55% |
|  |  |  |  |  |  |  |  |  | p21 | NR | 40.72% |
|  |  |  |  |  |  |  |  |  | Cyclin D1 | NR | 59.60% |
|  |  | Jason Y.K. Chan 2022^176^ | 24 | NR | HPV 6,16,18 | NR | NR | NR | NR | NR | NR |
|  |  | Hongzhi Wang 2015 | 312 | 8.97% | NR | NR | NR | NR | p16 | 1.28% | NR |
|  |  | Eddy WH Lam, FRCSEd2018^188^ | 83 | 1.20% | HPV 6 | NR | NR | NR | p16 |  | 12.04% |
|  |  |  |  |  |  |  |  |  | p53 |  | 56.62% |
|  |  | Lifang Cui2021^195^ | 307 | NR | HPV 16,51,52,33 | E6/E7 | 0.00% | 0.00% | p16 |  | 9.77% |
|  |  | Meiling Mao 2018^196^ | 12 | 0.00% | NR | NR | NR | NR | p53 |  | 100% |
|  |  | Lifang Cui2018^197^ | 29 | 29.60% | HPV 16 | NR | NR | NR | p16 | 11.10% | 18.52% |
|  | **Precancerous lesions of the larynx** | Xiaoling Min 2017-2^139^ | 15 | 73.33% | NR | NR | NR | NR | NR | NR | NR |
|  | **Cancers of the larynx and hypopharynx** | Lixiao Chen 2021^133^ | 110 | 16.36% | HPV 6,16,18,11,33 | NR | NR | NR | NR | NR | NR |
|  | **Oropharyngeal Cancer** | Hong-xue Meng 2020^126^ | 1197 | 5.76% | NR | NR | NR | NR | p16 |  | 6.01% |
|  |  | Eddy WH Lam 2015^147^ | 178 | 19.10% | NR | E6/7 | NR | 19.10% | NR | NR | NR |
|  |  | Jamil N. Al-Swiahb 2010^168^ | 206 | 11.65% | NR | NR | NR | NR | NR | NR | NR |
|  |  | Hong-xue Meng 2018^169^ | 1167 | 5.91% | NR | NR | NR | NR | p16INK4a |  | 6.17% |
|  |  | Guoying Ni 2019^180^ | 71 | 40.85% | NR | NR | NR | NR | NR | NR | NR |
|  |  | Zhen Wang 2015^194^ | 171 | 9.36% | HPV 16,18 | NR | NR | NR | p16INK4a |  | 28.00% |
|  |  | Hui Huang 2012^143^ | 61 | 11.48% | NR | NR | NR | NR | NR | NR | NR |
|  | **Tongue cancer** | Kunsong Huang 2020^120^ | 83 | 20.48% | NR | NR | NR | NR | NR | NR | NR |
|  | **Hypopharyngeal carcinoma** | Jason Y.K. Chan 2022^176^ | 13 | NR | HPV 6,16,18 | NR | NR | NR | NR | NR | NR |
|  | **Pharyngeal carcinoma** | Wuhao Lu 2013^136^ | 26 | NR | HPV 16,18,33,52 | NR | NR | NR | NR | NR | NR |
|  |  | Zhen Wang 2016^141^ | 168 | 9.52% | HPV 16 | NR | NR | NR | p53 | 0% | NR |
|  | **Head and Neck Cancer** | Xiangrui Ma 2017^121^ | 111 | 24.32% | HPV 16 | NR | NR | NR | NR | NR | NR |
|  |  | Bei Chen 2004^124^ | 51 | 13.73% | HPV 16 | NR | NR | NR | pRb | 3.92% | NR |
|  |  | Jun-Quan Yang 2019^125^ | 261 | 18.77% | NR | NR | NR | NR | NR | NR | NR |
|  |  | Wei Wei 2012^134^ | 84 | 40.48% | NR | NR | NR | NR | NR | NR | NR |
|  |  | Yu Long 2011^153^ | 59 | 11.86% | NR | NR | NR | NR | NR | NR | NR |
|  |  | Enqi Wu 2019^171^ | 12 | 16.67% | NR | NR | NR | NR | NR | NR | NR |
|  |  | Gian Luca Armas2008^205^ | 31 | NR | HPV 16,18 | NR | NR | NR | NR | NR | NR |
| **Lung cancer** | | Fei He 2019^115^ | 87 | 9.20% | NR | NR | NR | NR | NR | NR | NR |
|  |  | Yongping Wang 2008^155^ | 237 | 46.41% | NR | NR | NR | NR | NR | NR | NR |
|  |  | Xiaohong Fan 2014^160^ | 70 | 7.14% | NR | NR | NR | NR | NR | NR | NR |
|  |  | Ya Wen Cheng 2004^161^ | 96 | 36.46% | HPV 6 | NR | NR | NR | NR | NR | NR |
|  |  | Jinn-Li Wang 2014^182^ | 102 | 36.27% | NR | NR | NR | NR | NR | NR | NR |
|  |  | Hui-Ling Chiou 2003^186^ | 90 | NR | HPV 16/18 | NR | NR | NR | NR | NR | NR |
|  |  | Ming Zhang 2009^187^ | 49 | 42.86% | NR | NR | NR | NR | NR | NR | NR |
|  |  | Yan Yu 2015^201^ | 127 | 37.01% | HPV 16 | NR | NR | NR | NR | NR | NR |
|  |  | Ming-Fang Wu 2005^202^ | 108 | 44.44% | NR | NR | NR | NR | p16INK4a | NR | 50.92% |
| **Anal cancer** | **Anal cancer** | Tsen-Fang Tsai 2008^62^ | 7 | 100.00% | HPV 6,11,16,58 | NR | NR | NR | NR | NR | NR |
|  | **Anal precancerous lesions** | Tsen-Fang Tsai 2008^62^ | 8 | 100.00% | HPV 6,11,16,58,18,33,51 | NR | NR | NR | NR | NR | NR |
|  |  | Shu-Hsing Cheng 2014^172^ | 53 | 96.20% | NR | NR | NR | NR | NR | NR | NR |
|  |  | Shu-Hsing Cheng 2015^200^ | 14 | 92.86% | NR | NR | NR | NR | NR | NR | NR |
|  |  | Shu-Hsing Cheng 2017^165^ | 88 | 93.10% | HPV 6,11,16,18,31,33,45,52,58 | NR | NR | NR | NR | NR | NR |
| **Bladder cancer** | | Yongji Yan 2020^154^ | 122 | NR | HPV 16,18,33,39 | NR | NR | NR | NR | NR | NR |
|  |  | Longjia Qu 2010^149^ | 56 | 62.50% | NR | NR | NR | NR | NR | NR | NR |
| **Skin cancer** | | Yanan Xu 2017^151^ | 201 | 66.67% | NR | NR | NR | NR | NR | NR | NR |
|  |  | Zhen Wang 2014^152^ | 196 | 66.33% | NR | NR | NR | NR | NR | NR | NR |
| **Prostate Cancer** | | Lin Huang 2016^131^ | 75 | 34.67% | HPV 16,18 | NR | NR | NR | NR | NR | NR |
|  |  | Heng‑Jui Chang 2023^198^ | 143 | 6.99% | HPV 18,52 | NR | NR | NR | NR | NR | NR |
| **Esophageal cancer** | | Lianghai Wang 2016^107^ | 78 | 46.15% | NR | NR | NR | NR | p16INK4A | NR | 17.47% |
|  |  |  |  |  |  |  |  |  | p53 | NR | 90.57% |
|  |  | Dong-Hong Zhang 2011a^108^ | 70 | 40.00% | HPV 16 | NR | NR | NR | NR | NR | NR |
|  |  | De He 1997^111^ | 127 | 22.83% | HPV 16,18 | NR | NR | NR | NR | NR | NR |
|  |  | Shuying Li 2018^146^ | 136 | 40.44% | HPV 16 | NR | NR | NR | NR | NR | NR |
|  |  | Tao Liu 2013^148^ | 153 | NR | HPV 16,18 | NR | NR | NR | NR | NR | NR |
|  |  | J. M. Hu 2013^156^ | 93 | 37.63% | HPV 16 | NR | NR | NR | NR | NR | NR |
|  |  | Qing Ying Zhang 2011b^157^ | 71 | 73.24% | NR | NR | NR | NR | NR | NR | NR |
|  |  | Shuying Li 2017^158^ | 136 | 40.44% | HPV 16 | NR | NR | NR | NR | NR | NR |
|  |  | K Shuyama 2007^164^ | 48 | 33.33% | NR | NR | NR | NR | NR | NR | NR |
|  |  | X.M. Lu 2008^163^ | 41 | 24.39% | NR | NR | NR | NR | NR | NR | NR |
|  |  | Xiaobin Cui 2014^162^ | 117 | 29.06% | NR | NR | NR | NR | NR | NR | NR |
|  |  | King-Yin Lam 1997^167^ | 59 | 10.17% | HPV 16,18 | NR | NR | NR | NR | NR | NR |
|  |  | Wen-Kang Liu 2010b^175^ | 48 | 54.17% | HPV 16 | NR | NR | NR | NR | NR | NR |
|  |  | PKS Chan 2017^181^ | 112 | 3.57% | HPV 16 | E6 | HPV 16: 0% | NR | p16INK4a | 0.00% | NR |
|  |  | Yu-Feng Wang 2013^184^ | 56 | 16.07% | NR | NR | NR | NR | NR | NR | NR |
|  |  | Ling Liu 2014^191^ | 90 | 18.89% | NR | NR | NR | NR | NR | NR | NR |
|  |  | Xiaogang Du 2010^192^ | 29 | 41.38% | NR | NR | NR | NR | p53 | NR | 65.51% |
|  |  | Dakai Zhang 2017^203^ | 155 | 36.77% | NR | NR | NR | NR | p53 | 32.10% | NR |
|  |  | Wen-Lun WANG 2015^204^ | 148 | 17.57% | NR | NR | NR | NR | NR | NR | NR |
| **Gastric cancer** | | Tian-You Ma 2007^177^ | 26 | 42.31% | HPV 16 | NR | NR | NR | NR | NR | NR |
| **Penile cancer** | **Penile cancer** | Chengbiao Chu 2020^112^ | 226 | 32.74% | HPV 6,16,18 | NR | NR | NR | P16INK4a | NR | 22.57% |
|  |  | Weijie Gu 2019^119^ | 340 | 48.82% | HPV 16,18,31,33,35,45,51,52,56,58,59,6,11 | NR | NR | NR | P16INK4a | 35.60% | 45.60% |
|  |  | Bin Lang 2023^123^ | 103 | 72.82% | HPV 16,33,31,58,39,18,45,6,11 | NR | NR | NR | NR | NR | NR |
|  |  | Jianpo Zhai 2013^129^ | 28 | 25.00% | HPV 16 | NR | NR | NR | NR | NR | NR |
|  |  | K W Chan 1994^145^ | 41 | 14.63% | HPV 16,18 | NR | NR | NR | NR | NR | NR |
|  |  | Qiang Ding 1996^150^ | 28 | 60.71% | HPV 16,18 | NR | NR | NR | NR | NR | NR |
|  |  | Yuanlin Liu 1995-2^190^ | 16 | NR | HPV 16,18,33 | NR | NR | NR | NR | NR | NR |
|  |  | Yihong Zhuang 2005^193^ | 124 | 59.68% | NR | NR | NR | NR | NR | NR | NR |
|  | **Precancerous lesions of penile cancer** | Yuanlin Liu 1995-1^190^ | 10 | 100.00% | HPV 16,18,6 | NR | NR | NR | NR | NR | NR |
| **Rectal cancer** | | Na Zhao 2010^137^ | 53 | NR | HPV 16,51 | NR | NR | NR | NR | NR | NR |
|  |  | Zhen-Qiang Sun 2013^173^ | 37 | 67.57% | NR | NR | NR | NR | NR | NR | NR |

**Supplementary 5 Subgroup analysis of publication time**


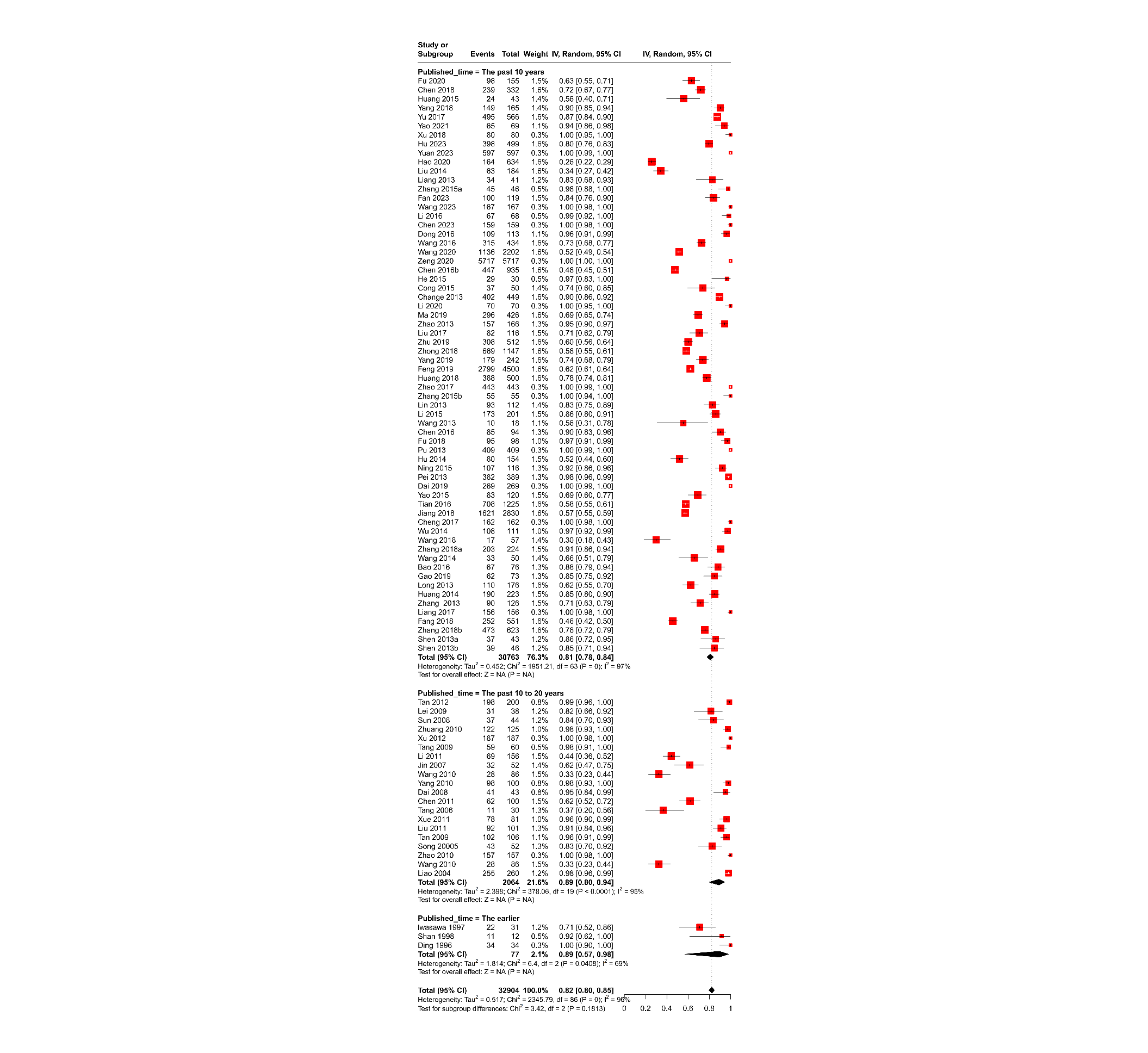


Figure S1 Forst plot of prevalence of any type of HPV among male patients with genital warts


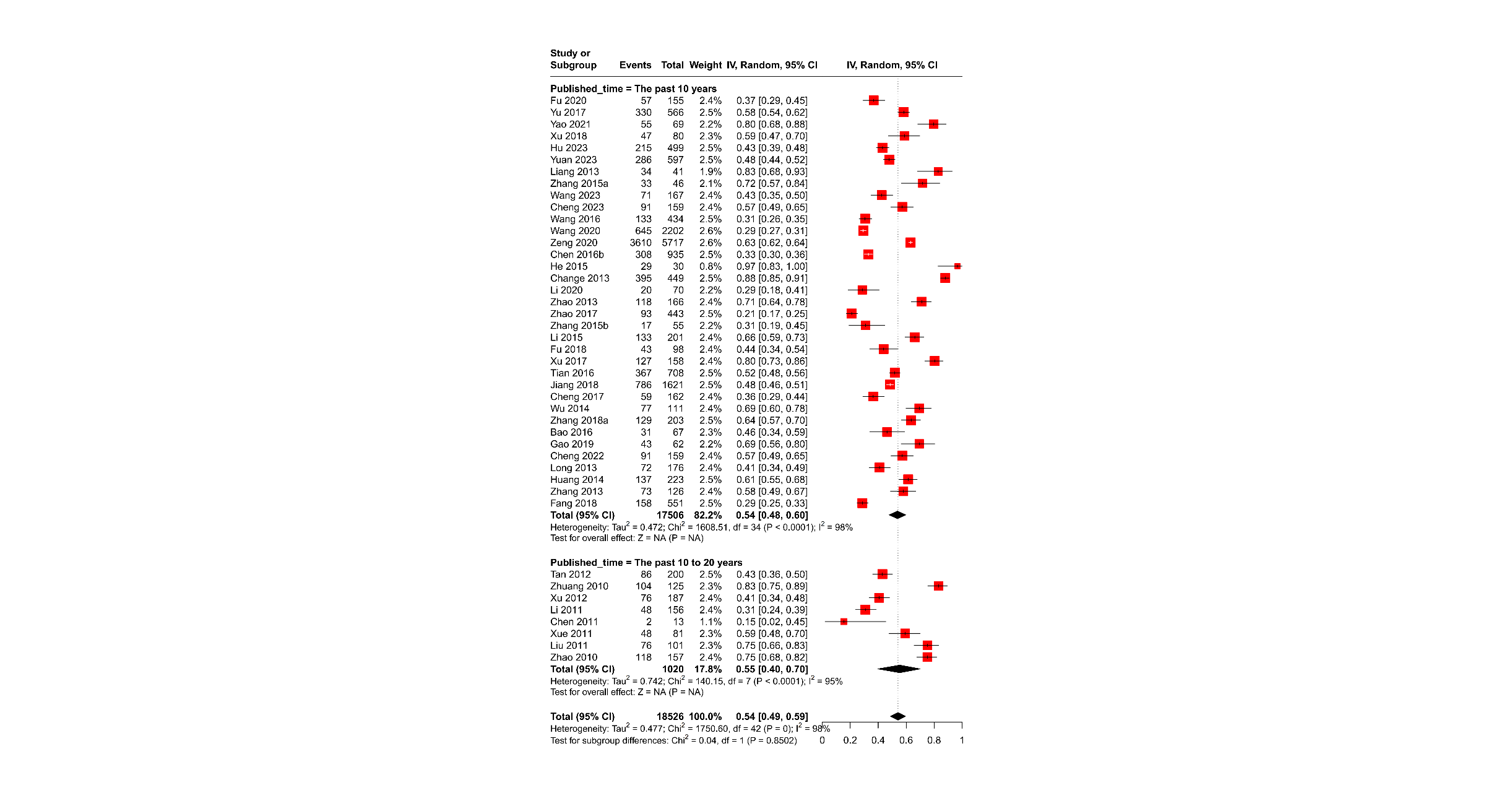


Figure S2 Forst plot of prevalence of single infection of HPV among male patients with genital warts


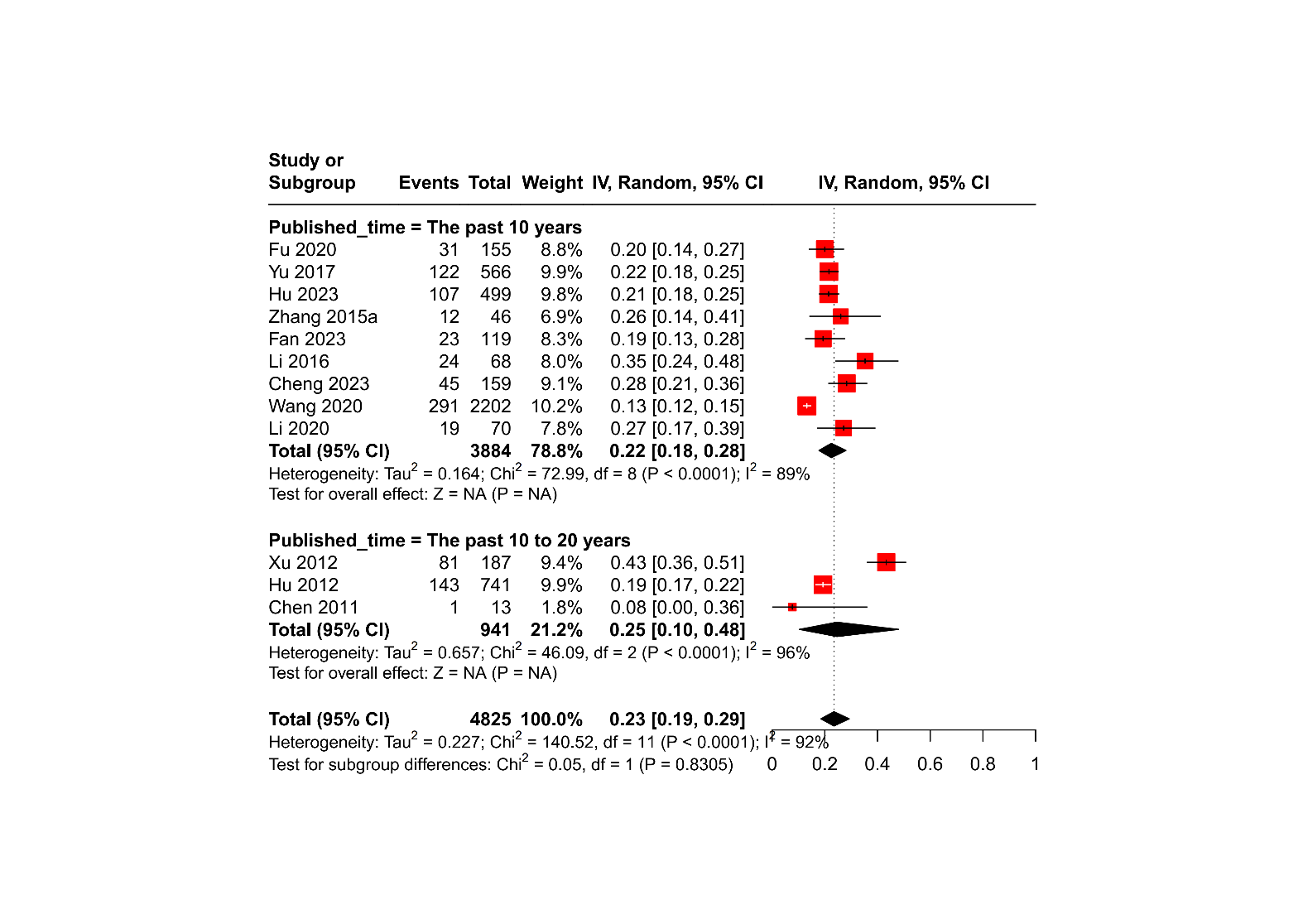


Figure S3 Forst plot of prevalence of double infection of HPV among male patients with genital warts


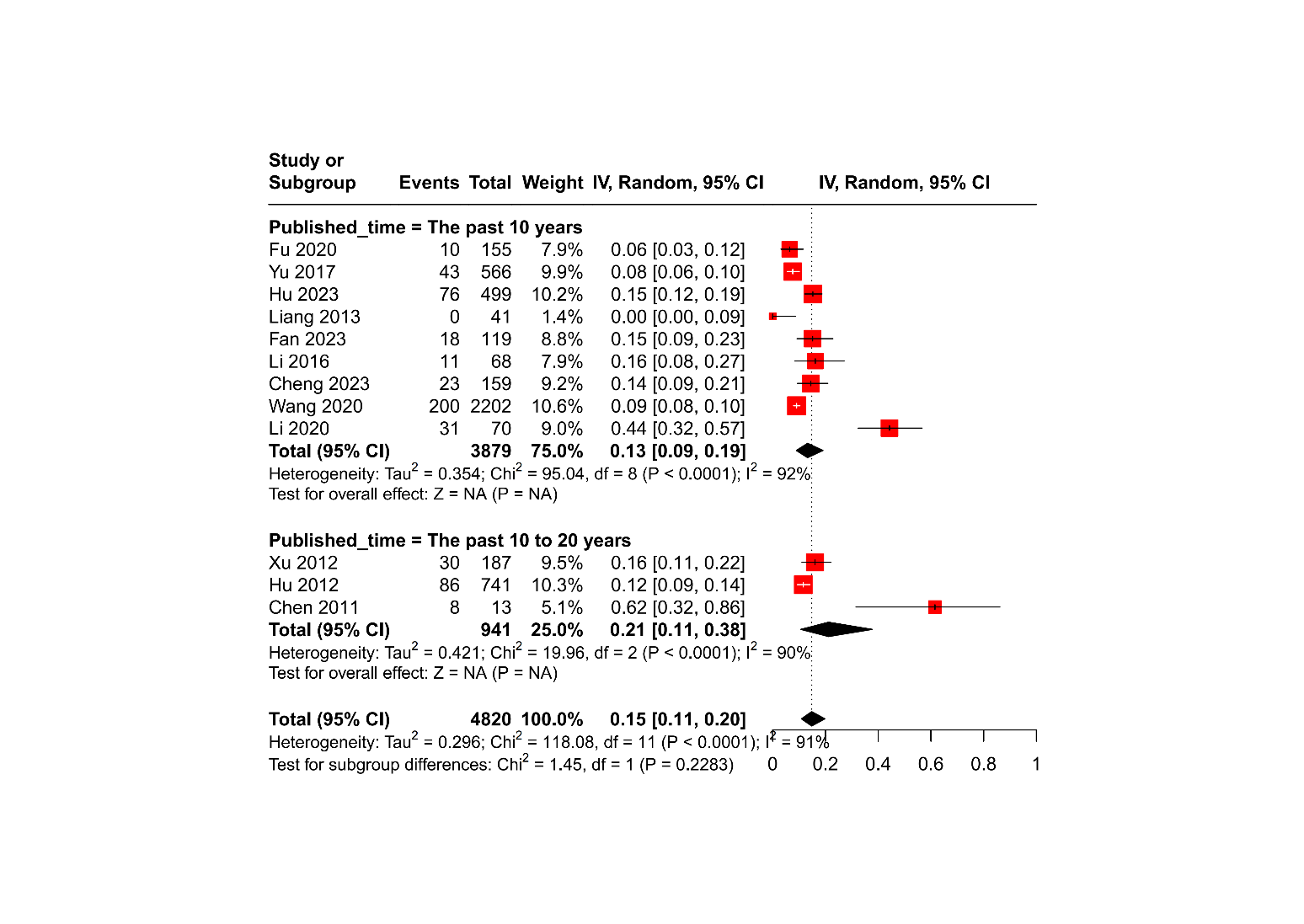


Figure S4 Forst plot of prevalence of multiple infection of HPV among male patients with genital warts


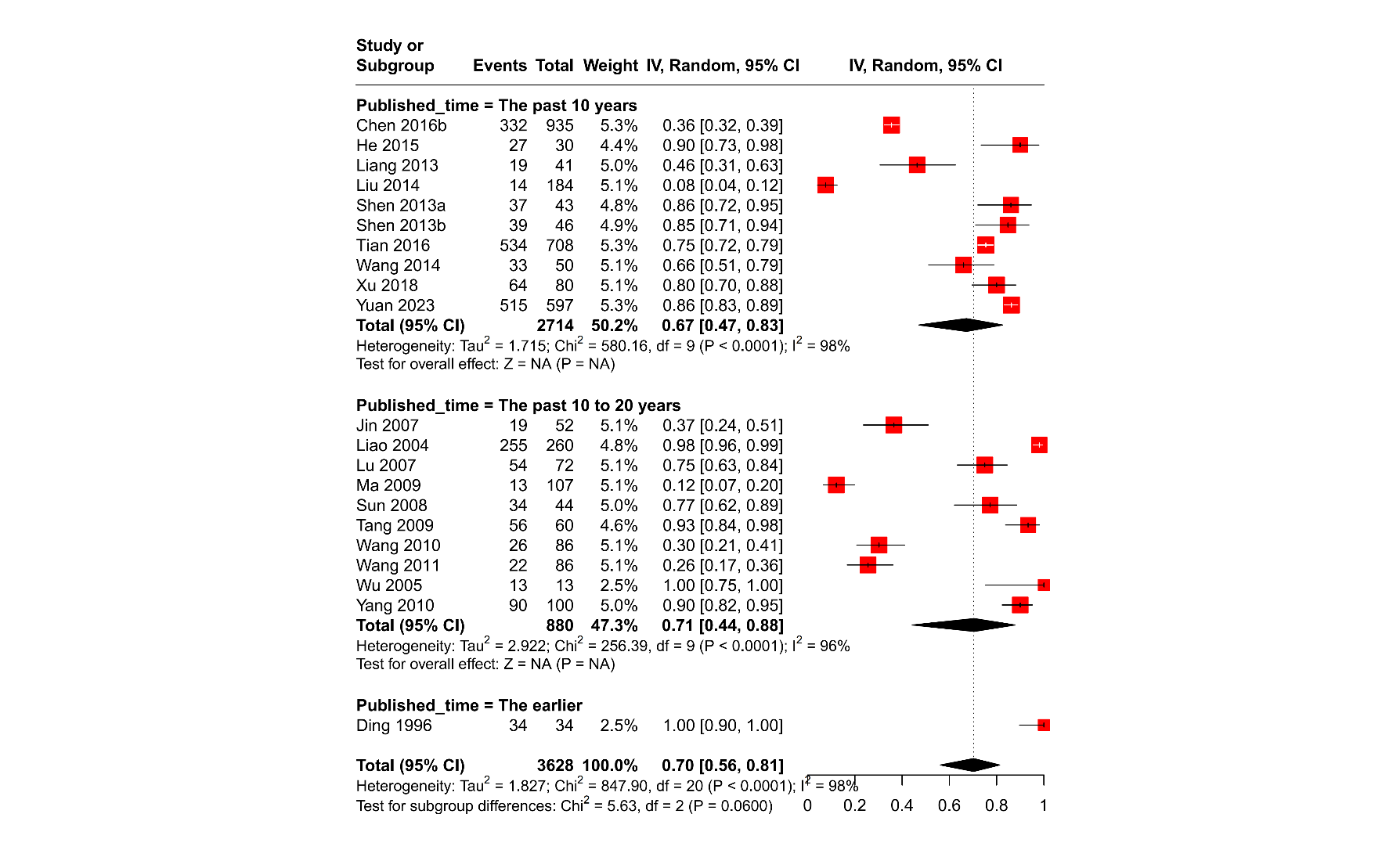


Figure S5 Forst plot of prevalence of HPV 6/11 infection among male patients with genital warts

**Supplementary 6 Publication bias assessment results**


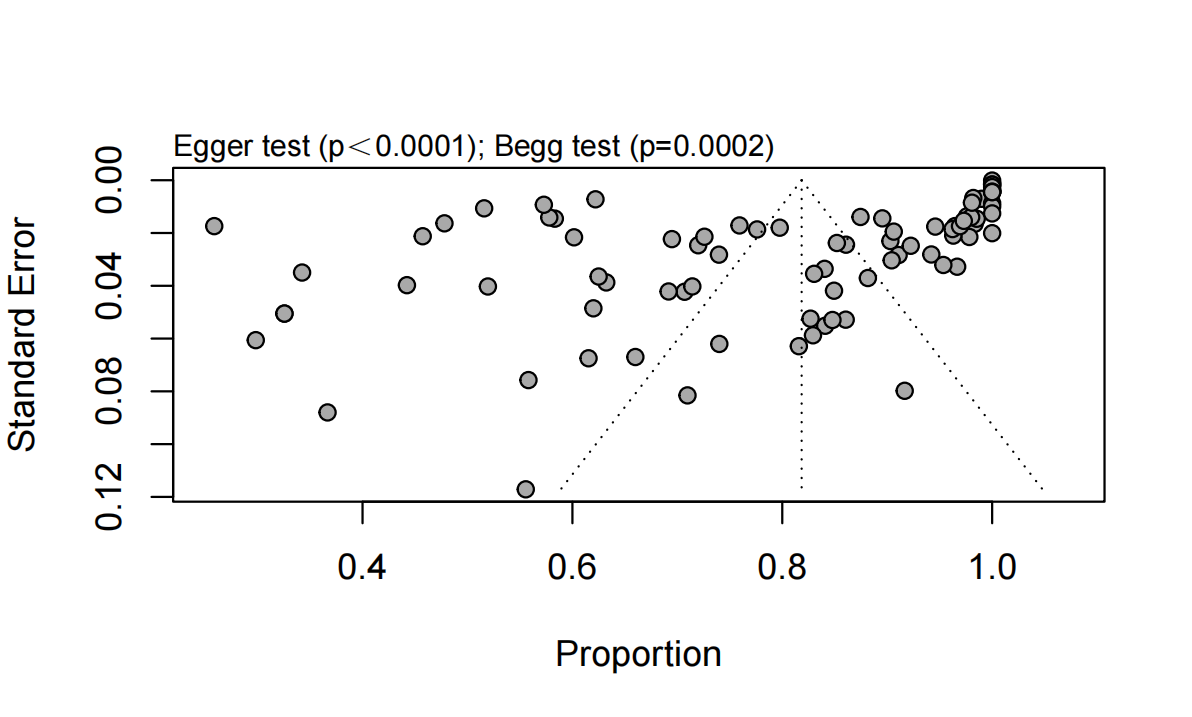


Figure S6 Funnel plot of prevalence of any type of HPV among male patients with genital warts


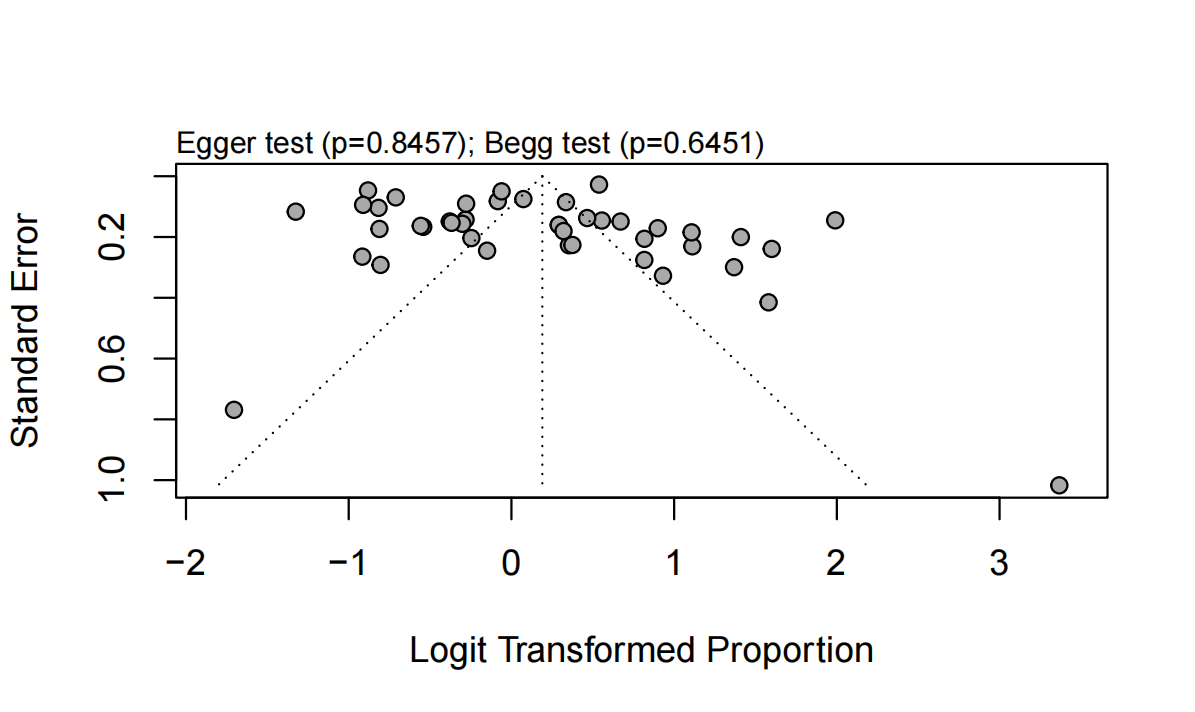


Figure S7 Funnel plot of prevalence of single infection of HPV among male patients with genital warts


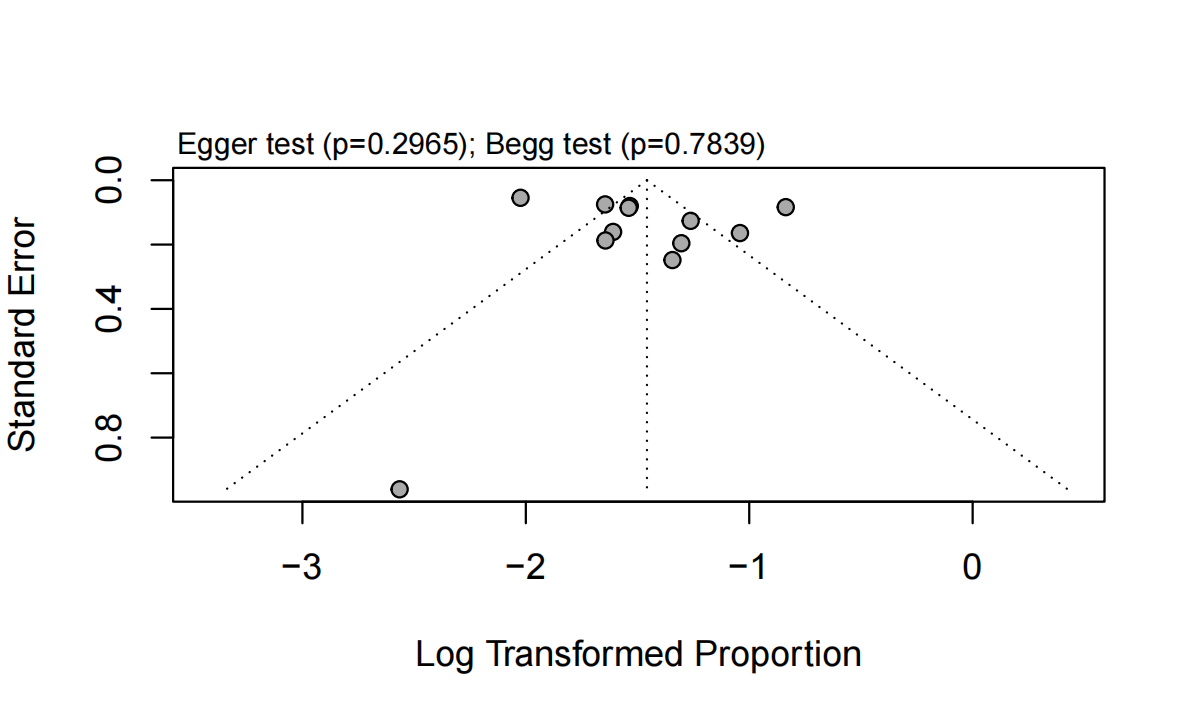


Figure S8 Funnel plot of prevalence of double infection of HPV among male patients with genital warts


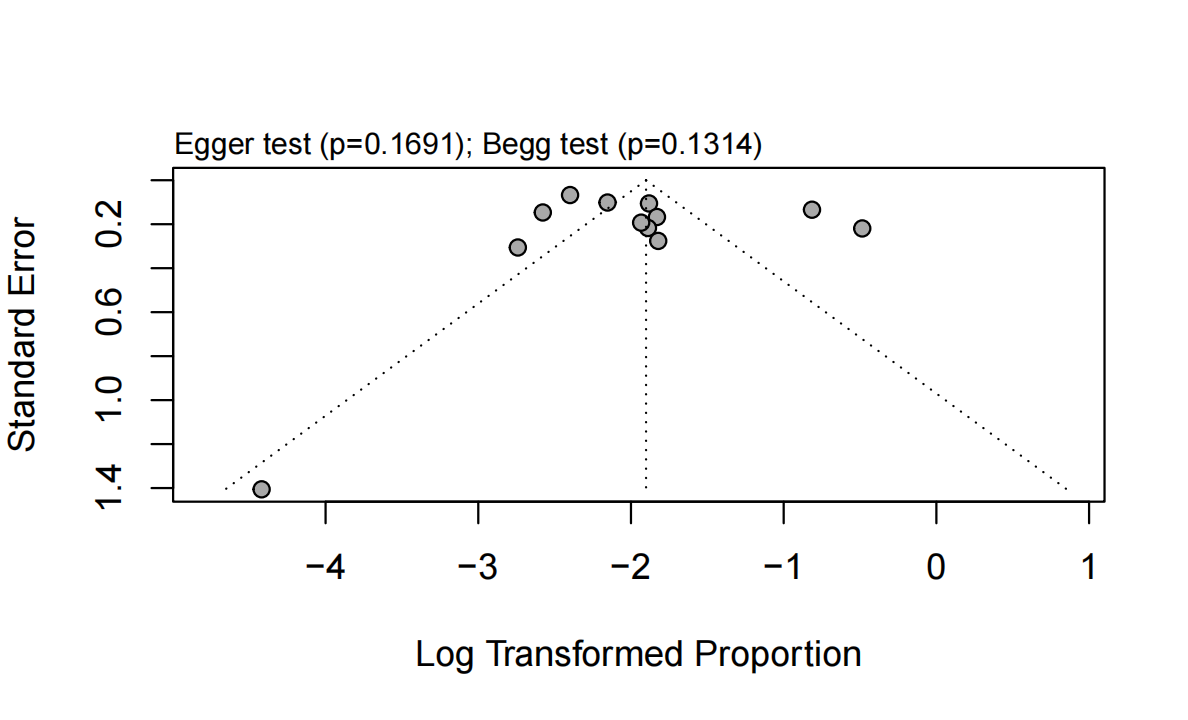


Figure S9 Funnel plot of prevalence of multiple infection of HPV among male patients with genital warts


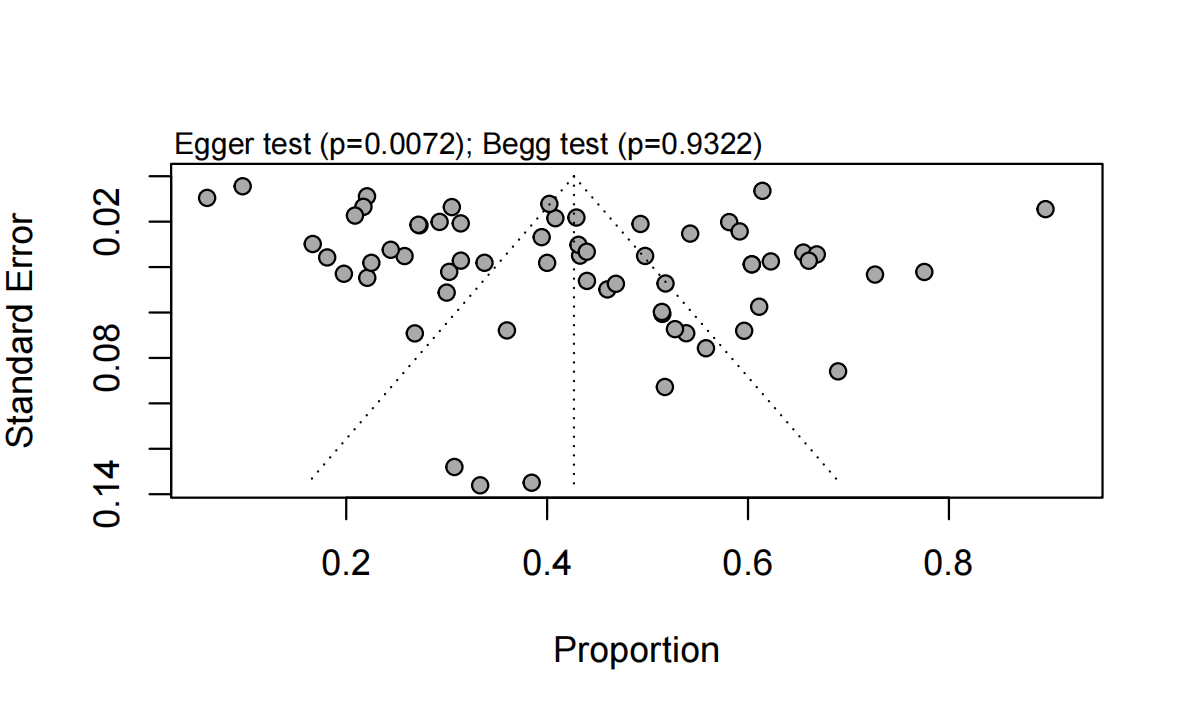


Figure S10 Funnel plot of prevalence of HPV 6 among male patients with genital warts


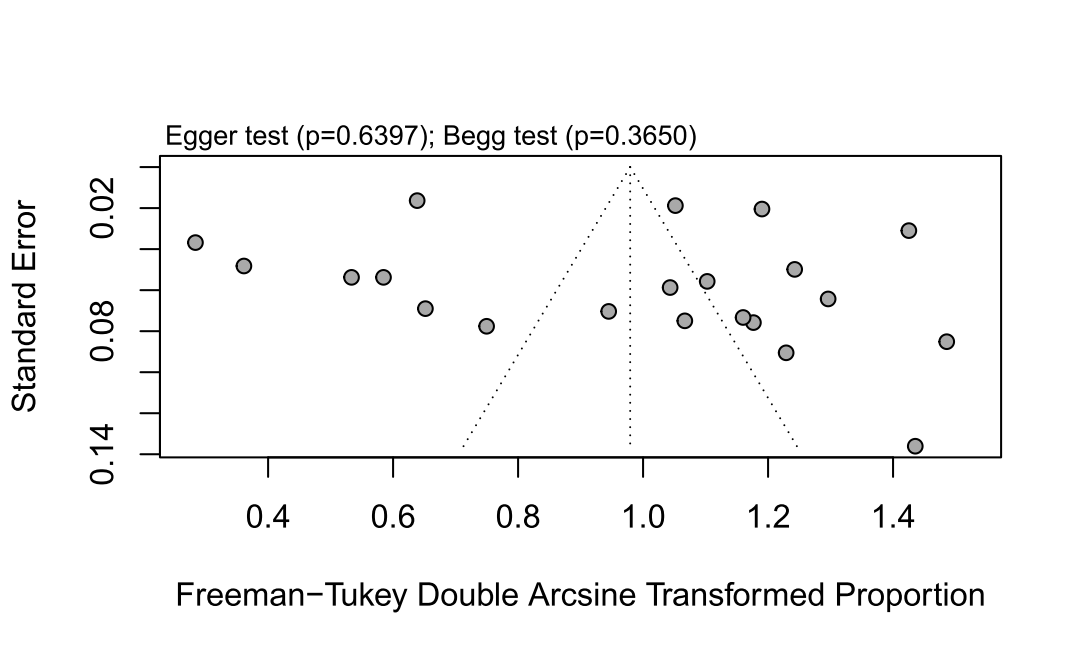


Figure S11 Funnel plot of prevalence of HPV 6/11 among male patients with genital warts


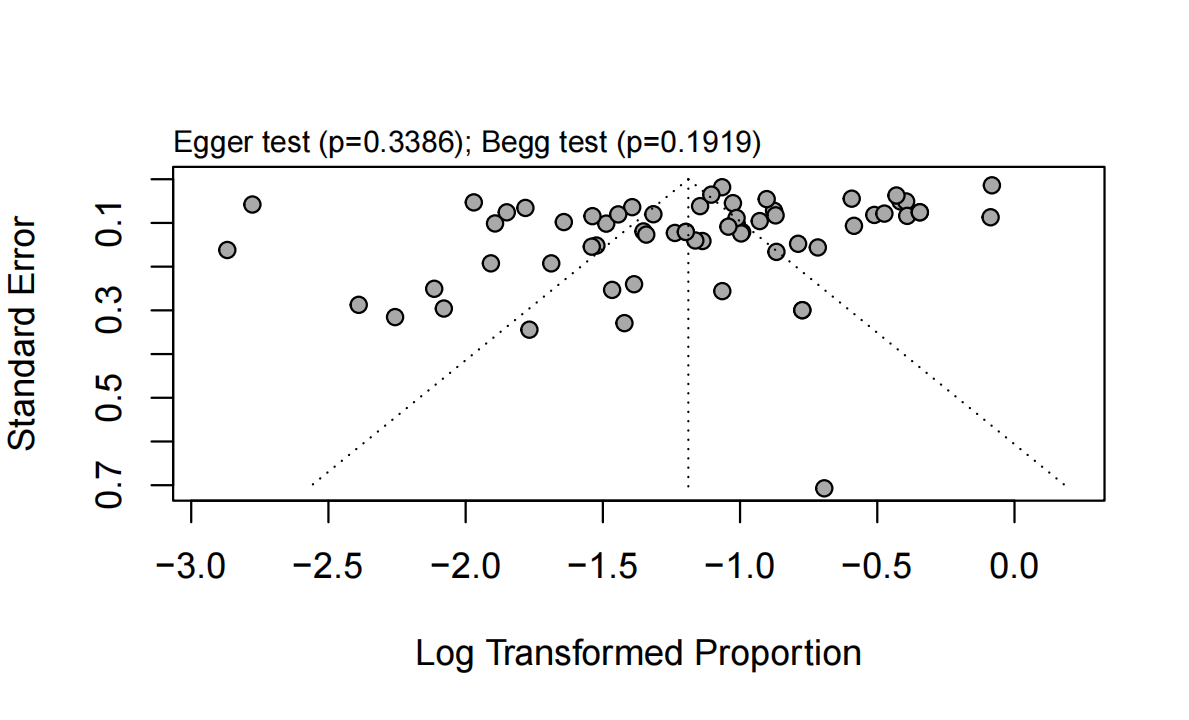


Figure S12 Funnel plot of prevalence of HPV 11 among male patients with genital warts


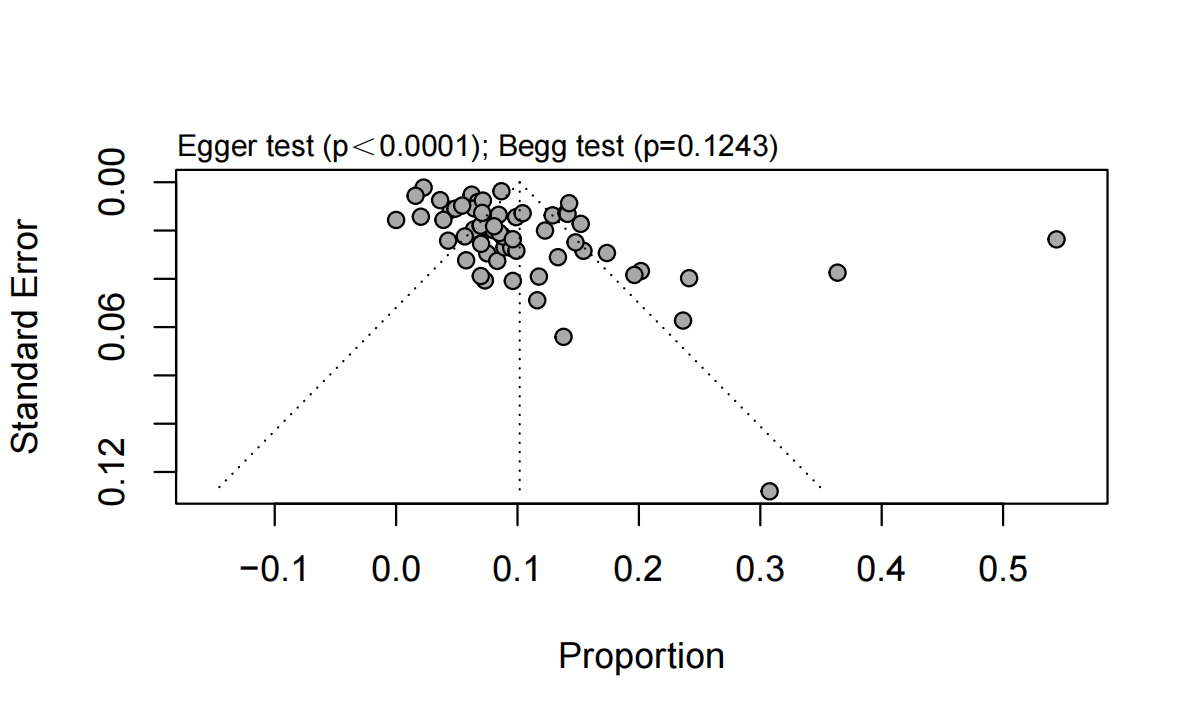


Figure S13 Funnel plot of prevalence of HPV 16 among male patients with genital warts


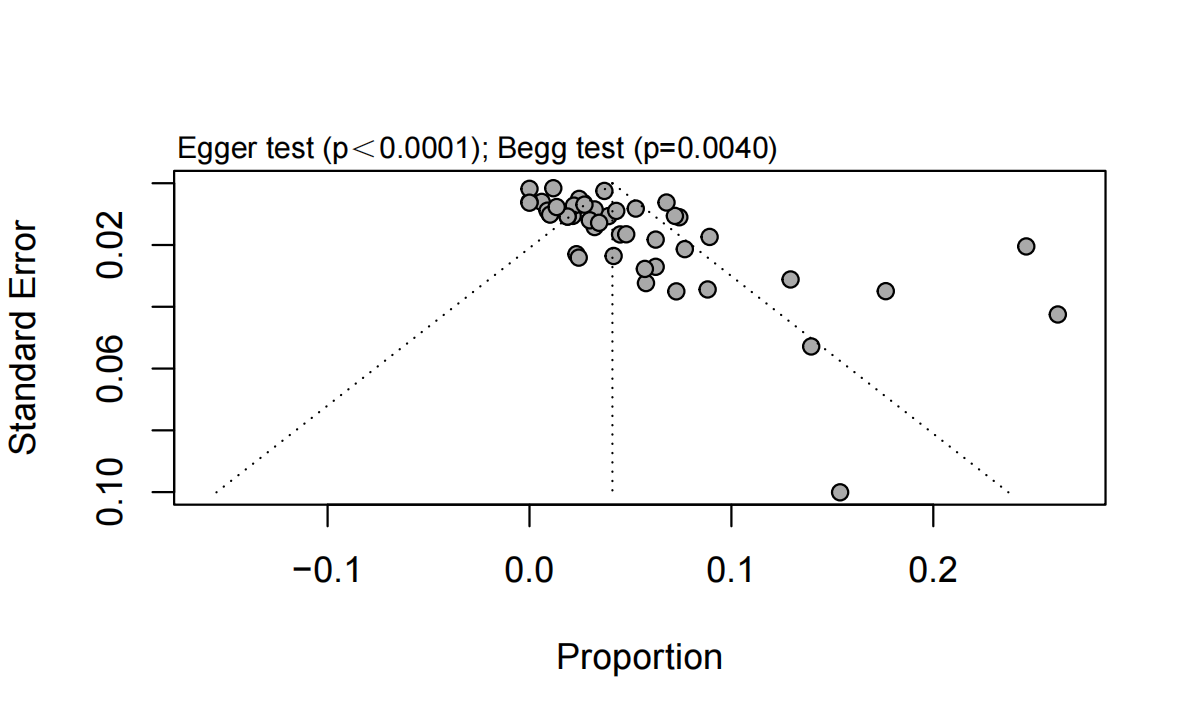


Figure S14 Funnel plot of prevalence of HPV 18 among male patients with genital warts


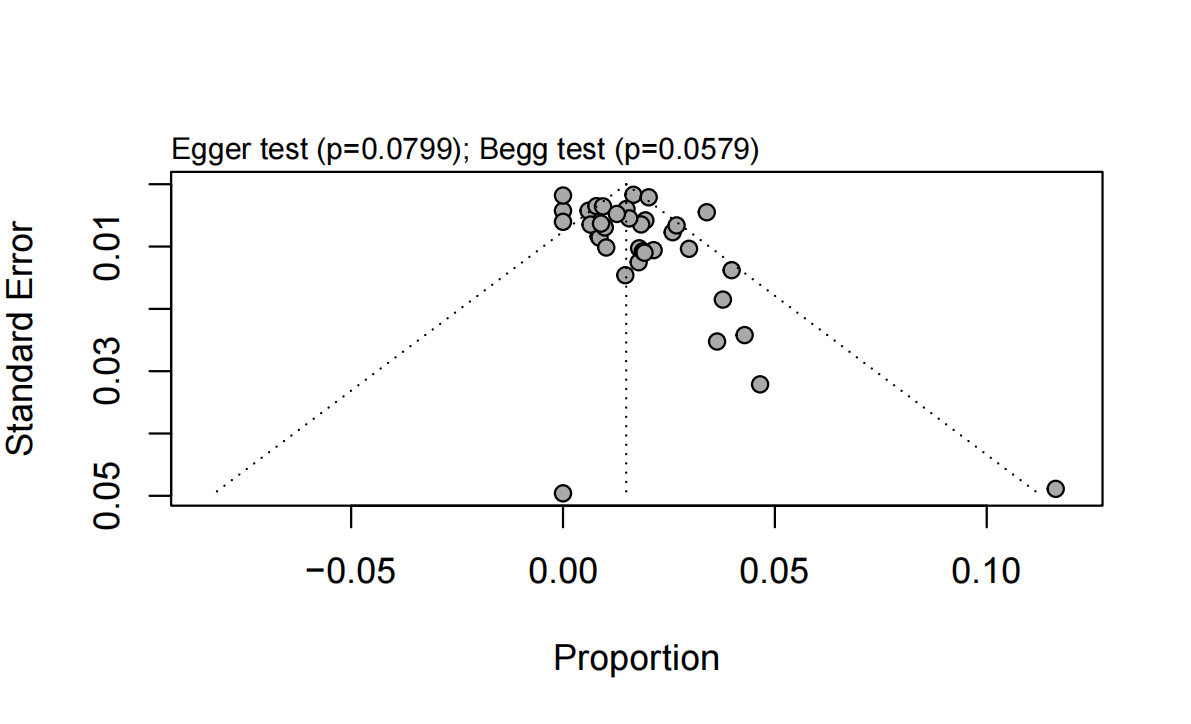


Figure S15 Funnel plot of prevalence of HPV 31 among male patients with genital warts


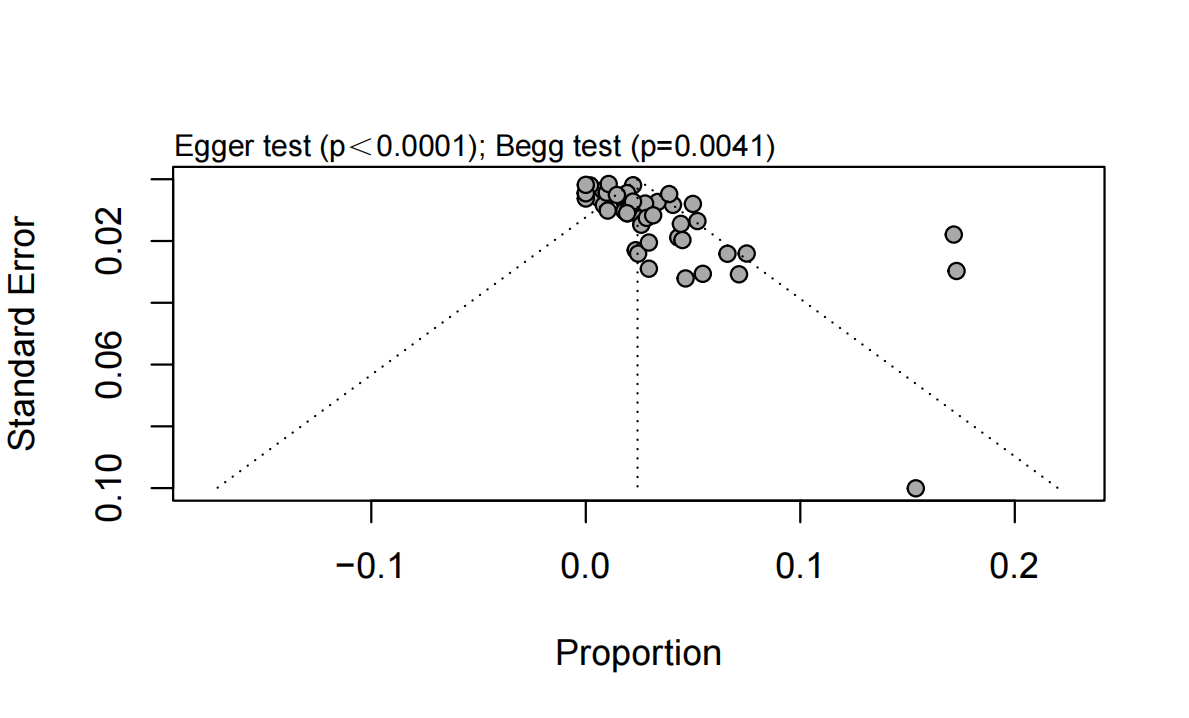


Figure S16 Funnel plot of prevalence of HPV 33 among male patients with genital warts


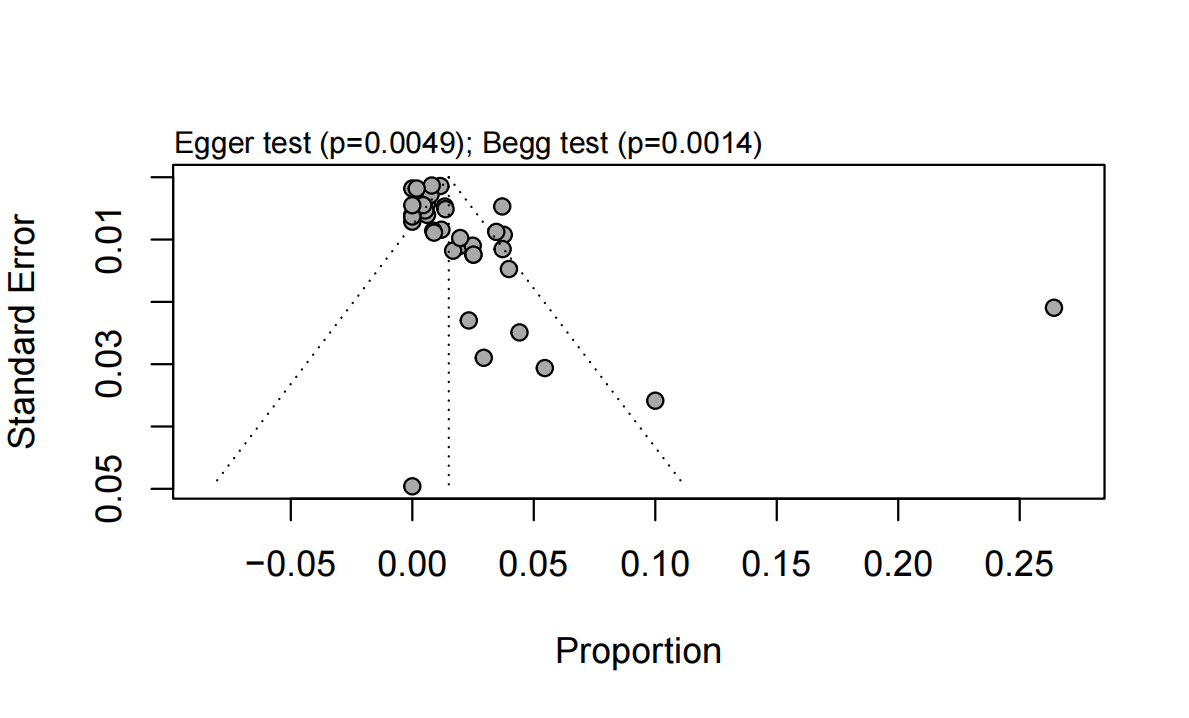


Figure S17 Funnel plot of prevalence of HPV 35 among male patients with genital warts


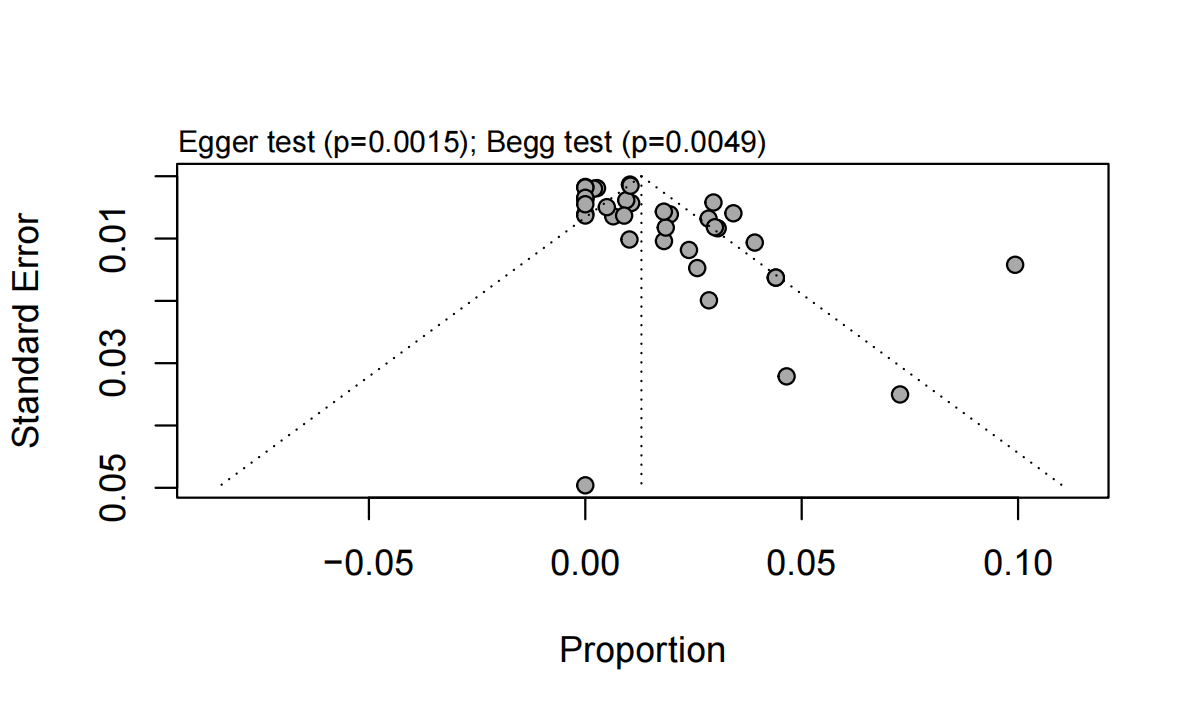


Figure S18 Funnel plot of prevalence of HPV 39 among male patients with genital warts


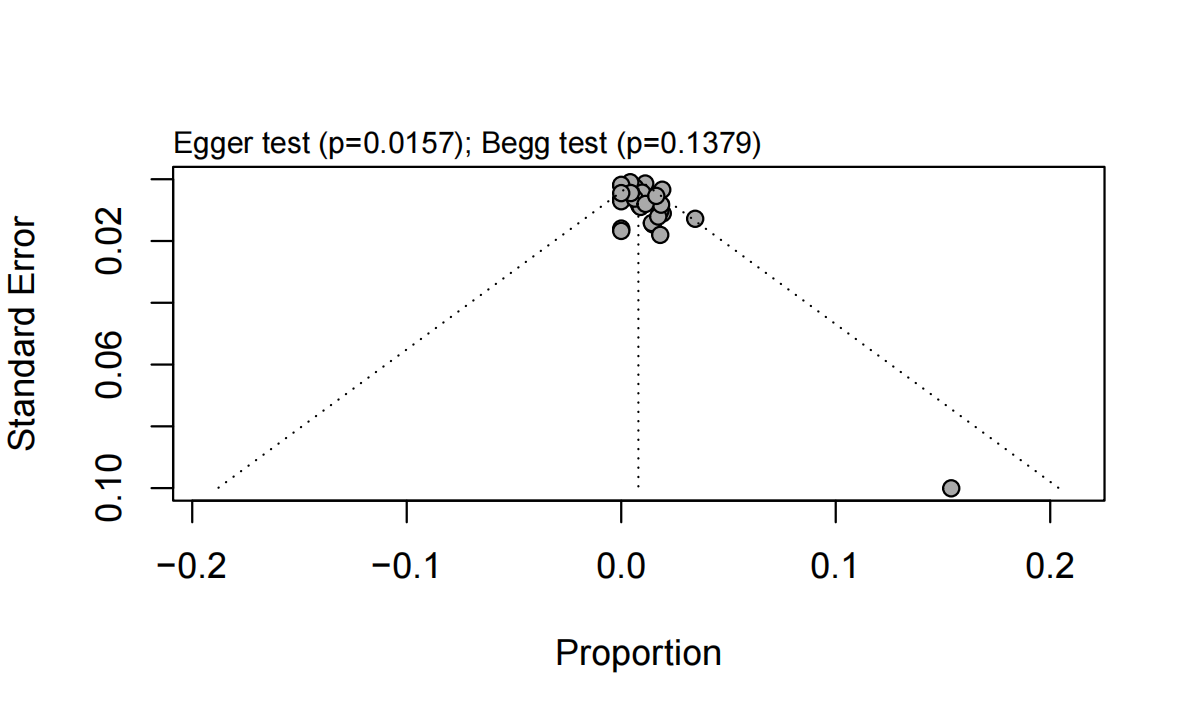


Figure S19 Funnel plot of prevalence of HPV 45 among male patients with genital warts


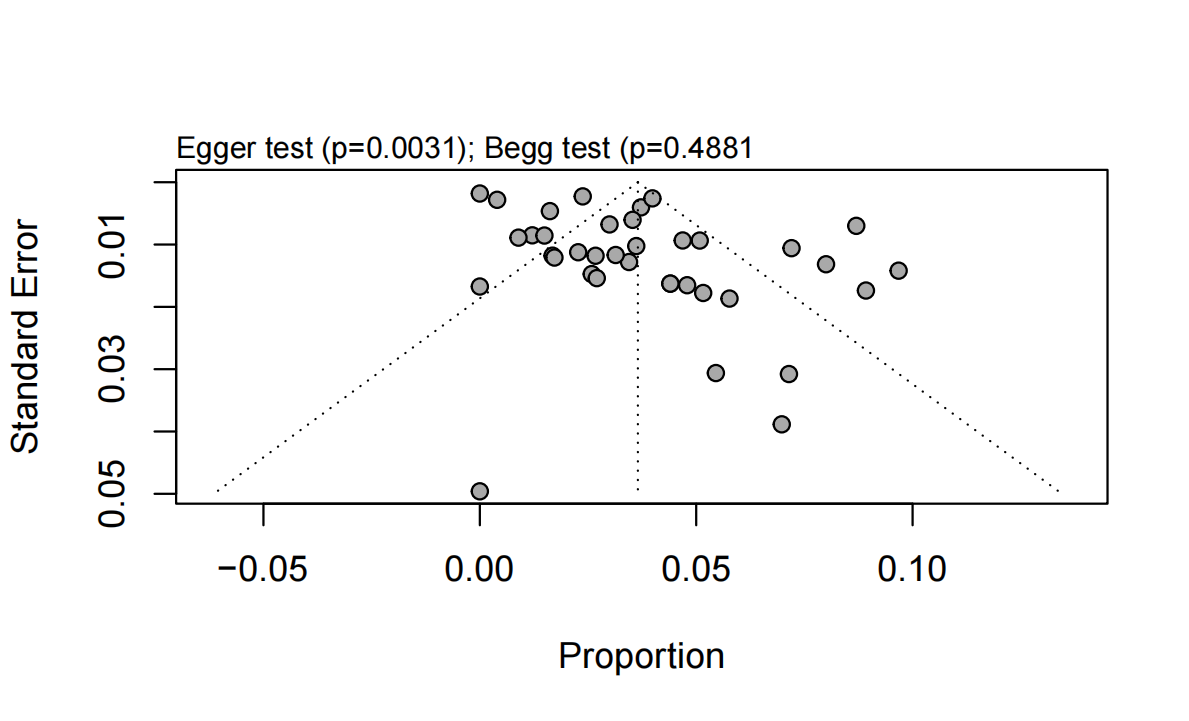


Figure S20 Funnel plot of prevalence of HPV 51 among male patients with genital warts


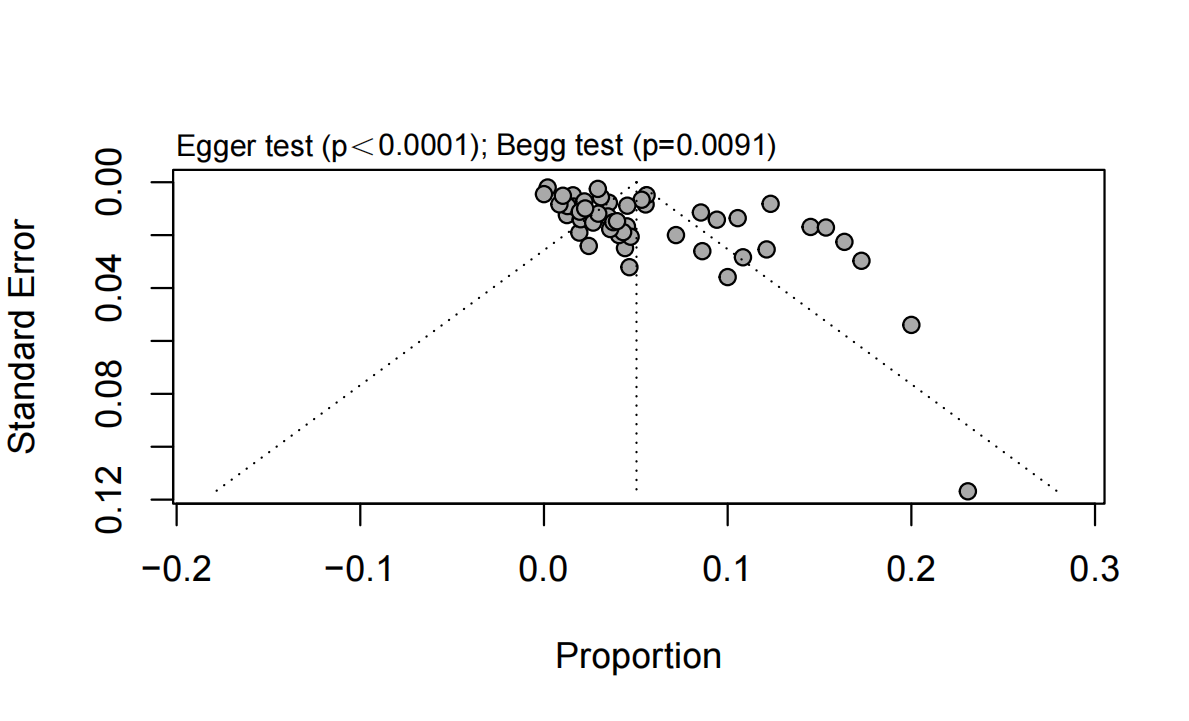


Figure S21 Funnel plot of prevalence of HPV 52 among male patients with genital warts


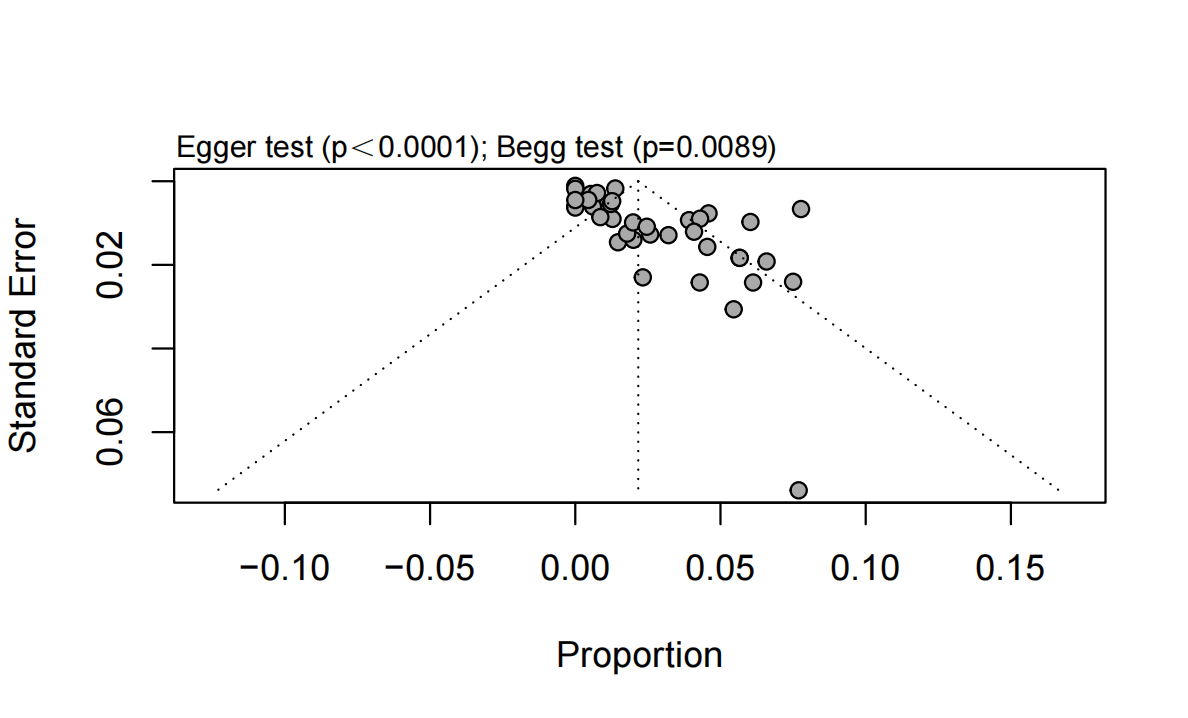


Figure S22 Funnel plot of prevalence of HPV 56 among male patients with genital warts


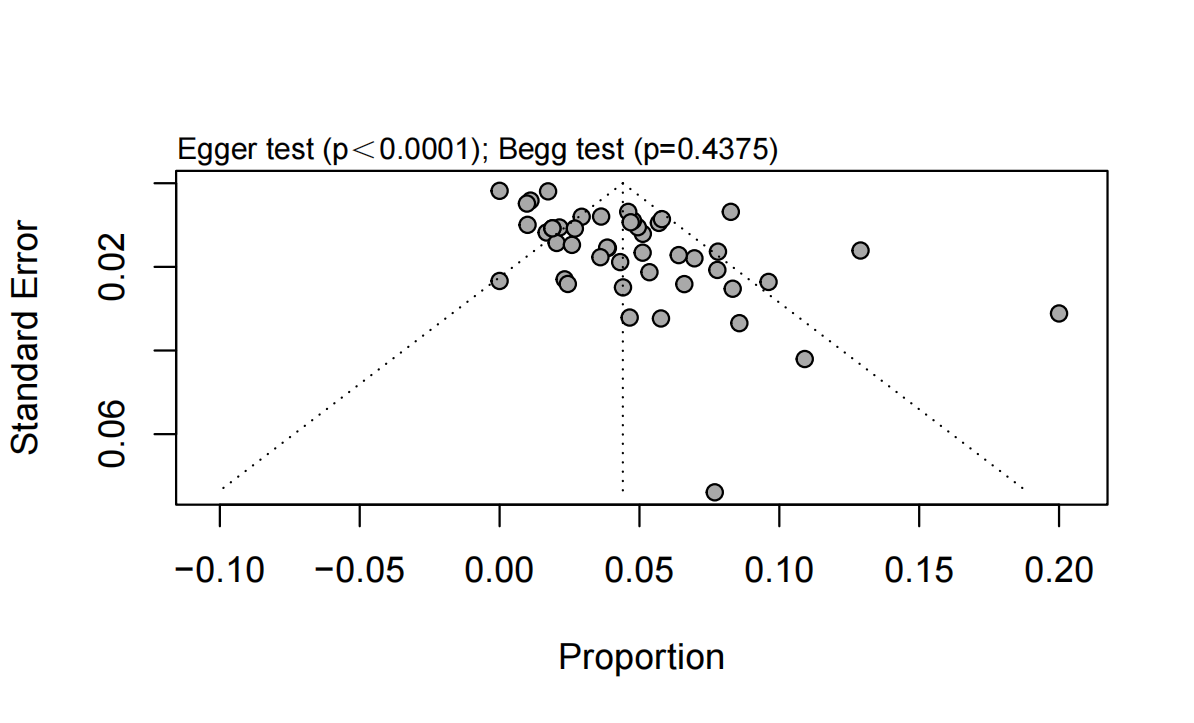


Figure S23 Funnel plot of prevalence of HPV 58 among male patients with genital warts


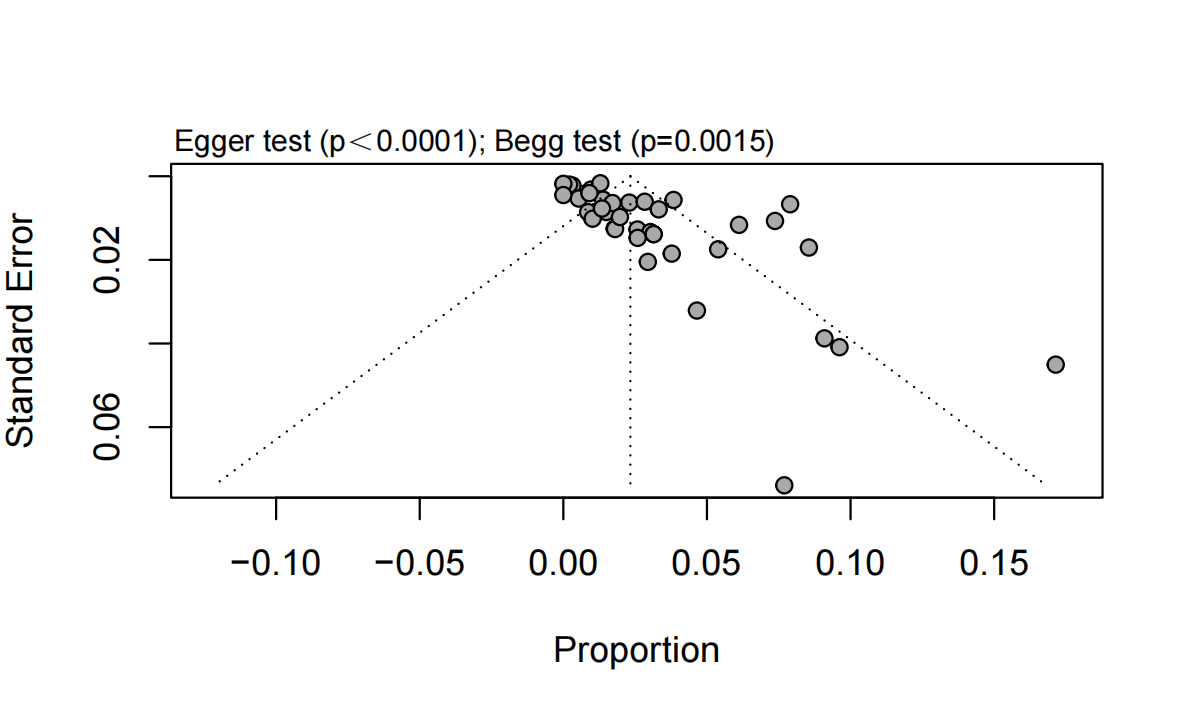


Figure S24 Funnel plot of prevalence of HPV 59 among male patients with genital warts


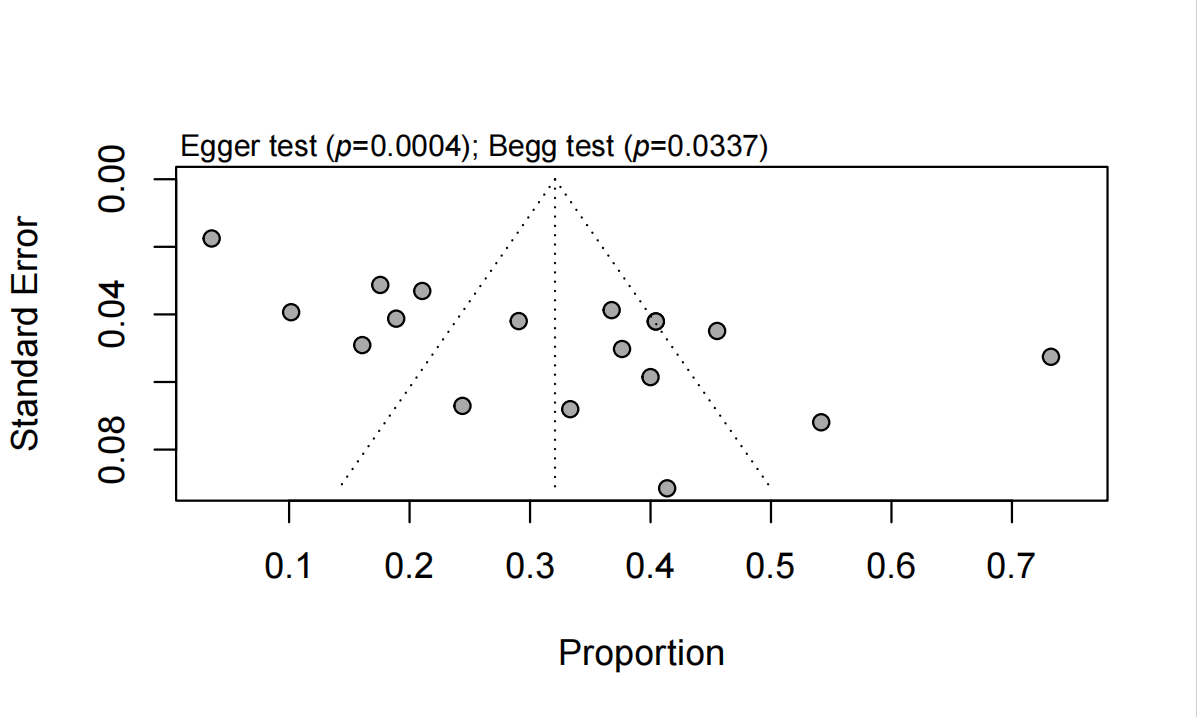


Figure S25 Funnel plot of prevalence of any type of HPV among male patients with esophageal cancer


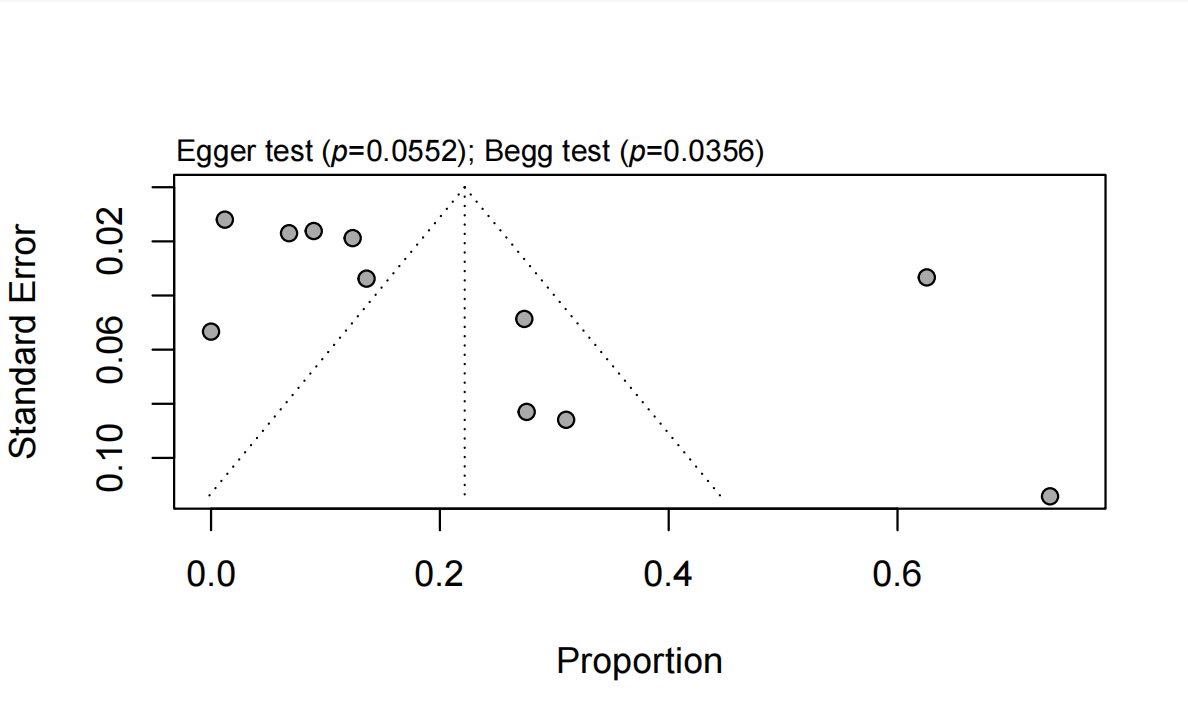


Figure S26 Funnel plot of prevalence of any type of HPV among male patients with laryngeal cancer


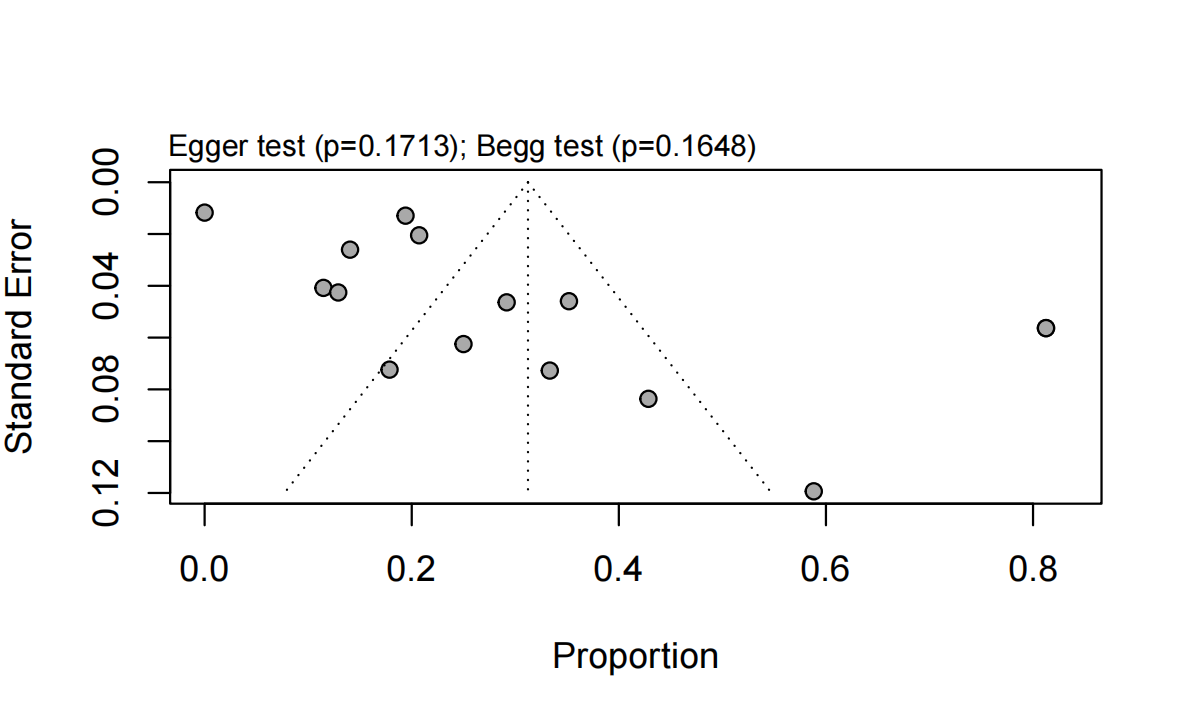


Figure S27 Funnel plot of prevalence of any type of HPV among male patients with oral cancer
